# Patient-reported outcome measures (PROMs) capturing therapy adherence of cancer patients: an umbrella review

**DOI:** 10.1080/1179271X.2026.2669130

**Published:** 2026-05-12

**Authors:** Luise Richter, Sophie Pauge, Wolfgang Greiner

**Affiliations:** a Methods in Empirical Social Research, Institute of Sociology, Faculty of Arts, Humanities and Social Science, Technische Universität Dresden, Dresden, Germany; b Department for Health Economics and Health Care Management, School of Public Health, Bielefeld University, Bielefeld, Germany

**Keywords:** Oncology, validation, therapy adherence, medication, diet, exercise

## Abstract

**Background:**

Adherence is a key determinant of treatment success in oncology and, according to the World Health Organization (WHO), describes how closely patients follow the recommendations agreed upon with their healthcare provider, including taking medications, adhering to dietary plans, or making lifestyle changes. Patient-reported outcome measures (PROMs) provide insights into patients’ perceptions, barriers, and beliefs. However, a comprehensive overview of adherence-related PROMs in oncology is lacking.

**Methods:**

We conducted a systematic umbrella review in PubMed and Embase up to October 2024 in adult cancer patients. A qualitative synthesis was performed to classify adherence dimensions according to the WHO framework and to evaluate the validation status.

**Results:**

Of 6,073 records screened, 89 systematic literature reviews (SLR) were included, encompassing 599 PROM applications. Most PROMs assessed medication adherence (41.1%). 4.8% of PROMs measured multiple adherence dimensions. 49.7% of PROMs were validated, while for 39.7% of PROMs validation status was not reported.

**Conclusion:**

Adherence-related PROMs in oncology predominantly focus on medication, while lifestyle and multidimensional aspects remain underrepresented. Validation status is frequently unclear. Standardized and validated modular PROMs are needed to inform the choice of PROMs for monitoring patient adherence in clinical practice and to enhance health outcomes.

## Introduction

Cancer remains a major public health challenge worldwide, with an increasing number of therapeutic options aiming to improve patient outcomes. Beyond advances in surgery, pharmacological therapy, and supportive care, the growing recognition of cancer as a chronic disease emphasizes holistic and interdisciplinary approaches that integrate patient engagement and self-management. Within this broader perspective, adherence has emerged as a central determinant of treatment success.

Adherence has traditionally been defined as “the extent to which the patient follows medical instructions.”^1^ While medication adherence is widely recognized as a key component of treatment success, particularly in oncology, where it is closely linked to clinical outcomes,^2^ this definition is too narrow to capture the full spectrum of interventions used in chronic disease management. In response, the WHO Adherence Project defined adherence as a multidimensional phenomenon: “the extent to which a person’s behavior - taking medication, following a diet, and/or executing lifestyle changes - corresponds with agreed recommendations from a health care provider.”^1^

Across chronic diseases in developed countries, average adherence rates were estimated by the WHO in 2002 to be around 50%, highlighting the magnitude of the challenge.^1^ Two decades later, adherence rates remain largely unchanged, with recent estimates in patients with multiple chronic conditions ranging from approximately 44% (based on electronic monitoring) to 77% (based on self-reports).^3^ Non-adherence is also a significant issue in oncology. For example, an SLR by Lasala and Santoleri^4^ reported non-adherence rates for oral anticancer therapies ranging from 16% to 100% across 42 studies. High adherence has been consistently associated with improved progression-free survival, reduced recurrence, and better overall survival, whereas non-adherence can impair treatment efficacy, disease control, and increase mortality risk.^4–7^ In addition to clinical outcomes, poor adherence entails substantial economic consequences, including increased healthcare utilization, hospitalizations, and productivity losses among cancer survivors.^8^ Therefore, improving adherence is not only vital for optimizing individual patient outcomes but also for alleviating the broader societal and economic burden of cancer care.

Non-adherence in oncology is a multifactorial phenomenon influenced by treatment complexity, side effects, symptom burden, and competing life demands. This complexity is reflected in behavioral models such as the Health Belief Model, which posits that health behaviors are shaped by perceived disease severity and susceptibility, as well as perceived benefits and barriers to treatment and self-efficacy,^9^^,^^10^ with strong empirical support from meta-analytic research.^11^ In addition, factors such as motivation, treatment beliefs, and the patient-provider relationship further influence adherence, highlighting that it is a context-dependent behavioral process.

Given these implications, accurate measurement of adherence is essential to identify patients at risk and to guide targeted interventions. Methods range from objective indicators, such as pill counts, pharmacy refill records, or electronic monitoring, to PROMs, which capture patients’ individual perspectives, beliefs, and barriers. While PROMs are widely applied in chronic diseases such as diabetes, heart failure, or HIV to monitor adherence and inform care,^12–14^ their use in oncology is becoming more relevant as cancer is increasingly recognized as a chronic disease. However, a comprehensive and systematic overview of PROMs across different adherence domains in oncology is currently lacking.

While the WHO framework supports a multidimensional understanding, adherence in non-pharmacological domains differs conceptually and operationally from medication adherence. Medication adherence typically refers to following prescribed dosing regimens (e.g. timing, dosage, persistence), whereas diet, exercise, and lifestyle-related measures are often assessed as behavioral patterns and may not always reflect adherence to clearly defined therapeutic prescriptions. Adherence is inherently a behavioral construct and can be assessed through reported behaviors; however, to capture adherence, these behaviors must be referenced to a specific recommendation. Otherwise, it may remain unclear whether the measured behavior reflects adherence or habitual lifestyle, potentially compromising construct validity. In this review, we therefore use the WHO framework as a guiding structure while explicitly acknowledging these conceptual differences.

In this context, the measurement of adherence becomes particularly challenging. Instruments that fail to capture the multifaceted and context-dependent nature of adherence may overlook important aspects of patient behavior. This underscores the importance of structured self-reported measures that enable systematic and comparable assessment across individuals.

To address this gap, we conducted an umbrella review to map PROMs used for assessing adherence in oncology. Using the WHO adherence framework as an a priori classification (medication, diet, exercise, lifestyle, other, multiple dimensions), our aims were to (1) identify which adherence dimensions the identified PROMs capture, (2) assess the validation status of the applied PROMs, and (3) highlight gaps and priorities for future development and validation of adherence measures. This synthesis seeks to inform healthcare providers, researchers, and policymakers, supporting the design of targeted interventions, strengthening patient education, and ultimately improving adherence and patient outcomes while reducing the societal burden of cancer care.

## Material and methods

To comprehensively capture the complexity of different areas of adherence to cancer therapies and efficiently manage the large volume of literature, we conducted an umbrella review. This approach allows the synthesis of existing evidence by identifying relevant studies through SLRs. The review was conducted in accordance with the Preferred Reporting Items for Overviews of Reviews (PRIOR) guidelines.^15^

### Eligibility criteria

We included peer-reviewed SLRs published in English or German. The target population comprised adult cancer patients (≥18 years) either undergoing treatment or in survivorship. Cancer therapy was broadly defined as any intervention within the holistic cancer care pathway, encompassing medical, supportive, and behavioral components, without restrictions regarding cancer type or stage.

Eligible SLRs assessed adherence to cancer therapy, regardless of whether a meta-analysis was conducted, and reported at least one primary study that used structured PROMs to evaluate adherence to cancer therapy or related side effects. Structured PROMs were defined as instruments based on a predefined set of items or formats applied consistently across participants. No minimum psychometric thresholds were applied, as the aim was to map the range of measures.

We excluded scoping reviews and SLRs focusing exclusively on adherence in preventive contexts or on guideline adherence by healthcare professionals. Furthermore, qualitative approaches, such as semi-structured interviews, were excluded, as they do not rely on a fixed set of items and therefore do not allow for systematic comparison across individuals. To focus on real-world and survivorship contexts and to avoid overrepresentation of highly controlled, protocol-driven settings, we excluded phase I–III randomized pharmaceutical trials. In these settings, adherence is typically assessed under conditions that differ substantially from routine clinical practice (e.g. strict monitoring and selected patient populations).

### Information sources and search strategy

A comprehensive systematic literature search was conducted in PubMed and Embase from inception to October 2024. The search strategy was developed collaboratively and combined synonyms and related terms for cancer, adherence/compliance, and systematic reviews to ensure broad identification of relevant literature, based on established taxonomies. For analysis, all identified measures were classified according to the WHO adherence framework, which served as the a priori conceptual reference. The detailed search strategy is provided in Supplementary Table S1. Title/abstract and full-text screening were performed independently by two authors (LR and SP) based on predefined eligibility criteria. Any disagreements were resolved by consensus.

### Quality appraisal

To assess the methodological quality of the included SLRs, we applied the validated AMSTAR 2 tool, which is specifically designed to appraise SLRs of both randomized and non-randomized healthcare interventions. The tool evaluates SLRs across 16 domains, seven of which are considered critical. Based on the presence of critical and non-critical methodological weaknesses, the overall confidence in each review was categorized as high, moderate, low, or critically low.^16^ AMSTAR 2 ratings were used descriptively to characterize the methodological quality of the included SLRs. No quality-based weighting or restrictions were applied, as the aim was to map the range of reported instruments rather than to conduct a quality-weighted synthesis.

### Data extraction and qualitative synthesis

Data extraction was performed independently by two authors (LR and SP) using a structured, purpose-built Excel template. The following information was extracted:

- Publication details: author, year, title, time frame of the SLR
- SLR characteristics: whether a meta-analysis was performed (yes/no), healthcare setting, study designs, number of primary studies included, cancer entities, treatment stage (currently under treatment and/or in survivorship), total number of cancer patients included, and whether study quality was assessed (yes/no)
- Results: adherence outcomes, adherence dimensions reported by the SLR, number and names of PROMs used, adherence dimensions assessed by PROMs, and validation status (validated, adapted, not validated, not reported).

Validation status was categorized based on the information reported in the included SLRs and primary studies. Importantly, validation encompasses multiple aspects (e.g. content, construct, and cross-cultural validity). The applied categorization provides a high-level overview and does not capture the full complexity of psychometric evaluation. Differences in validation type and quality should therefore be interpreted with caution.

To analyze adherence domains, therapy types were classified according to the WHO adherence framework (medication, diet, lifestyle, exercise, other). An additional category, “multiple,” was added for cases in which several adherence dimensions were assessed simultaneously. Because the aims and reporting quality of SLRs varied, we also reviewed primary studies cited in the included SLRs when necessary to obtain more detailed information on PROMs. Since our umbrella review required that SLRs explicitly reported the use of PROMs to assess adherence, only those primary studies in which a PROM was clearly identified were examined in detail.

## Results

**Figure 1.**
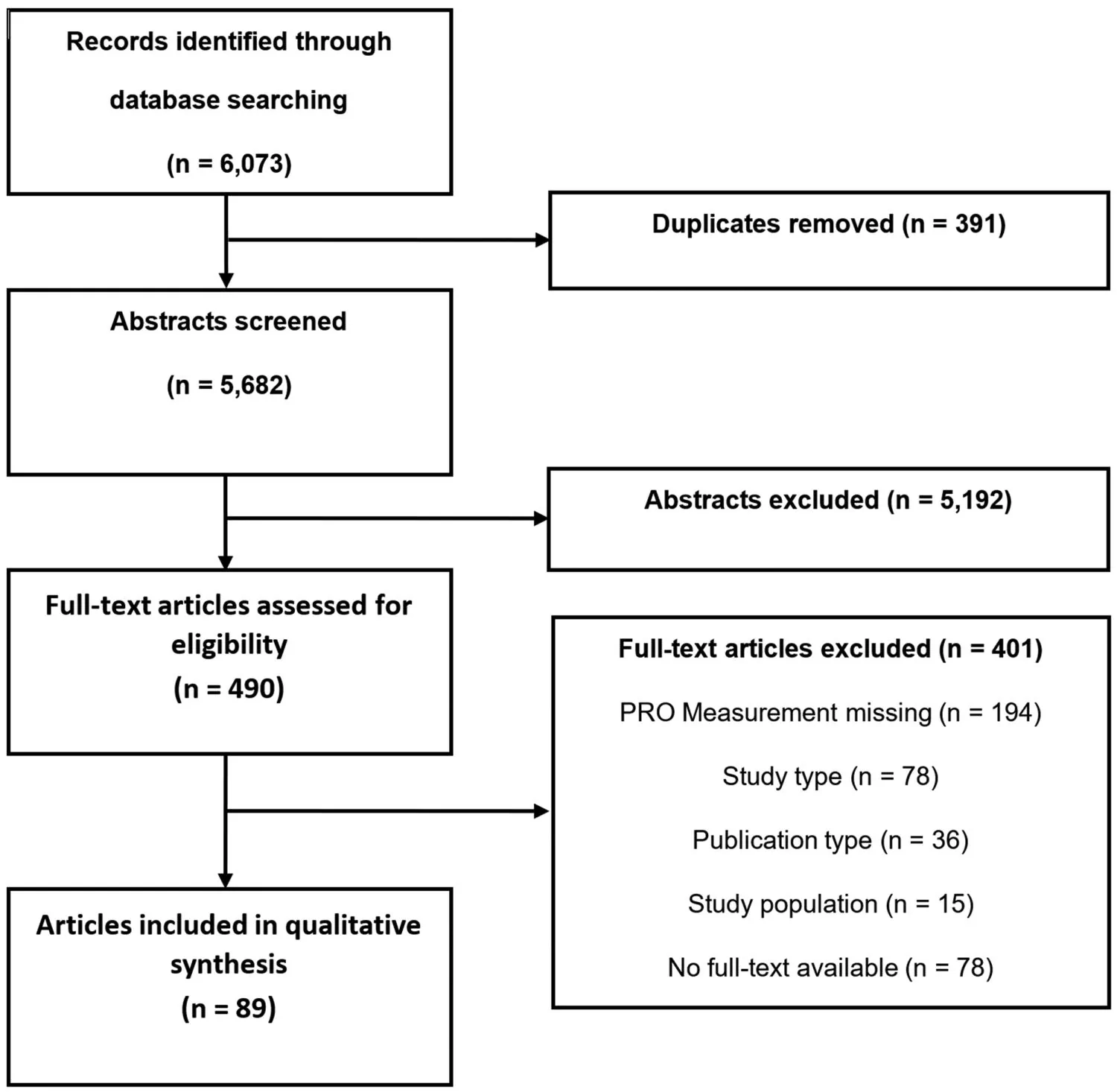
Flow chart of study selection process.

A total of 6,073 records were identified up to October 2024. After removing 391 duplicates, 5,682 articles remained, of which 5,192 articles were excluded after abstract screening. During screening of the remaining 490 full texts, 401 articles were excluded. Most full-text articles were excluded during the screening process due to the absence of a PRO measurement (*n* = 194), or a not eligible study or publication type (n = 78 and 36, respectively). Ultimately, 89 SLRs met the predefined criteria and were included (See Figure 1 for the flow chart of study selection).

### Characteristics of included SLRs

Table 1 summarizes the characteristics of the 89 included SLRs. The majority of SLRs (61; 68.5%) included studies from more than one continent, while three SLRs (3.4%) focused on a single continent (Europe, n = 2; North America, n = 1). Most SLRs (n = 46; 51.7%) included studies with multiple study designs, 20 (22.5%) were observational studies, 14 (15.8%) were randomized controlled trials, and 2 (2.3%) were non-randomized intervention studies.

**Table 1.** Summarized characteristics of included SLRs.

Category	n (%)
Number of reviews	89 (100%)
Number of studies included in SLR Mean/median sample size (range): 22.1/15.5 (2–146)	
<10	26 (29.2%)
10–19	30 (33.7%)
20–49	26 (28.2%)
≥ 50	7 (7.9%)
Number of studies using PROMs included in SLR Mean/median sample size (range): 8.4/5 (1–54)	
<5	39 (43.8%)
5–9	25 (28.1%)
10–19	17 (19.1%)
≥20	8 (9.0%)
Number of patients included (total) Mean/median sample size (range): 94,937/3,541 (168–2,666,401)	
<1,000	22 (24.7%)
1,000–4,999	27 (31.3%)
5,000–19,999	12 (13.5%)
20,000–99,999	16 (18.0%)
≥100,000	9 (10.1%)
Not reported	3 (3.4%)
Meta-analysis performed	
Yes	20 (22.5%)
No	69 (77.5%)
Healthcare setting	
Multicontinental	61 (68.5%)
Europe	2 (2.3%)
North America	1 (1.1%)
Not reported	25 (28.0%)
Study design	
Multimethod studies	46 (51.7%)
Observational studies	20 (22.5%)
Randomized controlled trials (RCTs)	14 (15.8%)
Non-randomized intervention studies	2 (2.3%)
Not reported	7 (7.9%)
Treatment stadium	
Currently under treatment	58 (65.2%)
Survivor	12 (13.5%)
Currently under treatment and survivor	19 (21.3%)
Cancer entities	
Various cancer entities	38 (42.7%)
Breast cancer	25 (28.1%)
Gynecological cancers	3 (3.4%)
Colorectal/gastrointestinal cancers	5 (5.6%)
Lung cancer	2 (2.3%)
Prostate cancer	1 (1.1%)
Head and Neck Cancer	4 (4.5%)
Glioblastoma/brain tumors	0 (0%)
Hematologic malignancies	7 (7.9%)
Thyroid cancer	0 (0%)
Not reported	4 (4.5%)

Regarding the number of studies included per SLR, the median was 15.5 (mean 22.1; range 2–146), with most SLRs including fewer than 20 studies. The median for the number of studies using PROMs per SLR was 5. The total number of patients included across SLRs varied widely (median 3,541; mean 94,937; range 168–2,666,401). Most reviews included fewer than 5,000 patients, while nine reviews (10.1%) included more than 100,000 patients.

Most SLRs focused on patients currently undergoing treatment (58; 65.2%), whereas 12 (13.5%) focused on survivors, and 19 SLRs (21.3%) included both patients under treatment and survivors. Regarding cancer type, the majority of SLRs included mixed cancer populations (38; 42.7%), followed by breast cancer (25; 28.1%) (see Supplementary Table S2 for detailed characteristics of included SLRs).

### Quality appraisal

Overall, according to the AMSTAR 2 tool, 57 reviews were rated as (critically) low and 32 as moderate in methodological quality. The appraisal revealed variability in methodological rigor: while a number of reviews adhered to key standards of systematic review methodology, such as employing an adequate search strategy and conducting the study selection process in duplicate, overall confidence remained low across the body of evidence. This was primarily attributed to the frequent absence of a comprehensive justification for the selection of study designs, insufficient reporting of excluded studies, and limited use of appropriate strategies to address the risk of bias in included publications - either through formal quality appraisal, critical interpretation of findings, or disclosure of underlying funding sources. A detailed overview of the quality appraisal for each review is provided in Supplementary Table S3.

### Dimensions of patient-reported adherence

Across the 89 SLRs, medication adherence was the dimension most frequently addressed, reported in 52 SLRs (58.4%). Exercise adherence was addressed in 17 SLRs (19.1%) and dietary adherence in 12 SLRs (13.5%), while lifestyle-related adherence was rarely covered (2 SLRs, 2.3%). Other heterogeneous adherence domains were captured in 6 SLRs (6.7%).

Across the included SLRs, we identified 510 unique primary studies employing at least one PROM to assess adherence. These were derived from 758 records, of which 248 represented duplicates across reviews. Among the identified primary studies, 63 studies applied more than one PROM, most commonly two instruments (n = 45), resulting in a total of 599 PROM applications.

Table 2 summarizes the adherence dimensions captured by the identified PROM applications. Most PROMs assessed medication adherence (n = 246), followed by dietary adherence (n = 222) and exercise adherence (n = 82). Fewer instruments addressed lifestyle-related adherence (n = 6). An additional 14 instruments were categorized as “Other”, covering dilator use (n = 11) and meditation or mindfulness practice (n = 3). Due to their small number, these were grouped into a single “Other” category despite representing conceptually distinct domains. Moreover, 29 instruments were classified as “Multiple”, assessing more than one adherence dimension. Most of these instruments assessed two dimensions (n = 25), predominantly diet and exercise (n = 13) or diet and lifestyle (n = 5). Given the small number of instruments and the heterogeneity of combinations, these were grouped into a single “Multiple” category for descriptive purposes. Overall, most instruments targeted a single adherence domain, with medication adherence being the most frequently assessed. A detailed description of all identified PROMs is provided in Supplementary Table S4.

**Table 2.** Dimensions of adherence.

Adherence dimension	PROMs used n (%)	Validated n (%)	Adapted n (%)	Not validated n (%)	Validation status not reported n (%)
Medication	246 (100%)	130 (52.8%)	28 (11.4%)	11 (4.5%)	77 (31.3%)
Diet	222 (100%)	146 (65.4%)	19 (8.5%)	0 (0.0%)	57 (25.5%)
Exercise	82 (100%)	16 (19.5%)	1 (1.2%)	0 (0.0%)	65 (79.3%)
Lifestyle	6 (100%)	2 (33.3%)	1 (16.7%)	0 (0.0%)	3 (50.0%)
Other	14 (100%)	0 (0.0%)	0 (0.0%)	1 (7.1%)	13 (92.9%)
Multiple	29 (100%)	4 (13.8%)	1 (3.4%)	1 (3.4%)	23 (79.3%)
Total	599 (100%)	298 (49.7%)	50 (8.3%)	13 (2.2%)	238 (39.7%)

Overall, 49.7% of the identified PROMs were validated (n = 298), and an additional 8.3% (n = 50) represented adaptations of validated instruments. 2.2% (n = 13) were explicitly reported as not validated, while for 39.7% (n = 238) the validation status was not reported or could not be determined from the available sources. The distribution varied substantially across adherence dimensions. For medication-related PROMs, 52.8% were validated, with 31.3% lacking validation status. Dietary PROMs showed the highest proportion of validated instruments (65.4%), while most exercise- and lifestyle-related PROMs did not report validation status (79.3% and 50.0%, respectively). These results indicate that, despite the large number of PROMs identified, evidence regarding validation remains limited in several adherence dimensions, particularly for exercise, lifestyle, and heterogeneous categories.

### Applied PROMs

Table 3 summarizes the most frequently applied PROMs across adherence dimensions. Within the medication domain, the Morisky Medication Adherence Scale (MMAS, n = 78) was most used, followed by non-specified questionnaires (n = 62) and the Medication Adherence Rating Scale (MARS, n = 22). Dietary adherence was most frequently assessed using Food Frequency Questionnaires (n = 73), Mediterranean Diet Adherence Scores (MEDAS, n = 38), and the Healthy Eating Index (HEI, n = 30). For exercise adherence, diaries (n = 63) dominated, whereas standardized instruments such as the 7-day Physical Activity Recall (7-PAR, n = 6) and the Godin Leisure-Time Physical Activity Questionnaire (GLTPAQ, n = 4) were used less often. Finally, mixed instruments covering more than one adherence dimension primarily consisted of diaries (n = 14), non-specified questionnaires (n = 10), and Food Frequency Questionnaires (n = 2). For lifestyle-related and other adherence dimensions, only a few PROMs were identified (n = 6 and 14, respectively).

**Table 3.** Measurement instruments capturing adherence are most frequently used by dimension.

Adherence dimension	Instrument	PROMs used n (%)	Validated n (%)	Adapted n (%)	Not validated n (%)	Validation status not reported n (%)
Medication	MMAS*	78 (100%)	58 (74%)	20 (26%)	0 (0%)	0 (0%)
	Questionnaire, not specified	62 (100%)	9 (15%)	2 (3%)	8 (13%)	43 (69%)
	MARS*	22 (100%)	22 (100%)	0 (0%)	0 (0%)	0 (0%)
Diet	FFQ	73 (100%)	40 (54%)	13 (18%)	0 (0 %)	20 (27%)
	Mediterranean Diet Adherence Scores*	38 (100%)	37 (97%)	1 (3%)	0 (0%)	0 (0%)
	Healthy Eating Indices*	30 (100%)	30 (100%)	0 (0%)	0 (0%)	0 (0%)
	Diary	27 (100%)	0 (0%)	0 (0%)	0 (0%)	27 (100%)
Exercise	Diary	63 (100%)	0 (0 %)	0 (0%)	0 (0%)	63 (100%)
	7-day Physical Activity Logs*	6 (100%)	6 (100%)	0 (0%)	0 (0%)	0 (0%)
	GLTPAQ	4 (100%)	3 (75%)	1 (25%)	0 (0%)	0 (0%)
Multiple	Diary	14 (100%)	0 (0%)	0 (0%)	0 (0%)	14 (100%)
	Questionnaire, not specified	10 (100%)	1 (10%)	0 (0%)	0 (10%)	9 (80%)
	FFQ	2 (100%)	1 (50%)	1 (50%)	0 (0%)	0 (0%)

Abbreviations. MMAS, Morisky Medication Adherence Scale; MARS, Medication Adherence Report Scale; FFQ, Food Frequencies Questionnaire; GLTPAQ, Godin Leisure Time Physical Activity Questionnaire.

*Note*. Due to their infrequent use, some PROMs are not displayed here; *Instruments were applied in different versions (e.g. short and long forms). These versions were summarized in the table. A comprehensive list, including all PROMs identified as well as detailed information on specific versions, is provided in Supplementary Table S4. Duplicate records of primary studies reported across multiple SLRs were identified and removed prior to analysis to avoid double-counting. Proportions are therefore based on unique PROM applications.

Across all domains, validation status varied considerably. While established tools such as MARS, the Healthy Eating Index, or the Mediterranean Diet Adherence Scores were consistently validated, non-specified questionnaires and diaries frequently lacked validation. A comprehensive list of identified PROMs and validation status can be found in Supplementary Table S4.

## Discussion

This umbrella review provides a comprehensive overview of PROMs used to assess adherence among cancer patients, and, to our knowledge, it is the first to capture PROMs that address adherence across all adherence dimensions. We found that most instruments focused on medication or dietary adherence, whereas the dimensions of exercise and lifestyle were underrepresented. Moreover, adherence was rarely conceptualized in a multidimensional way, despite the fact that oncology care often requires patients to manage complex treatment regimens across multiple domains. This gap reflects an important challenge in the current measurement landscape and suggests that many existing PROMs only capture a partial picture of patients’ adherence behavior.

The predominance of medication adherence measures should be interpreted in light of both structural differences between adherence domains and historical developments in oncology care. Medication adherence, particularly to oral therapies such as endocrine treatments or tyrosine kinase inhibitors, is clinically central, well-defined, and closely linked to treatment outcomes.^17^^,^^18^ In contrast, non-pharmacological components such as diet, exercise, or lifestyle have only more recently gained attention as part of more holistic treatment approaches. These are often less clearly defined, more heterogeneous, and often intervention-specific, which makes measurement more challenging and may partly explain their lower representation.

Another key finding relates to psychometric quality. While half of the identified PROMs had undergone formal validation, a substantial proportion lacked information on validation status, with considerable variation across adherence dimensions, being highest for dietary PROMs and lowest for exercise-related measures. Instruments with limited or unknown psychometric properties may undermine the reliability and comparability of adherence data across studies and clinical practice. If the measurement tools themselves have unclear or insufficient psychometric properties, conclusions drawn from studies using these instruments must be interpreted with caution. At the same time, this pattern may partly reflect incomplete reporting of validation in the included SLRs and primary studies rather than a complete absence of validation.

Furthermore, diaries and logs, particularly in the context of exercise or behavioral interventions, were frequently used but classified as “not validated” due to the absence of formal psychometric evaluation as PROMs. While these tools can provide detailed and context-specific insights into patient behavior, they differ from validated PROMs in their level of standardization and contribute, together with non-specified questionnaires, to conceptual heterogeneity within the identified measures.

Our findings align with evidence from a recent SLR by Kwan et al.^19^ which focused solely on medication adherence and identified 121 unique PROMs across chronic conditions. Despite the large number of available tools, the authors reported that only a few instruments demonstrated consistent evidence for key measurement properties such as construct validity or internal consistency. Like Kwan et al., we found the Morisky Medication Adherence Scales (MMAS) and the Medication Adherence Rating Scales (MARS) to be among the most frequently used tools, alongside many unspecified, study-specific questionnaires. Kwan et al. additionally reported frequent use of the Adult AIDS Clinical Trials Group (AACTG) adherence items (AACTG).

However, the widespread use of generic adherence instruments such as MMAS and MARS may be limited in their applicability within the oncological context. These scales were originally developed for chronic disease populations with relatively stable treatment regimens and may not fully capture the complexity of adherence in cancer care. Oncology treatment often involves multimodal and dynamic regimens, including combinations of systemic therapies, supportive treatments, and frequent dose adjustments due to treatment-related toxicities. While the use of established instruments facilitates comparability across studies, their suitability for capturing oncology-specific adherence challenges remains uncertain. This highlights the need for oncology-specific or oncology-sensitive adherence measures. Taken together, these findings indicate that the frequent use of PROMs does not necessarily reflect their suitability or methodological quality. Summary counts should, therefore, be interpreted as reflecting the broader landscape of self-reported adherence measures across the included SLRs rather than the availability of validated PROMs.

For research, the substantial heterogeneity of measures limits comparability across studies. Future work should focus on developing and validating adherence measures tailored to oncology, taking into account feasibility, patient burden, and clinical relevance across different phases of the cancer trajectory. In this context, modular approaches that combine a shared conceptual framework with domain-specific subscales may offer a flexible and promising solution. Such instruments could allow for both comparability across domains and sufficient specificity within individual adherence dimensions. Our study provides a foundation for identifying overlapping as well as treatment-specific content across existing questionnaires for different dimensions.

This umbrella review has several limitations. First, our analysis was restricted to PROMs reported within SLRs and underlying primary studies, which may have led to the omission of instruments or validation details and may introduce reporting bias.

Second, the search strategy may not have captured all relevant studies. Adherence is described using various terms (e.g. compliance, persistence). Although these terms were included, relevant terminology may still have been missed. Moreover, SLRs addressing adherence as a secondary or peripheral outcome were not captured if adherence was not explicitly mentioned in the abstract.

Third, the application of the WHO adherence framework across heterogeneous domains may introduce conceptual ambiguity. In particular, instruments assessing diet, physical activity, or lifestyle may capture general health behaviors if not related to clearly defined therapeutic recommendations, potentially leading to overestimation of non-medication adherence domains.

Fourth, validation was categorized using a simplified classification scheme that does not capture differences in validation type or methodological rigor. Moreover, some instruments were developed and validated in non-oncologic populations, which may limit their applicability to cancer patients. In addition, no minimum psychometric thresholds for reliability or validity were applied, as the aim of this review was to map available measures rather than to evaluate their psychometric quality in detail. While this approach allowed for a comprehensive overview, it may introduce heterogeneity and should be considered when interpreting the findings.

Fifth, classifying diaries and logs as “not validated” may oversimplify their role. While often lacking formal psychometric evaluation, they are widely used and may capture context-specific aspects of adherence, but contribute to conceptual heterogeneity.

Finally, the methodological quality of the included reviews was often low or critically low. Consequently, the findings may be influenced by the quality of the included reviews.

## Conclusion

This umbrella review shows that most PROMs used to assess adherence in oncology focus on a single dimension, most often medication adherence, while multidimensional aspects of adherence remain underrepresented. This limits comparability across studies and may hinder a comprehensive understanding of patients’ adherence behavior in a holistic cancer treatment pathway. Future research should therefore prioritize the development and validation of standardized PROMs that reflect the complexity of cancer care. Modular approaches, combining a shared framework with domain-specific subscales, may offer a promising way to balance comparability across domains with sufficient precision within individual adherence dimensions.

## Supplementary Material

SM Table S2 SLR characterisctics.docx

Revised SM Table S4 PROMs.docx

SM Table S3 AMSTAR 2 Quality appraisal of included systematic reviews.docx

SM Table S1 Search Strategy 2.docx

## Transparency

### Declaration of financial/other relationships

The authors have no relevant affiliations or financial involvement with any organization or entity with a financial interest in or financial conflict with the subject matter or materials discussed in the manuscript. This includes employment, consultancies, honoraria, stock ownership or options, expert testimony, grants or patents received or pending, or royalties. Peer reviewers on this manuscript have no relevant financial or other relationships to disclose.
