# Supplementary material for: Patient-reported outcome measures (PROMs) capturing therapy adherence of cancer patients: an umbrella review
Source: Patient Relat Outcome Meas. 2026 May 12;17(1):25–34. doi: 10.1080/1179271X.2026.2669130 (PMC13536962; doi:10.1080/1179271X.2026.2669130)
Supplement: Supplementary file 1 [file DPRO_A_2669130_SM6242.docx]

**Table S1 Search strategy for databases of Pubmed and Embase**

|  | **Population** | **Intervention** | **Study Design** |
| --- | --- | --- | --- |
| MeSH  (Pubmed) | Neoplasms | Treatment Adherence and Compliance | Systematic Review |
|  | Medical oncology |  |  |
|  |  |  |  |
|  |  |  |  |
|  |  |  |  |
| Emtree (Embase) | Oncology | Mental compliance | Systematic review |
|  | Neoplasm |  |  |
|  |  |  |  |
|  |  |  |  |
|  |  |  |  |
| Freetext  (Title/Abstract) | Cancer* | Adheren* | systematic review |
|  | Tumor* | Complian* | systematic literature |
|  | Oncolog* | Nonadheren* | Meta-analysis |
|  | Neoplasm* | Noncomplian* | systematic search |
|  | Carcinoma* | Refusal |  |
|  | Malignanc* | Discontinuation |  |
|  |  | Dropout* |  |
|  |  | Drop out* |  |
|  |  | Attendance |  |
|  |  | Persistance |  |
| Filter | English, German | | |
