# Supplementary material for: Patient-reported outcome measures (PROMs) capturing therapy adherence of cancer patients: an umbrella review
Source: Patient Relat Outcome Meas. 2026 May 12;17(1):25–34. doi: 10.1080/1179271X.2026.2669130 (PMC13536962; doi:10.1080/1179271X.2026.2669130)
Supplement: Supplementary file 2 [file DPRO_A_2669130_SM6244.docx]

**Table S3 AMSTAR 2 Quality appraisal of included systematic reviews**

| **Study** | **Overall** | **Q1** | **Q2*** | **Q3** | **Q4*** | **Q5** | **Q6** | **Q7*** | **Q8** | **Q9 RCTs*** | **Q9 NRSI*** | **Q10** | **Q11 RCTs*** | **Q11 NRSI*** | **Q12** | **Q13*** | **Q14** | **Q15*** | **Q16** |
| --- | --- | --- | --- | --- | --- | --- | --- | --- | --- | --- | --- | --- | --- | --- | --- | --- | --- | --- | --- |
| Abboud et al. 2021 | Moderate | Y | PY | N | PY | Y | Y | Y | Y | Y | NA | N | NA | NA | NA | Y | Y | NA | Y |
| Agirre-Elordui et al. 2024 | Low | Y | Y | N | PY | Y | Y | N | Y | Y | NA | N | Y | NA | Y | Y | Y | Y | Y |
| Ahmed & Loiselle 2023 | Critically low | Y | PY | N | PY | N | N | N | PY | No | No | N | NA | NA | NA | N | N | NA | Y |
| Alves et al. 2016 | Critically low | N | PY | N | PY | N | N | PY | PY | NA | No | N | NA | NA | NA | N | Y | NA | N |
| Atkinson et al. 2016 | Critically low | N | N | N | PY | Y | Y | PY | PY | NA | No | Y | NA | NA | NA | N | N | NA | N |
| Barnes et al. 2019 | Moderate | Y | Y | N | PY | Y | N | PY | Y | PY | NA | Y | NA | NA | NA | Y | Y | NA | Y |
| Baydoun et al. 2021 | Low | N | PY | N | PY | Y | Y | PY | Y | Y | NA | Y | NA | NA | NA | N | Y | NA | Y |
| Bright et al. 2023 | Critically low | Y | Y | Y | Y | Y | Y | N | Y | PY | PY | N | Y | Y | N | N | Y | N | Y |
| Bruns et al. 2016 | Moderate | N | PY | N | PY | Y | Y | PY | PY | Y | NA | N | NA | NA | NA | Y | Y | NA | Y |
| Burke et al. 2024 | Moderate | Y | Y | N | PY | Y | Y | N | Y | Y | Y | N | NA | NA | NA | Y | Y | NA | Y |
| Cahir et al. 2015 | Critically low | Y | Y | N | PY | Y | Y | N | Y | No | No | N | Y | Y | N | N | Y | N | Y |
| Carr & Rosengarten 2021 | Critically low | Y | N | N | Y | Y | Y | N | Y | No | No | N | NA | NA | NA | N | Y | NA | Y |
| Charters et al. 2024 | Critically low | N | N | Y | PY | Y | N | N | PY | PY | No | N | NA | NA | NA | N | Y | NA | Y |
| Chen et al. 2022 | Critically low | N | Y | Y | PY | Y | Y | N | PY | PY | PY | N | NA | NA | NA | N | Y | NA | Y |
| Chen et al. 2023 | Critically low | Y | Y | N | Y | Y | Y | N | Y | Y | Y | N | Y | Y | N | N | Y | Y | Y |
| Cheung et al. 2023 | Critically low | Y | N | N | PY | Y | Y | N | PY | Y | No | N | NA | NA | NA | N | Y | NA | Y |
| Chlebowski & Geller 2006 | Critically low | N | PY | N | N | N | N | N | PY | NA | No | N | NA | NA | NA | N | N | NA | N |
| Chow et al. 2024 | Moderate | Y | Y | N | PY | Y | Y | Y | Y | Y | NA | N | Y | NA | Y | Y | Y | Y | Y |
| Claros et al. 2019 | Moderate | Y | Y | N | PY | Y | Y | PY | N | NA | Y | N | NA | NA | NA | Y | Y | NA | Y |
| Deng et al. 2024 | Critically low | N | Y | N | PY | Y | Y | N | PY | NA | No | N | Y | Y | Y | Y | Y | Y | Y |
| Eliasson, 2012 | Critically low | N | PY | N | PY | N | N | PY | Y | NA | No | Y | NA | NA | NA | N | N | NA | Y |
| Finitsis et al. 2018 | Moderate | N | PY | N | PY | Y | Y | PY | Y | Y | Y | N | Y | Y | Y | Y | Y | Y | Y |
| Fleming et al. 2020 | Critically low | N | Y | Y | PY | Y | Y | N | PY | Y | Y | N | NA | NA | NA | N | N | NA | Y |
| Ganna et al. 2024 | Critically low | Y | N | N | PY | Y | Y | N | PY | No | Y | N | NA | NA | NA | Y | N | NA | Y |
| Greer et al. 2016 | Moderate | N | PY | N | PY | N | N | PY | Y | Y | Y | Y | NA | NA | NA | Y | N | NA | Y |
| Haddad et al. 2021 | Critically low | N | PY | N | PY | Y | Y | PY | Y | N | N | Y | NA | NA | NA | N | Y | NA | Y |
| Hajjar & Rezazadeh 2021 | Critically low | Y | N | N | N | N | N | PY | PY | NA | No | N | NA | NA | NA | N | N | NA | Y |
| Haynes et al. 2021 | Low | Y | PY | N | PY | N | N | PY | PY | NA | Y | Y | NA | Y | Y | Y | Y | N | Y |
| Heiney et al. 2018 | Low | N | PY | N | PY | Y | Y | N | PY | Y | Y | Y | NA | NA | NA | Y | N | NA | Y |
| Heiney et al. 2021 | Moderate | N | PY | N | PY | Y | Y | PY | PY | Y | Y | Y | NA | NA | NA | Y | N | NA | Y |
| Horne et al. 2013 | Moderate | N | PY | N | PY | N | N | PY | PY | NA | Y | Y | NA | Y | Y | Y | Y | Y | Y |
| Huang et al. 2016 | Critically low | N | PY | N | N | N | N | PY | PY | NA | No | Y | NA | NA | NA | N | N | NA | Y |
| Hurtado-de-Medoza et al. 2016 | Low | N | PY | N | PY | Y | Y | N | PY | NA | Y | N | NA | NA | NA | Y | N | NA | Y |
| Kaptein et al. 2021 | Critically low | N | PY | N | N | N | N | PY | Y | NA | No | N | NA | NA | NA | N | N | NA | Y |
| Kavookjian & Wittayanukorn 2015 | Moderate | N | Y | N | Y | N | N | PY | Y | Y | Y | N | NA | NA | NA | Y | Y | NA | N |
| Kearney et al. 2022 | Critically low | Y | Y | Y | PY | Y | Y | N | Y | Y | No | N | NA | NA | NA | N | Y | NA | Y |
| Krekeler et al. 2018 | Critically low | Y | PY | N | PY | Y | Y | PY | PY | N | N | Y | NA | NA | NA | N | N | NA | Y |
| Lafata et al. 2022 | Low | N | PY | N | PY | Y | Y | N | PY | NA | Y | Y | NA | NA | NA | Y | N | NA | Y |
| Lasla & Santoleri 2021 | Moderate | N | PY | N | PY | Y | Y | PY | PY | Y | Y | Y | NA | NA | NA | Y | Y | NA | Y |
| Lazzari et al. 2021 | Moderate | N | Y | N | Y | Y | Y | PY | Y | Y | Y | Y | NA | NA | NA | Y | Y | NA | Y |
| Lee 2018 | Critically low | N | PY | N | PY | N | N | PY | PY | N | N | Y | NA | NA | NA | N | N | NA | Y |
| Lin et al. 2017 | Critically low | N | PY | N | Y | Y | Y | PY | PY | N | N | Y | NA | NA | NA | N | N | NA | Y |
| Luo et al. 2022 | Low | Y | Y | N | Y | Y | Y | PY | Y | Y | NA | Y | Y | NA | Y | N | Y | Y | Y |
| Ma et al. 2020 | Moderate | Y | Y | Y | PY | Y | Y | PY | Y | Y | NA | N | NA | NA | NA | Y | Y | NA | N |
| Mathes et al. 2014 | Moderate | N | Y | Y | Y | Y | Y | PY | Y | Y | Y | Y | NA | NA | NA | Y | Y | NA | Y |
| Mausbach et al. 2015 | Low | N | PY | N | PY | N | N | PY | PY | Y | Y | N | Y | Y | Y | N | Y | Y | Y |
| Mc Grath et al. 2022 | Critically low | N | PY | N | Y | Y | Y | PY | Y | No | No | Y | NA | NA | NA | Y | Y | NA | Y |
| McGrady & Pai 2019 | Critically low | N | PY | N | PY | N | N | PY | Y | NA | No | Y | NA | NA | NA | N | N | NA | Y |
| McHugh et al. 2024 | Moderate | Y | Y | Y | PY | Y | N | PY | Y | Y | NA | N | NA | NA | NA | Y | Y | NA | Y |
| Molenaar et al. 2023 | Moderate | Y | Y | N | PY | Y | Y | PY | Y | Y | NA | Y | Y | NA | Y | Y | Y | Y | Y |
| Moon et al. 2017 | Moderate | N | PY | N | PY | Y | N | PY | Y | NA | Y | Y | NA | NA | NA | Y | Y | NA | Y |
| Mullan et al. 2023 | Moderate | Y | Y | Y | Y | Y | Y | PY | Y | PY | PY | N | NA | NA | NA | Y | Y | NA | Y |
| Murphy et al. 2012 | Low | N | PY | N | Y | Y | Y | PY | Y | NA | No | Y | NA | NA | NA | Y | Y | NA | Y |
| Naser et al. 2022 | Critically low | N | PY | Y | PY | Y | Y | N | Y | NA | PY | N | NA | Y | N | N | Y | N | Y |
| Noens et al. 2014 | Moderate | N | PY | N | Y | N | N | PY | PY | NA | Y | Y | NA | NA | NA | Y | Y | NA | Y |
| Oldenmenger et al. 2018 | Moderate | N | PY | Y | Y | Y | Y | PY | Y | Y | NA | Y | NA | NA | NA | Y | Y | NA | Y |
| Onvani et al. 2016 | Low | N | Y | N | PY | N | N | PY | Y | NA | Y | Y | NA | Y | N | N | Y | Y | Y |
| Ormel et al. 2017 | Critically low | N | Y | N | PY | Y | Y | PY | Y | No | NA | Y | NA | NA | NA | N | N | NA | Y |
| Parjanjpe et al. 2019 | Critically low | N | PY | N | PY | N | N | PY | Y | NA | No | Y | NA | NA | NA | N | N | NA | Y |
| Pezzolato et al. 2023 | Critically low | Y | Y | N | PY | Y | Y | N | Y | PY | PY | N | NA | NA | NA | N | Y | NA | Y |
| Puspitasari et al. 2020 | Critically low | N | PY | N | PY | Y | Y | PY | PY | NA | No | Y | NA | NA | NA | N | N | NA | Y |
| Puspitasari et al. 2021 | Critically low | N | PY | N | PY | Y | Y | PY | PY | N | N | Y | NA | NA | NA | N | N | NA | N |
| Puspitasari et al. 2022 | Low | Y | PY | N | PY | Y | Y | N | PY | Y | NA | N | NA | NA | NA | Y | Y | NA | N |
| Puts et al. 2014 | Critically low | N | PY | N | PY | Y | Y | PY | PY | NA | No | Y | NA | NA | NA | N | N | NA | Y |
| Ramos et al. 2024 | Low | Y | PY | Y | PY | Y | Y | N | Y | PY | NA | N | NA | NA | NA | Y | Y | NA | Y |
| Schwedhelm et al. 2016 | Moderate | Y | Y | Y | PY | Y | Y | PY | Y | NA | Y | Y | NA | Y | Y | Y | Y | Y | Y |
| Setyowibowo et al. 2022 | Moderate | N | Y | N | PY | Y | Y | PY | Y | Y | NA | Y | Y | NA | Y | Y | Y | Y | Y |
| Skrabal et al. 2021 | Critically low | N | PY | N | Y | N | Y | PY | Y | NA | No | Y | NA | NA | NA | N | N | NA | Y |
| Sotirova et al. 2021 | Low | N | PY | N | Y | Y | Y | PY | Y | N | N | Y | Y | Y | N | N | N | N | Y |
| Spei et al. 2023 | Moderate | N | Y | N | PY | Y | N | Y | Y | NA | Y | N | NA | Y | N | Y | Y | Y | Y |
| Spence et al. 2010 | Critically low | Y | PY | N | PY | N | N | Y | Y | N | N | Y | NA | NA | NA | N | N | NA | Y |
| Stalsberg & Darvik 2024 | Low | Y | PY | Y | PY | Y | N | PY | Y | No | NA | N | NA | NA | NA | Y | Y | NA | Y |
| Sun et al. 2024 | Moderate | Y | Y | N | PY | Y | Y | PY | Y | Y | NA | N | Y | NA | N | Y | Y | N | Y |
| Tan et al. 2021 | Moderate | Y | PY | N | PY | Y | Y | PY | Y | Y | NA | Y | NA | NA | NA | Y | N | NA | Y |
| Trauchburg et al. 2023 | Moderate | Y | Y | N | PY | Y | N | PY | Y | NA | Y | N | NA | Y | N | Y | N | Y | Y |
| van Gestel et al. 2022 | Low | N | Y | N | PY | Y | Y | PY | Y | Y | NA | Y | Y | NA | Y | Y | Y | N | Y |
| van Liew et al. 2014 | Critically low | N | PY | N | PY | N | N | PY | Y | NA | No | Y | NA | NA | NA | N | N | NA | Y |
| Verbrugghe et al. 2013 | Moderate | N | Y | N | PY | Y | Y | PY | Y | NA | Y | Y | NA | NA | NA | Y | N | NA | Y |
| Virani et al. 2024 | Low | Y | PY | N | PY | N | Y | N | Y | Y | Y | Y | NA | NA | NA | Y | Y | NA | Y |
| Visintini et al. 2023 | Moderate | Y | PY | N | Y | Y | Y | PY | Y | PY | PY | Y | NA | NA | NA | Y | Y | NA | Y |
| Wang et al. 2023 | Low | Y | Y | N | PY | Y | Y | PY | Y | Y | No | N | NA | NA | NA | Y | Y | NA | Y |
| Waseem et al. 2022 | Low | Y | PY | N | PY | Y | Y | Y | Y | PY | Y | Y | Y | N | Y | Y | Y | N | N |
| Watts et al. 2024 | Moderate | Y | Y | N | Y | Y | Y | PY | PY | Y | Y | N | NA | NA | NA | Y | N | NA | Y |
| Yang et al. 2023 | Critically low | Y | PY | N | PY | Y | N | PY | Y | No | No | N | NA | NA | NA | N | Y | NA | Y |
| Yussof et al. 2022 | Moderate | N | Y | N | PY | Y | Y | PY | Y | NA | Y | Y | NA | NA | NA | Y | Y | NA | Y |
| Zerillo et al. 2018 | Critically low | N | PY | N | PY | Y | Y | PY | Y | NA | No | Y | NA | NA | NA | N | N | NA | Y |
| Zhong et al. 2020 | Moderate | N | PY | N | PY | Y | Y | PY | Y | NA | Y | Y | NA | Y | N | Y | Y | Y | Y |
| Zou et al. 2023 | Low | Y | PY | N | PY | Y | Y | PY | PY | Y | PY | N | NA | NA | NA | Y | Y | NA | Y |
| Zullig et al. 2023 | Low | N | PY | N | PY | Y | N | N | PY | NA | PY | N | NA | NA | NA | Y | Y | NA | Y |

Abbreviations: Y = Yes, PY = Partial Yes, N = No
