# Supplementary material for: Patient-reported outcome measures (PROMs) capturing therapy adherence of cancer patients: an umbrella review
Source: Patient Relat Outcome Meas. 2026 May 12;17(1):25–34. doi: 10.1080/1179271X.2026.2669130 (PMC13536962; doi:10.1080/1179271X.2026.2669130)
Supplement: Supplementary file 3 [file DPRO_A_2669130_SM6248.docx]

**Table S4 Patient Reported Outcome Measures (PROMs) used in included primary studies**

| SLR Author(s), Year | Study Author(s), Year | Patient Reported Outcome Measure (PROM) | Short Form | Validation status | Adherence Dimension: Medication | Adherence Dimension: Diet | Adherence Dimension: Exercise | Adherence Dimension: Lifestyle | Adherence Dimension: Other | Other specific |
| --- | --- | --- | --- | --- | --- | --- | --- | --- | --- | --- |
| Abboud et al. 2021 [1] | Augustus et al. 2021 | Diary | NR | NR | No | Yes | No | No | No | N/A |
| Abboud et al. 2021 | Martin-McGill et al. 2020 | Diary | NR | NR | No | Yes | No | No | No | N/A |
| Agirre-Elordui et al. 2024 [2] | Courneya et al. 2016 | Total Physical Activity Questionnaire | TPAQ | Validated | No | No | Yes | No | No | N/A |
| Agirre-Elordui et al. 2024 | Hawkes et al. 2013 | Cancer Council Victoria Food Frequency Questionnaire | FFQ | Validated | No | Yes | No | No | No | N/A |
| Agirre-Elordui et al. 2024 | Hawkes et al. 2013 | Godin Leisure-Time Physical Activity Questionnaire | GLTPAQ | Adapted | No | No | Yes | No | No | N/A |
| Agirre-Elordui et al. 2024 | Mayer et al. 2018 | Godin Leisure-Time Physical Activity Questionnaire | GLTPAQ | Validated | No | No | Yes | No | No | N/A |
| fAgirre-Elordui et al. 2024 | Pinto et al. 2013 | 7-Day Physical Activity Recall | 7D-PAR | Validated | No | No | Yes | No | No | N/A |
| Ahmed & Loiselle 2023 [3] | Bellomo et al. 2017 | Adherence Starts with Knowledge-12 | ASK-12 | Validated | Yes | No | No | No | No | N/A |
| Ahmed & Loiselle 2023 | Bordonaro et al. 2012 | Questionnaire | NR | NR | Yes | No | No | No | No | N/A |
| Ahmed & Loiselle 2023 | Bordonaro et al. 2014 | Questionnaire | NR | Not Validated | Yes | No | No | No | No | N/A |
| Ahmed & Loiselle 2023 | Boucher et al. 2015 | Morisky Medication Adherence Scale-8 | MMAS-8 | Validated | Yes | No | No | No | No | N/A |
| Ahmed & Loiselle 2023 | Bouleftour et al. 2021 | Morisky Medication Adherence Scale-8 | MMAS-8 | Validated | Yes | No | No | No | No | N/A |
| Ahmed & Loiselle 2023 | Dennis et al. 2020 | Questionnaire | NR | Validated | Yes | No | No | No | No | N/A |
| Ahmed & Loiselle 2023 | El-Behadli et al. 2022 | Questionnaire | NR | NR | Yes | No | No | No | No | N/A |
| Ahmed & Loiselle 2023 | Fischer et al. 2018 | Morisky Medication Adherence Scale-8 | MMAS-8 | Validated | Yes | No | No | No | No | N/A |
| Ahmed & Loiselle 2023 | Gebbia et al. 2013 | Basel Assessment of Adherence Scale | BAAS | Validated | Yes | No | No | No | No | N/A |
| Ahmed & Loiselle 2023 | Gebbia et al. 2013 | Visual Analog Scale | VAS | NR | Yes | No | No | No | No | N/A |
| Ahmed & Loiselle 2023 | Ghiggia et al. 2021 | Questionnaire | NR | NR | Yes | No | No | No | No | N/A |
| Ahmed & Loiselle 2023 | Gönderen Çakmak & Kapucu 2021 | Medication Adherence Self-Efficacy Scale | MASES | Validated | Yes | No | No | No | No | N/A |
| Ahmed & Loiselle 2023 | Gönderen Çakmak & Kapucu 2021 | Oral Chemotherapy Adherence Scale | OCAS | Validated | Yes | No | No | No | No | N/A |
| Ahmed & Loiselle 2023 | Jean et al. 2016 | Morisky Medication Adherence Scale-4 | MMAS-4 | Validated | Yes | No | No | No | No | N/A |
| Ahmed & Loiselle 2023 | Karaaslan-Eser & Ayaz-Alkaya 2021 | Oral Chemotherapy Adherence Scale | OCAS | Validated | Yes | No | No | No | No | N/A |
| Ahmed & Loiselle 2023 | Kimura et al. 2017 | Questionnaire | NR | NR | Yes | No | No | No | No | N/A |
| Ahmed & Loiselle 2023 | Lau-Min et al. 2020 | Questionnaire | NR | NR | Yes | No | No | No | No | N/A |
| Ahmed & Loiselle 2023 | Lin et al. 2021 | Questionnaire | NR | NR | Yes | No | No | No | No | N/A |
| Ahmed & Loiselle 2023 | Newman et al. 2018 | Diary | NR | NR | Yes | No | No | No | No | N/A |
| Ahmed & Loiselle 2023 | Porcher et al. 2020 | Questionnaire | NR | NR | Yes | No | No | No | No | N/A |
| Ahmed & Loiselle 2023 | Ramesh et al. 2015 | Medication Adherence Rating Scale | MARS-1 | Validated | Yes | No | No | No | No | N/A |
| Ahmed & Loiselle 2023 | Ramesh et al. 2015 | Beliefs About Medicines Questionnaire | BMQ | Validated | Yes | No | No | No | No | N/A |
| Ahmed & Loiselle 2023 | Rodriguez et al. 2017 | Questionnaire | NR | NR | Yes | No | No | No | No | N/A |
| Ahmed & Loiselle 2023 | Schneider et al. 2014 | Questionnaire | NR | Not Validated | Yes | No | No | No | No | N/A |
| Ahmed & Loiselle 2023 | Signorelli et al. 2022 | Morisky Medication Adherence Scale-8 | MMAS-8 | Validated | Yes | No | No | No | No | N/A |
| Ahmed & Loiselle 2023 | Skrabal et al. 2022 | Medication Adherence Rating Scale-5 | MARS-5 | Validated | Yes | No | No | No | No | N/A |
| Ahmed & Loiselle 2023 | Sommers et al. 2012 | Morisky Medication Adherence Scale-8 | MMAS-8 | Validated | Yes | No | No | No | No | N/A |
| Ahmed & Loiselle 2023 | Sun et al. 2021 | Questionnaire | NR | NR | Yes | No | No | No | No | N/A |
| Ahmed & Loiselle 2023 | Tokdemir et al. 2017 | Medication Adherence Self-Efficacy Scale | MASES | Validated | Yes | No | No | No | No | N/A |
| Ahmed & Loiselle 2023 | Vella et al. 2017 | Morisky Medication Adherence Scale-8 | MMAS-8 | Validated | Yes | No | No | No | No | N/A |
| Ahmed & Loiselle 2023 | Wang et al. 2012 | Morisky Medication Adherence Scale-8 | MMAS-8 | Validated | Yes | No | No | No | No | N/A |
| Ahmed & Loiselle 2023 | Zhang et al. 2022 | Morisky Medication Adherence Scale-8 | C-MMAS-8 | Validated | Yes | No | No | No | No | N/A |
| Ahmed & Loiselle 2023 | Zukin et al. 2019 | Morisky Medication Adherence Scale-8 | MMAS-8 | Validated | Yes | No | No | No | No | N/A |
| Alves et al. 2016 [4] | Efficace et al. 2012 | Morisky Medication Adherence Scale | MMAS | Adapted | Yes | No | No | No | No | N/A |
| Alves et al. 2016 | Eliasson et al. 2011 | Single Item Measure | NR | NR | Yes | No | No | No | No | N/A |
| Alves et al. 2016 | Hirji et al. 2013 | Questionnaire | NR | Adapted | Yes | No | No | No | No | N/A |
| Alves et al. 2016 | Jönsson et al. 2012 | Morisky Medication Adherence Scale-9 | MMAS-9 | Validated | Yes | No | No | No | No | N/A |
| Alves et al. 2016 | Noens et al. 2009 | Basel Assessment of Adherence Scale | BAAS | Validated | Yes | No | No | No | No | N/A |
| Alves et al. 2016 | Noens et al. 2009 | Visual Analog Scale | VAS | NR | Yes | No | No | No | No | N/A |
| Alves et al. 2016 | Rosa et al. 2013 | Simplified Medication Adherence Questionnaire | SMAQ | Validated | Yes | No | No | No | No | N/A |
| Atkinson et al. 2016 [5] | Addeo et al. 2015 | Questionnaire | NR | NR | Yes | No | No | No | No | N/A |
| Atkinson et al. 2016 | Daouphars et al. 2013 | Questionnaire | NR | Validated | Yes | No | No | No | No | N/A |
| Atkinson et al. 2016 | Gebbia et al. 2013 | Basel Assessment of Adherence Scale | BAAS | Validated | Yes | No | No | No | No | N/A |
| Atkinson et al. 2016 | Gebbia et al. 2013 | Visual Analog Scale | VAS | NR | Yes | No | No | No | No | N/A |
| Atkinson et al. 2016 | Schneider et al. 2014 | Questionnaire | NR | NR | Yes | No | No | No | No | N/A |
| Atkinson et al. 2016 | Timmers et al. 2014 | Medication Adherence Rating Scale-5 | MARS-5 | Validated | Yes | No | No | No | No | N/A |
| Atkinson et al. 2016 | Timmers et al. 2015 | Medication Adherence Rating Scale-5 | MARS-5 | Validated | Yes | No | No | No | No | N/A |
| Atkinson et al. 2016 | Walter et al. 2013 | Questionnaire | NR | NR | Yes | No | No | No | No | N/A |
| Atkinson et al. 2016 | Waterhouse et al. 1993 | Questionnaire | NR | Validated | Yes | No | No | No | No | N/A |
| Barnes et al. 2019 [6] | Baguely et al. 2017 | Wollongong Dietary Inventory | NR | Validated | No | Yes | Yes | No | No | N/A |
| Barnes et al. 2019 | Bourke et al. 2014 | Diary | NR | NR | No | Yes | No | No | No | N/A |
| Barnes et al. 2019 | Bourke et al. 2014 | Godin Leisure-Time Physical Activity Questionnaire | GLTPAQ | Validated | No | No | Yes | No | No | N/A |
| Barnes et al. 2019 | Davison et al. 2011 | Dietary Health Questionnaire | DHQ | Adapted | Yes | Yes | Yes | No | No | N/A |
| Barnes et al. 2019 | Focht et al. 2018 | Dietary Health Questionnaire II | DHQ II | Validated | No | Yes | No | No | No | N/A |
| Barnes et al. 2019 | Gilbert et al. 2016 | Diary | NR | NR | No | Yes | No | No | No | N/A |
| Barnes et al. 2019 | Kiwata et al. 2017 | Diary | NR | NR | No | Yes | No | No | No | N/A |
| Barnes et al. 2019 | Nobes et al. 2011 | Diary | NR | NR | No | Yes | Yes | No | No | N/A |
| Barnes et al. 2019 | O'Neill et al. 2015 | Diary | NR | NR | Yes | Yes | Yes | No | No | N/A |
| Barnes et al. 2019 | Owen et al. 2017 | Single Item Measure | NR | Validated | No | Yes | Yes | Yes | No | N/A |
| Barnes et al. 2019 | Richard et al. 2015 | Diary | NR | NR | No | Yes | No | No | No | N/A |
| Baydoun et al. 2021 [7] | Campbell et al. 2012 | Diary | NR | NR | No | No | No | No | Yes | Meditation |
| Baydoun et al. 2021 | Carlson et al. 2007 | Diary | NR | NR | No | No | Yes | No | No | N/A |
| Baydoun et al. 2021 | Compen et al. 2018 | Diary | NR | NR | No | No | No | No | Yes | Meditation |
| Baydoun et al. 2021 | Foley et al. 2010 | Diary | NR | NR | No | No | Yes | No | Yes | Meditation |
| Baydoun et al. 2021 | Johannsen et al. 2016 | Diary | NR | NR | No | No | Yes | No | No | N/A |
| Baydoun et al. 2021 | Johns et al. 2015 | Diary | NR | NR | No | No | Yes | No | No | N/A |
| Baydoun et al. 2021 | Johns et al. 2016 | Diary | NR | NR | No | No | Yes | No | No | N/A |
| Baydoun et al. 2021 | Labelle et al. 2015 | Diary | NR | NR | No | No | Yes | No | No | N/A |
| Baydoun et al. 2021 | Lengacher et al. 2009 | Diary | NR | NR | No | No | Yes | No | No | N/A |
| Baydoun et al. 2021 | Lengacher et al. 2012 | Diary | NR | NR | No | No | Yes | No | No | N/A |
| Baydoun et al. 2021 | Lengacher et al. 2018 | Diary | NR | NR | No | No | Yes | No | No | N/A |
| Baydoun et al. 2021 | Poletti et al. 2019 | Diary | NR | NR | No | No | Yes | No | No | N/A |
| Baydoun et al. 2021 | Russell et al. 2019 | Diary | NR | NR | No | No | Yes | No | No | N/A |
| Baydoun et al. 2021 | Speca et al. 2000 | Diary | NR | NR | No | No | Yes | No | No | N/A |
| Baydoun et al. 2021 | Tamagawa et al. 2015 | Diary | NR | NR | No | No | Yes | No | No | N/A |
| Baydoun et al. 2021 | Zernicke et al. 2014 | Diary | NR | NR | No | No | Yes | No | Yes | Meditation |
| Baydoun et al. 2021 | Zhao et al. 2020 | Diary | NR | NR | No | No | No | No | Yes | Meditation |
| Bright et al. 2023 [8] | Albert et al. 2011 | Questionnaire | NR | NR | Yes | No | No | No | No | N/A |
| Bright et al. 2023 | Alkhayyat et al. 2012 | Questionnaire | NR | Not Validated | Yes | No | No | No | No | N/A |
| Bright et al. 2023 | Arch et al. 2022 | Single Item Measure | NR | Validated | Yes | No | No | No | No | N/A |
| Bright et al. 2023 | Getachew et al. 2022 | Simplified Medication Adherence Questionnaire | SMAQ | Validated | Yes | No | No | No | No | N/A |
| Bright et al. 2023 | Graetz et al. 2018 | Medication Adherence Reasons | MARS-1 | Validated | Yes | No | No | No | No | N/A |
| Bright et al. 2023 | Graetz et al. 2018 | Morisky Medication Adherence Scale-4 | MMAS-4 | Validated | Yes | No | No | No | No | N/A |
| Bright et al. 2023 | Hadji et al. 2013 | Morisky Medication Adherence Scale-4 | MMAS-4 | Adapted | Yes | No | No | No | No | N/A |
| Bright et al. 2023 | Irwin et al. 2015 | Diary | NR | NR | Yes | No | Yes | No | No | N/A |
| Bright et al. 2023 | Jacobs et al. 2022 | Medication Adherence Rating Scale-5 | MARS-5 | Validated | Yes | No | No | No | No | N/A |
| Bright et al. 2023 | Krok-Schoen et al. 2019 | Morisky Medication Adherence Scale | MMAS | Adapted | Yes | No | No | No | No | N/A |
| Bright et al. 2023 | Markopoulos et al. 2015 | Single Item Measure | NR | NR | Yes | No | No | No | No | N/A |
| Bright et al. 2023 | Moon et al. 2019b | Medication Adherence Rating Scale-5 | MARS-5 | Validated | Yes | No | No | No | No | N/A |
| Bright et al. 2023 | Mougalian et al. 2017 | Single Item Measure | NR | NR | Yes | No | No | No | No | N/A |
| Bright et al. 2023 | Neven et al. 2014 | Single Item Measure | NR | NR | Yes | No | No | No | No | N/A |
| Bright et al. 2023 | Park et al. 2022a | Diary | NR | NR | Yes | No | No | No | No | N/A |
| Bright et al. 2023 | Ream et al. 2021 | Endocrine Therapy Medication Usage Questionnaire | ETMUQ | Validated | Yes | No | No | No | No | N/A |
| Bright et al. 2023 | Tan et al. 2020 | Simplified Medication Adherence Questionnaire | SMAQ | Validated | Yes | No | No | No | No | N/A |
| Bright et al. 2023 | Ziller et al. 2013 | Questionnaire | NR | Not Validated | Yes | No | No | No | No | N/A |
| Bruns et al. 2016 [9] | Dronkers et al. 2010 | Diary | NR | NR | No | No | Yes | No | No | N/A |
| Bruns et al. 2016 | Gillis et al. 2014 | Diary | NR | NR | No | Yes | No | No | No | N/A |
| Bruns et al. 2016 | Gillis et al. 2014 | Diary | NR | NR | No | No | Yes | No | No | N/A |
| Bruns et al. 2016 | Li et al. 2012 | Diary | NR | NR | No | No | Yes | No | No | N/A |
| Burke et al. 2024 [10] | Brown et al. 2020 | Diary | NR | NR | Yes | No | No | No | No | N/A |
| Burke et al. 2024 | Wooten et al. 2021 | Diary | NR | NR | No | No | Yes | No | No | N/A |
| Cahir et al. 2015 [11] | Atkins & Fallowfield 2006 | Questionnaire | NR | NR | Yes | No | No | No | No | N/A |
| Cahir et al. 2015 | Font et al. 2012 | Single Item Measure | NR | NR | Yes | No | No | No | No | N/A |
| Cahir et al. 2015 | Liu et al. 2013 | Questionnaire | NR | NR | Yes | No | No | No | No | N/A |
| Cahir et al. 2015 | Markkula et al. 2012 | Questionnaire | NR | NR | Yes | No | No | No | No | N/A |
| Cahir et al. 2015 | Simon et al. 2014 | Questionnaire | NR | NR | Yes | No | No | No | No | N/A |
| Cahir et al. 2015 | Ziller et al. 2009 | Questionnaire | NR | Validated | Yes | No | No | No | No | N/A |
| Carr & Rosengarten 2021 [12] | Kondryn et al. 2009 | Cancer Patient Self-Report Non-Adherence Questionnaire | NR | Not Validated | Yes | No | No | No | No | N/A |
| Charters et al. 2024 [13] | Cnossen et al. 2014 | Diary | NR | NR | No | No | Yes | No | No | N/A |
| Chen et al. 2022 [14] | Alegria et al. 2016 | Food Frequencies Questionnaire | FFQ | Validated | No | Yes | No | No | No | N/A |
| Chen et al. 2022 | Alegria et al. 2016 | Healthy Eating Index for Spanish Diet | HEISD | Validated | No | Yes | No | No | No | N/A |
| Chen et al. 2022 | Alegria et al. 2016 | MedDietScore | MDS | Validated | No | Yes | No | No | No | N/A |
| Chen et al. 2022 | Blair et al. 2019 | International Physical Activity Questionnaire | IPAQ | Validated | No | No | Yes | No | No | N/A |
| Chen et al. 2022 | Bourke et al. 2011 | Diary | NR | NR | No | No | Yes | No | No | N/A |
| Chen et al. 2022 | Hawkes et al. 2013 | Food Frequencies Questionnaire | FFQ | Validated | No | Yes | No | No | No | N/A |
| Chen et al. 2022 | Hawkes et al. 2013 | Godin Leisure-Time Physical Activity Questionnaire | GLTPAQ | Adapted | No | No | Yes | No | No | N/A |
| Chen et al. 2022 | Hawkins et al. 2015 | Questionnaire | NR | NR | No | Yes | No | No | No | N/A |
| Chen et al. 2022 | James et al. 2016 | 7-Day Physical Activity Recall | 7D-PAR | Validated | No | No | Yes | No | No | N/A |
| Chen et al. 2022 | James et al. 2016 | Food Frequencies Questionnaire | FFQ | NR | No | Yes | No | No | No | N/A |
| Chen et al. 2022 | Ko et al. 2010 | Questionnaire | NR | NR | No | Yes | No | No | No | N/A |
| Chen et al. 2022 | Ko et al. 2010 | Block Food Frequencies Questionnaire | Block FFQ | Adapted | No | Yes | No | No | No | N/A |
| Chen et al. 2022 | Lee et al. 2018 | Diary | NR | NR | No | Yes | No | No | No | N/A |
| Chen et al. 2022 | Lewis et al. 2016 | DIQN | DIQN | NR | No | Yes | No | No | No | N/A |
| Chen et al. 2022 | Lewis et al. 2016 | Diet History Questionnaire | DHQ | Validated | No | Yes | No | No | No | N/A |
| Chen et al. 2022 | Lewis et al. 2016 | Quick Food Scan Questionnaire | QFSQ | Adapted | No | Yes | No | No | No | N/A |
| Chen et al. 2022 | Satia et al. 2004 | Block 2005 Food Frequency Questionnaire | Block 2005 FFQ, modified | Adapted | No | Yes | No | No | No | N/A |
| Chen et al. 2022 | Satia et al. 2004 | 7-Day Physical Activity Recall | 7D-PAR | Validated | No | No | Yes | No | No | N/A |
| Chen et al. 2023 [15] | Alvarez-Bustos et al. 2021 | Mediterranean Diet Adherence Score | MEDAS | Validated | No | Yes | No | No | No | N/A |
| Chen et al. 2023 | Barchitta et al. 2020 | Mediterranean Diet Adherence Score | MEDAS | Validated | No | Yes | No | No | No | N/A |
| Chen et al. 2023 | Di Maso et al. 2020 | Food Frequencies Questionnaire | FFQ | Validated | No | Yes | No | No | No | N/A |
| Chen et al. 2023 | Di Maso et al. 2020 | Mediterranean Diet Score | MDS | Validated | No | Yes | No | No | No | N/A |
| Chen et al. 2023 | Ergas et al. 2021 | Healthy Eating Index-2015 | HEI-2015 | Validated | No | Yes | No | No | No | N/A |
| Chen et al. 2023 | Ergas et al. 2021 | Block 2005 Food Frequency Questionnaire | Block 2005 FFQ | Validated | No | Yes | No | No | No | N/A |
| Chen et al. 2023 | Ergas et al. 2021 | Alternate Mediterranean Diet score | aMED | Validated | No | Yes | No | No | No | N/A |
| Chen et al. 2023 | Ergas et al. 2021 | American Cancer Society Score | ACS Score | Validated | No | Yes | No | No | No | N/A |
| Chen et al. 2023 | Ergas et al. 2021 | Dietary Approaches to Stop Hypertension | DASH | Validated | No | Yes | No | No | No | N/A |
| Chen et al. 2023 | Karavasiloglou et al. 2019 | Healthy Eating Index 2005 | HEI-2005 | Validated | No | Yes | No | No | No | N/A |
| Chen et al. 2023 | Karavasiloglou et al. 2019 | Mediterranean Diet Score | MDS | Validated | No | Yes | No | No | No | N/A |
| Chen et al. 2023 | Kim et al. 2011 | Alternate Healthy Eating Index | AHEI | Validated | No | Yes | No | No | No | N/A |
| Chen et al. 2023 | Kim et al. 2011 | Alternate Mediterranean Diet score | aMED | Validated | No | Yes | No | No | No | N/A |
| Chen et al. 2023 | Kim et al. 2011 | Diet Quality Index-Revised | DQIR | Validated | No | Yes | No | No | No | N/A |
| Chen et al. 2023 | Kim et al. 2011 | Food Frequencies Questionnaire | FFQ | Validated | No | Yes | No | No | No | N/A |
| Chen et al. 2023 | Kim et al. 2011 | Recommended Food Score | RFS | Validated | No | Yes | No | No | No | N/A |
| Chen et al. 2023 | Lorenzo et al. 2020 | Mediterranean Diet Adherence Score | MEDAS | Adapted | No | Yes | No | No | No | N/A |
| Chen et al. 2023 | Lorenzo et al. 2020 | Food Frequencies Questionnaire | FFQ | Validated | No | Yes | No | No | No | N/A |
| Chen et al. 2023 | Negrati et al. 2021 | Mediterranean Diet Score | MDS | Validated | No | Yes | No | No | No | N/A |
| Chen et al. 2023 | Parma et al. 2022 | Mediterranean Diet Adherence Score | MEDAS | Validated | No | Yes | No | No | No | N/A |
| Chen et al. 2023 | Porciello et al. 2019 | Mediterranean Diet Adherence Score | MEDAS | Validated | No | Yes | No | No | No | N/A |
| Chen et al. 2023 | Porciello et al. 2019 | Mediterranean Diet Adherence Score | MEDAS | Validated | No | Yes | No | No | No | N/A |
| Chen et al. 2023 | Ramirez et al. 2017 | Food Frequencies Questionnaire | FFQ | NR | No | Yes | No | No | No | N/A |
| Chen et al. 2023 | Ruiz-Casado et al. 2020 | Mediterranean Diet Adherence Score | MEDAS | Validated | No | Yes | No | No | No | N/A |
| Chen et al. 2023 | Skouroliakou et al. 2017 | Diary | NR | NR | No | Yes | No | No | No | N/A |
| Chen et al. 2023 | Skouroliakou et al. 2017 | Food Frequencies Questionnaire | FFQ | Validated | No | Yes | No | No | No | N/A |
| Chen et al. 2023 | Zuniga et al. 2019 | Spice And Herbs Questionnaire | NR | Validated | No | Yes | No | No | No | N/A |
| Chen et al. 2023 | Zuniga et al. 2019 | Questionnaire | NR | Validated | No | Yes | No | No | No | N/A |
| Cheung et al. 2023 [16] | Capozzi et al. 2016 | Godin Leisure-Time Physical Activity Questionnaire | GLTPAQ | Validated | No | No | Yes | No | No | N/A |
| Cheung et al. 2023 | Hall et al. 2021 | Diary | NR | NR | No | No | Yes | No | No | N/A |
| Cheung et al. 2023 | Naito et al. 2019 | Diary | NR | NR | No | Yes | Yes | No | No | N/A |
| Cheung et al. 2023 | Storck et al. 2020 | Diary | NR | NR | No | Yes | No | No | No | N/A |
| Cheung et al. 2023 | Uster et al. 2018 | Diary | NR | NR | No | Yes | No | No | No | N/A |
| Chlebowski & Geller 2006 [17] | Grunfeld et al. 2005 | Beliefs About Medicines Questionnaire | BMQ | Validated | Yes | No | No | No | No | N/A |
| Chlebowski & Geller 2006 | Grunfeld et al. 2005 | Medication Adherence Rating Scale-5 | MARS-5 | Validated | Yes | No | No | No | No | N/A |
| Chlebowski & Geller 2006 | Grunfeld et al. 2005 | Single Item Measure | NR | NR | Yes | No | No | No | No | N/A |
| Chlebowski & Geller 2006 | Lash et al. 2006 | Questionnaire | NR | NR | Yes | No | No | No | No | N/A |
| Chlebowski & Geller 2006 | Waterhouse et al. 1993 | Questionnaire | NR | Validated | Yes | No | No | No | No | N/A |
| Chow & Tan 2024 [18] | Graetz et al. 2018 | Medication Adherence Reasons | MARS-1 | Validated | Yes | No | No | No | No | N/A |
| Chow & Tan 2024 | Graetz et al. 2018 | Morisky Medication Adherence Scale-4 | MMAS-4 | Validated | Yes | No | No | No | No | N/A |
| Chow & Tan 2024 | Greer et al. 2020 | Morisky Medication Adherence Scale-4 | MMAS-4 | Validated | Yes | No | No | No | No | N/A |
| Chow & Tan 2024 | Karaaslan-Eser & Ayaz-Alkaya 2021 | Oral Chemotherapy Adherence Scale | OCAS | Validated | Yes | No | No | No | No | N/A |
| Chow & Tan 2024 | Mir et al. 2022 | Morisky Medication Adherence Scale-4 | MMAS-4 | Validated | Yes | No | No | No | No | N/A |
| Claros et al. 2019 [19] | Amorim et al. 2015 | Adherence Determinants Questionnaire | ADQ | Validated | Yes | No | No | No | No | N/A |
| Claros et al. 2019 | Bagcivan et al. 2015 | Oral Chemotherapy Adherence Scale | OCAS | Validated | Yes | No | No | No | No | N/A |
| Claros et al. 2019 | Daouphars et al. 2013 | Questionnaire | NR | Validated | Yes | No | No | No | No | N/A |
| Claros et al. 2019 | Jacobsen et al. 2008 | Medication Adherence Rating Scale-4 | DMARS-4 | Validated | Yes | No | No | No | No | N/A |
| Deng et al. 2024 [20] | Fung et al. 2015 | Questionnaire | NR | Not Validated | No | No | Yes | No | Yes | Respiratory Rehabilitation |
| Eliasson et al. 2012 [21] | Daouphars et al. 2011 | Questionnaire | NR | Validated | Yes | No | No | No | No | N/A |
| Eliasson et al. 2012 | Noens et al. 2009 | Visual Analog Scale | VAS | NR | No | No | No | No | Yes | Scheduled Appointments |
| Eliasson et al. 2012 | Noens et al. 2009 | Basel Assessment of Adherence Scale | BAAS | Validated | Yes | No | No | No | No | N/A |
| Finitsis et al. 2018 [22] | Graetz et al. 2018 | Medication Adherence Reasons | MARS-1 | Validated | Yes | No | No | No | No | N/A |
| Finitsis et al. 2018 | Graetz et al. 2018 | Morisky Medication Adherence Scale-4 | MMAS-4 | Validated | Yes | No | No | No | No | N/A |
| Finitsis et al. 2018 | Hadji et al. 2013 | Morisky Medication Adherence Scale-4 | MMAS-4 | Adapted | Yes | No | No | No | No | N/A |
| Finitsis et al. 2018 | Heisig et al. 2015 | Single Item Measure | NR | Validated | Yes | No | No | No | No | N/A |
| Finitsis et al. 2018 | Markopoulos et al. 2015 | Single Item Measure | NR | NR | Yes | No | No | No | No | N/A |
| Finitsis et al. 2018 | Ziller et al. 2013 | Questionnaire | NR | Not Validated | Yes | No | No | No | No | N/A |
| Fleming et al. 2020 [23] | Bowles et al. 2012 | Morisky Medication Adherence Scale-4 | MMAS-4 | Validated | Yes | No | No | No | No | N/A |
| Fleming et al. 2020 | Brett et al. 2018 | Medication Adherence Rating Scale-5 | MARS-5 | Validated | Yes | No | No | No | No | N/A |
| Fleming et al. 2020 | Brier et al. 2015 | Single Item Measure | NR | NR | Yes | No | No | No | No | N/A |
| Fleming et al. 2020 | Brier et al. 2015 | Visual Analog Scale | VAS | NR | Yes | No | No | No | No | N/A |
| Fleming et al. 2020 | Brier et al. 2015 | Morisky Medication Adherence Scale-8 | MMAS-8 | Adapted | Yes | No | No | No | No | N/A |
| Fleming et al. 2020 | Bright et al. 2016 | Morisky Medication Adherence Scale-8 | MMAS-8 | Adapted | Yes | No | No | No | No | N/A |
| Fleming et al. 2020 | Corter et al. 2018 | Beliefs About Medicines Questionnaire | BMQ | Adapted | Yes | No | No | No | No | N/A |
| Fleming et al. 2020 | Grossman 2016 | Single Item Measure | NR | NR | Yes | No | No | No | No | N/A |
| Fleming et al. 2020 | Hadji et al. 2014 | Questionnaire | NR | NR | Yes | No | No | No | No | N/A |
| Fleming et al. 2020 | Henry et al. 2017 | Medication Adherence Rating Scale-5 | MARS-5 | Validated | Yes | No | No | No | No | N/A |
| Fleming et al. 2020 | Iacorossi et al. 2016 | Morisky Medication Adherence Scale-8 | MMAS-8 | Validated | Yes | No | No | No | No | N/A |
| Fleming et al. 2020 | Jackisch et al. 2019 | Questionnaire | NR | NR | Yes | No | No | No | No | N/A |
| Fleming et al. 2020 | Kimmick et al. 2015 | Morisky Medication Adherence Scale-8 | MMAS-8 | Validated | Yes | No | No | No | No | N/A |
| Fleming et al. 2020 | Kimmick et al. 2015 | Questionnaire | NR | NR | Yes | No | No | No | No | N/A |
| Fleming et al. 2020 | Kool et al. 2014 | Questionnaire | NR | NR | Yes | No | No | No | No | N/A |
| Fleming et al. 2020 | Kyvernitakis et al. 2014 | Questionnaire | NR | NR | Yes | No | No | No | No | N/A |
| Fleming et al. 2020 | Liu et al. 2013 | Questionnaire | NR | NR | Yes | No | No | No | No | N/A |
| Fleming et al. 2020 | Markovitz et al. 2017 | Morisky Medication Adherence Scale-4 | MMAS-4 | Validated | Yes | No | No | No | No | N/A |
| Fleming et al. 2020 | Moon et al. 2019a | Medication Adherence Rating Scale-5 | MARS-5 | Validated | Yes | No | No | No | No | N/A |
| Fleming et al. 2020 | Nestoriuc et al. 2017 | Questionnaire | NR | Validated | Yes | No | No | No | No | N/A |
| Fleming et al. 2020 | Pan et al. 2018 | Single Item Measure | NR | Validated | Yes | No | No | No | No | N/A |
| Fleming et al. 2020 | Pinheiro et al. 2017 | Questionnaire | NR | NR | Yes | No | No | No | No | N/A |
| Fleming et al. 2020 | Pinheiro et al. 2017 | Morisky Medication Adherence Scale | MMAS | Adapted | Yes | No | No | No | No | N/A |
| Fleming et al. 2020 | Quinn et al. 2016 | Morisky Medication Adherence Scale-8 | MMAS-8 | Validated | Yes | No | No | No | No | N/A |
| Fleming et al. 2020 | Schover et al. 2014 | 3-item Adherence Estimator | NR | Validated | Yes | No | No | No | No | N/A |
| Fleming et al. 2020 | Shinn et al. 2019 | Questionnaire | NR | Not Validated | Yes | No | No | No | No | N/A |
| Fleming et al. 2020 | Spencer et al. 2020 | Questionnaire | NR | NR | Yes | No | No | No | No | N/A |
| Fleming et al. 2020 | Stahlschmidt et al. 2019 | Morisky Medication Adherence Scale-4 | MMAS-4 | Validated | Yes | No | No | No | No | N/A |
| Fleming et al. 2020 | Stahlschmidt et al. 2020 | Morisky Medication Adherence Scale-4 | MMAS-4 | Validated | Yes | No | No | No | No | N/A |
| Fleming et al. 2020 | Stanton et al. 2014 | Morisky Medication Adherence Scale-4 | MMAS-4 | Adapted | Yes | No | No | No | No | N/A |
| Fleming et al. 2020 | Walker et al. 2016 | Morisky Medication Adherence Scale | MMAS | Adapted | Yes | No | No | No | No | N/A |
| Fleming et al. 2020 | Wouters et al. 2014 | Medication Adherence Rating Scale-5 | MARS-5 | Validated | Yes | No | No | No | No | N/A |
| Fleming et al. 2020 | Wouters et al. 2014 | Morisky Medication Adherence Scale-8 | MMAS-8 | Validated | Yes | No | No | No | No | N/A |
| Fleming et al. 2020 | Wouters et al. 2014 | Questionnaire | NR | NR | Yes | No | No | No | No | N/A |
| Fleming et al. 2020 | Xu et al. 2020b | Morisky Medication Adherence Scale-4 | MMAS-4 | Validated | Yes | No | No | No | No | N/A |
| Fleming et al. 2020 | Ziller et al. 2009 | Questionnaire | NR | Validated | Yes | No | No | No | No | N/A |
| Ganna et al. 2024 [24] | Arch et al. 2022 | Single Item Measure | NR | Validated | No | No | No | No | No | N/A |
| Ganna et al. 2024 | Getachew et al. 2022 | Simplified Medication Adherence Questionnaire | SMAQ | Validated | Yes | No | No | No | No | N/A |
| Ganna et al. 2024 | Graetz et al. 2018 | Medication Adherence Reasons | MARS-1 | Validated | Yes | No | No | No | No | N/A |
| Ganna et al. 2024 | Graetz et al. 2018 | Morisky Medication Adherence Scale-4 | MMAS-4 | Validated | Yes | No | No | No | No | N/A |
| Ganna et al. 2024 | Jacobs et al. 2022 | Medication Adherence Rating Scale-5 | MARS-5 | Validated | Yes | No | No | No | No | N/A |
| Ganna et al. 2024 | Park et al. 2022a | Diary | NR | NR | Yes | No | No | No | No | N/A |
| Ganna et al. 2024 | Ream et al. 2021 | Endocrine Therapy Medication Usage Questionnaire | ETMUQ | Validated | Yes | No | No | No | No | N/A |
| Ganna et al. 2024 | Tan et al. 2020 | Simplified Medication Adherence Questionnaire | SMAQ | Validated | Yes | No | No | No | No | N/A |
| Greer et al. 2016 [25] | Bhattacharya et al. 2012 | Beliefs About Medicines Questionnaire | BMQ | Validated | Yes | No | No | No | No | N/A |
| Greer et al. 2016 | Bhattacharya et al. 2012 | Medication Adherence Rating Scale | MARS | Validated | Yes | No | No | No | No | N/A |
| Greer et al. 2016 | Brier et al. 2015 | Single Item Measure | NR | NR | Yes | No | No | No | No | N/A |
| Greer et al. 2016 | Brier et al. 2015 | Morisky Medication Adherence Scale-8 | MMAS-8 | Adapted | Yes | No | No | No | No | N/A |
| Greer et al. 2016 | Brier et al. 2015 | Visual Analog Scale | VAS | NR | Yes | No | No | No | No | N/A |
| Greer et al. 2016 | Efficace et al. 2012 | Morisky Medication Adherence Scale | MMAS | Adapted | Yes | No | No | No | No | N/A |
| Greer et al. 2016 | Font et al. 2012 | Single Item Measure | NR | NR | Yes | No | No | No | No | N/A |
| Greer et al. 2016 | Gebbia et al. 2013 | Basel Assessment of Adherence Scale | BAAS | Validated | Yes | No | No | No | No | N/A |
| Greer et al. 2016 | Gebbia et al. 2013 | Visual Analog Scale | VAS | NR | Yes | No | No | No | No | N/A |
| Greer et al. 2016 | Grunfeld et al. 2005 | Beliefs About Medicines Questionnaire | BMQ | Validated | Yes | No | No | No | No | N/A |
| Greer et al. 2016 | Grunfeld et al. 2005 | Medication Adherence Rating Scale-5 | MARS-5 | Validated | Yes | No | No | No | No | N/A |
| Greer et al. 2016 | Grunfeld et al. 2005 | Single Item Measure | NR | NR | Yes | No | No | No | No | N/A |
| Greer et al. 2016 | Hadji et al. 2013 | Morisky Medication Adherence Scale-4 | MMAS-4 | Adapted | Yes | No | No | No | No | N/A |
| Greer et al. 2016 | Jönsson et al. 2012 | Morisky Medication Adherence Scale-9 | MMAS-9 | Validated | Yes | No | No | No | No | N/A |
| Greer et al. 2016 | Macintosh et al. 2007 | Questionnaire | NR | NR | Yes | No | No | No | No | N/A |
| Greer et al. 2016 | Macintosh et al. 2007 | Diary | NR | NR | Yes | No | No | No | No | N/A |
| Greer et al. 2016 | Mayer et al. 2009 | Diary | NR | NR | Yes | No | No | No | No | N/A |
| Greer et al. 2016 | Mazzeo et al. 2011 | Visual Analog Scale | VAS | NR | Yes | No | No | No | No | N/A |
| Greer et al. 2016 | Neven et al. 2014 | Single Item Measure | NR | NR | Yes | No | No | No | No | N/A |
| Greer et al. 2016 | Noens et al. 2009 | Basel Assessment of Adherence Scale | BAAS | Validated | Yes | No | No | No | No | N/A |
| Greer et al. 2016 | Noens et al. 2009 | Visual Analog Scale | VAS | Validated | Yes | No | No | No | No | N/A |
| Greer et al. 2016 | Oberguggenberger et al. 2012 | Simplified Medication Adherence Questionnaire | SMAQ | Validated | Yes | No | No | No | No | N/A |
| Greer et al. 2016 | Ruddy et al. 2012 | Diary | NR | NR | Yes | No | No | No | No | N/A |
| Greer et al. 2016 | Schneider et al. 2014 | Questionnaire | NR | Not Validated | Yes | No | No | No | No | N/A |
| Greer et al. 2016 | Simon et al. 2014 | Questionnaire | NR | NR | Yes | No | No | No | No | N/A |
| Greer et al. 2016 | Sommers et al. 2012 | Morisky Medication Adherence Scale-8 | MMAS-8 | Validated | Yes | No | No | No | No | N/A |
| Greer et al. 2016 | Stanton et al. 2014 | Morisky Medication Adherence Scale-4 | MMAS-4 | Adapted | Yes | No | No | No | No | N/A |
| Greer et al. 2016 | Winterhalder et al. 2011 | Diary | NR | NR | Yes | No | No | No | No | N/A |
| Greer et al. 2016 | Xu et al. 2012 | Questionnaire | NR | NR | Yes | No | No | No | No | N/A |
| Greer et al. 2016 | Ziller et al. 2009 | Questionnaire | NR | Validated | Yes | No | No | No | No | N/A |
| Greer et al. 2016 | Ziller et al. 2009 | Questionnaire | NR | Validated | Yes | No | No | No | No | N/A |
| Greer et al. 2016 | Ziller et al. 2013 | Questionnaire | NR | Not Validated | Yes | No | No | No | No | N/A |
| Haddad et al. 2021 [26] | Akbaba et al. 2019 | Questionnaire | NR | Not Validated | No | No | No | No | Yes | Dilator use |
| Haddad et al. 2021 | Alban et al. 2019 | Questionnaire | NR | NR | No | No | No | No | Yes | Dilator use |
| Haddad et al. 2021 | Foerster et al. 2016 | Questionnaire | NR | NR | No | No | No | No | Yes | Dilator use |
| Haddad et al. 2021 | Friedman et al. 2009 | Questionnaire | NR | NR | No | No | No | No | Yes | Dilator use |
| Haddad et al. 2021 | Park et al. 2015 | Questionnaire | NR | NR | No | No | No | No | Yes | Dilator use |
| Haddad et al. 2021 | Punt 2011 | Questionnaire | NR | NR | No | No | No | No | Yes | Dilator use |
| Hajjar & Rezazadeh 2020 [27] | Kim et al. 2011 | Alternate Healthy Eating Index | AHEI | Validated | No | Yes | No | No | No | N/A |
| Hajjar & Rezazadeh 2020 | Kim et al. 2011 | Alternate Mediterranean Diet score | aMED | Validated | No | Yes | No | No | No | N/A |
| Hajjar & Rezazadeh 2020 | Kim et al. 2011 | Diet Quality Index-Revised | DQIR | Validated | No | Yes | No | No | No | N/A |
| Hajjar & Rezazadeh 2020 | Kim et al. 2011 | Food Frequencies Questionnaire | FFQ | Validated | No | Yes | No | No | No | N/A |
| Hajjar & Rezazadeh 2020 | Kim et al. 2011 | Recommended Food Score | RFS | Validated | No | Yes | No | No | No | N/A |
| Hajjar & Rezazadeh 2020 | Kyrø et al. 2013 | Food Frequencies Questionnaire | FFQ | Validated | No | Yes | No | No | No | N/A |
| Hajjar & Rezazadeh 2020 | Lassale et al. 2016 | Healthy Eating Index 2010 | HEI-2010 | Validated | No | Yes | No | No | No | N/A |
| Hajjar & Rezazadeh 2020 | Lassale et al. 2016 | Diet Quality Index International | DQI-I | Validated | No | Yes | No | No | No | N/A |
| Hajjar & Rezazadeh 2020 | Lassale et al. 2016 | Dietary Approaches to Stop Hypertension | DASH | Validated | No | Yes | No | No | No | N/A |
| Hajjar & Rezazadeh 2020 | Lassale et al. 2016 | Healthy Lifestyle Index | HLI | Validated | No | Yes | No | No | No | N/A |
| Hajjar & Rezazadeh 2020 | Lassale et al. 2016 | Healthy Nordic Food Index | HNFI | Validated | No | Yes | No | No | No | N/A |
| Hajjar & Rezazadeh 2020 | Lassale et al. 2016 | Mediterranean Diet Score | MDS | Validated | No | Yes | No | No | No | N/A |
| Hajjar & Rezazadeh 2020 | Lassale et al. 2016 | Mediterranean Style Dietary Pattern Score | MSDPS | Validated | No | Yes | No | No | No | N/A |
| Hajjar & Rezazadeh 2020 | Lassale et al. 2016 | Relative Med diet score | rMED | Validated | No | Yes | No | No | No | N/A |
| Hajjar & Rezazadeh 2020 | Lassale et al. 2016 | WHO Healthy Diet Indicator | WHO HDI | Validated | No | Yes | No | No | No | N/A |
| Hajjar & Rezazadeh 2020 | Lassale et al. 2016 | World Cancer Research Fund | WCRF | Validated | No | Yes | No | No | No | N/A |
| Hajjar & Rezazadeh 2020 | Lemming et al. 2018 | Healthy Nordic Food Index | HNFI | Validated | No | Yes | No | No | No | N/A |
| Hajjar & Rezazadeh 2020 | Lemming et al. 2018 | Modified Mediterranean Diet Score | MMDS | Validated | No | Yes | No | No | No | N/A |
| Hajjar & Rezazadeh 2020 | Li et al. 2015 | Healthy Nordic Food Index | HNFI | Validated | No | Yes | No | No | No | N/A |
| Hajjar & Rezazadeh 2020 | Ratjen et al. 2018 | Food Frequencies Questionnaire | FFQ | Validated | No | Yes | No | No | No | N/A |
| Hajjar & Rezazadeh 2020 | Roswall et al. 2015 | Healthy Nordic Food Index | HNFI | Validated | No | Yes | No | No | No | N/A |
| Hajjar & Rezazadeh 2020 | Sharma et al. 2018 | Food Frequencies Questionnaire | FFQ | Validated | No | Yes | No | No | No | N/A |
| Haynes et al. 2021 [28] | Abrahao et al. 2018 | Dental Care Score | NR | NR | No | No | No | Yes | No | N/A |
| Haynes et al. 2021 | Chang et al. 2013 | Oral Hygiene Score | NR | Validated | No | No | No | Yes | No | N/A |
| Haynes et al. 2021 | Farquhar et al. 2017 | Single Item Measure | NR | NR | No | No | No | Yes | No | N/A |
| Haynes et al. 2021 | Friemel et al. 2016 | Dental Care Score | NR | NR | No | No | No | Yes | No | N/A |
| Heiney et al. 2018 [29] | Albert et al. 2011 | Questionnaire | NR | NR | Yes | No | No | No | No | N/A |
| Heiney et al. 2018 | Arriola et al. 2014 | Medication Adherence Rating Scale-10 | MARS-10 | Validated | Yes | No | No | No | No | N/A |
| Heiney et al. 2018 | Arriola et al. 2014 | Drug Attittude Inventory | DAI | Validated | Yes | No | No | No | No | N/A |
| Heiney et al. 2018 | Hadji et al. 2013 | Morisky Medication Adherence Scale-4 | MMAS-4 | Adapted | Yes | No | No | No | No | N/A |
| Heiney et al. 2018 | Heisig et al. 2015 | Questionnaire | NR | Validated | Yes | No | No | No | No | N/A |
| Heiney et al. 2018 | Kahn et al. 2007 | Questionnaire | NR | NR | Yes | No | No | No | No | N/A |
| Heiney et al. 2018 | Lin et al. 2017 | Medication Adherence Rating Scale-4 | MMAS-4 | Validated | Yes | No | No | No | No | N/A |
| Heiney et al. 2018 | Liu et al. 2013 | Questionnaire | NR | NR | Yes | No | No | No | No | N/A |
| Heiney et al. 2018 | Markopoulos et al. 2015 | Single Item Measure | NR | NR | Yes | No | No | No | No | N/A |
| Heiney et al. 2018 | Ziller et al. 2013 | Questionnaire | NR | Not Validated | Yes | No | No | No | No | N/A |
| Heiney et al. 2021 [30] | Kekale et al. 2016 | Morisky Medication Adherence Scale-8 | MMAS-8 | Validated | Yes | No | No | No | No | N/A |
| Heiney et al. 2021 | Santoleri et al. 2018 | Diary | NR | NR | Yes | No | No | No | No | N/A |
| Horne et al. 2013 [31] | Bhattacharya et al. 2012 | Beliefs About Medicines Questionnaire | BMQ | Validated | Yes | No | No | No | No | N/A |
| Horne et al. 2013 | Bhattacharya et al. 2012 | Medication Adherence Rating Scale | MARS | Validated | Yes | No | No | No | No | N/A |
| Horne et al. 2013 | Grunfeld et al. 2005 | Beliefs About Medicines Questionnaire | BMQ | Validated | Yes | No | No | No | No | N/A |
| Horne et al. 2013 | Grunfeld et al. 2005 | Medication Adherence Rating Scale-5 | MARS-5 | Validated | Yes | No | No | No | No | N/A |
| Horne et al. 2013 | Grunfeld et al. 2005 | Single Item Measure | NR | NR | Yes | No | No | No | No | N/A |
| Huang et al. 2016 [32] | Almeida et al. 2013 | Morisky Medication Adherence Scale-4 | MMAS-4 | Validated | Yes | No | No | No | No | N/A |
| Huang et al. 2016 | Almeida et al. 2013 | Diary | NR | NR | Yes | No | No | No | No | N/A |
| Huang et al. 2016 | Bhattacharya et al. 2012 | Medication Adherence Rating Scale | MARS | Validated | Yes | No | No | No | No | N/A |
| Huang et al. 2016 | Bhattacharya et al. 2012 | Beliefs About Medicines Questionnaire | BMQ | Validated | Yes | No | No | No | No | N/A |
| Huang et al. 2016 | Daouphars et al. 2013 | Questionnaire | NR | Validated | Yes | No | No | No | No | N/A |
| Huang et al. 2016 | Efficace et al. 2012 | Morisky Medication Adherence Scale | MMAS | Adapted | Yes | No | No | No | No | N/A |
| Huang et al. 2016 | Gebbia et al. 2013 | Basel Assessment of Adherence Scale | BAAS | Validated | Yes | No | No | No | No | N/A |
| Huang et al. 2016 | Gebbia et al. 2013 | Visual Analog Scale | VAS | NR | Yes | No | No | No | No | N/A |
| Huang et al. 2016 | Grunfeld et al. 2005 | Beliefs About Medicines Questionnaire | BMQ | Validated | Yes | No | No | No | No | N/A |
| Huang et al. 2016 | Grunfeld et al. 2005 | Medication Adherence Rating Scale-5 | MARS-5 | Validated | Yes | No | No | No | No | N/A |
| Huang et al. 2016 | Grunfeld et al. 2005 | Single Item Measure | NR | NR | Yes | No | No | No | No | N/A |
| Huang et al. 2016 | Jönsson et al. 2012 | Morisky Medication Adherence Scale-9 | MMAS-9 | Validated | Yes | No | No | No | No | N/A |
| Huang et al. 2016 | Kekale et al. 2014 | Morisky Medication Adherence Scale-8 | MMAS-8 | Validated | Yes | No | No | No | No | N/A |
| Huang et al. 2016 | Noens et al. 2009 | Basel Assessment of Adherence Scale | BAAS | Validated | Yes | No | No | No | No | N/A |
| Huang et al. 2016 | Noens et al. 2009 | Visual Analog Scale | VAS | Validated | Yes | No | No | No | No | N/A |
| Huang et al. 2016 | Saratsiotou et al. 2010 | Questionnaire | NR | NR | Yes | No | No | No | No | N/A |
| Huang et al. 2016 | Sommers et al. 2012 | Morisky Medication Adherence Scale-8 | MMAS-8 | Validated | Yes | No | No | No | No | N/A |
| Hurtado-de-Mendoza et al. 2016 [33] | Hadji et al. 2013 | Morisky Medication Adherence Scale-4 | MMAS-4 | Adapted | Yes | No | No | No | No | N/A |
| Hurtado-de-Mendoza et al. 2016 | Neven et al. 2014 | Single Item Measure | NR | NR | Yes | No | No | No | No | N/A |
| Hurtado-de-Mendoza et al. 2016 | Ziller et al. 2013 | Questionnaire | NR | Not Validated | Yes | No | No | No | No | N/A |
| Kaptein et al. 2021 [34] | Bright et al. 2016 | Morisky Medication Adherence Scale-8 | MMAS-8 | Adapted | Yes | No | No | No | No | N/A |
| Kaptein et al. 2021 | Buzaglo et al. 2017 | Questionnaire | NR | Not Validated | Yes | No | No | No | No | N/A |
| Kaptein et al. 2021 | Corter et al. 2018 | Beliefs About Medicines Questionnaire | BMQ | Adapted | Yes | No | No | No | No | N/A |
| Kaptein et al. 2021 | Font et al. 2017 | Simplified Medication Adherence Questionnaire | SMAQ | Validated | Yes | No | No | No | No | N/A |
| Kaptein et al. 2021 | Hefner et al. 2018 | Medication Adherence Rating Scale-5 | MARS-5 | Validated | Yes | No | No | No | No | N/A |
| Kaptein et al. 2021 | Hefner et al. 2018 | Beliefs About Medicines Questionnaire | BMQ | Validated | Yes | No | No | No | No | N/A |
| Kaptein et al. 2021 | Hurtado-de-Mendoza et al. 2019a | Morisky Medication Adherence Scale | MMAS | Adapted | Yes | No | No | No | No | N/A |
| Kaptein et al. 2021 | Jacobs et al. 2017 | Cancer Therapy Satisfaction Questionnaire | CTSQ | Validated | Yes | No | No | No | No | N/A |
| Kaptein et al. 2021 | Karmakar et al. 2017 | Morisky Medication Adherence Scale-8 | MMAS-8 | Validated | Yes | No | No | No | No | N/A |
| Kaptein et al. 2021 | Kimmick et al. 2015 | Morisky Medication Adherence Scale-8 | MMAS-8 | Validated | Yes | No | No | No | No | N/A |
| Kaptein et al. 2021 | Markovitz et al. 2017 | Morisky Medication Adherence Scale-4 | MMAS-4 | Validated | Yes | No | No | No | No | N/A |
| Kaptein et al. 2021 | Mols et al. 2020 | Medication Adherence Rating Scale-5 | MARS-5 | Validated | Yes | No | No | No | No | N/A |
| Kaptein et al. 2021 | Moon et al. 2017 | Medication Adherence Rating Scale-5 | MARS-5 | Validated | Yes | No | No | No | No | N/A |
| Kaptein et al. 2021 | Moon et al. 2019a | Medication Adherence Rating Scale-5 | MARS-5 | Validated | Yes | No | No | No | No | N/A |
| Kaptein et al. 2021 | Pan et al. 2018 | Single Item Measure | NR | Validated | Yes | No | No | No | No | N/A |
| Kaptein et al. 2021 | Walker et al. 2016 | Morisky Medication Adherence Scale | MMAS | Adapted | Yes | No | No | No | No | N/A |
| Kavookjian & Wittayanukorn 2015 [35] | Richardson et al. 1988 | Questionnaire | NR | NR | Yes | No | No | No | No | N/A |
| Kearney et al. 2022 [36] | Schwartz & Winters-Stone 2009 | Diary | NR | NR | No | No | Yes | No | No | N/A |
| Kearney et al. 2022 | van Waart et al. 2015 | Diary | NR | NR | No | No | Yes | No | No | N/A |
| Krekeler et al. 2018 [37] | Cnossen et al. 2014 | Diary | NR | NR | No | No | Yes | No | No | N/A |
| Krekeler et al. 2018 | Cnossen et al. 2016 | Diary | NR | NR | No | No | Yes | No | No | N/A |
| Krekeler et al. 2018 | Duarte et al. 2013 | Diary | NR | NR | No | No | Yes | No | No | N/A |
| Krekeler et al. 2018 | Hajdu et al. 2017 | Diary | NR | NR | No | No | Yes | No | No | N/A |
| Krekeler et al. 2018 | Low et al. 2001 | Single Item Measure | NR | NR | No | Yes | Yes | No | No | N/A |
| Krekeler et al. 2018 | Wall et al. 2016 | Diary | NR | NR | No | No | Yes | No | No | N/A |
| Lafata et al. 2022 [38] | Gebbia et al. 2013 | Visual Analog Scale | VAS | NR | Yes | No | No | No | No | N/A |
| Lafata et al. 2022 | Gebbia et al. 2013 | Basel Assessment of Adherence Scale | BAAS | Validated | Yes | No | No | No | No | N/A |
| Lafata et al. 2022 | Kekale et al. 2016 | Morisky Medication Adherence Scale-8 | MMAS-8 | Validated | Yes | No | No | No | No | N/A |
| Lafata et al. 2022 | Krikorian et al. 2019 | Beliefs About Medicines Questionnaire | BMQ | Validated | Yes | No | No | No | No | N/A |
| Lafata et al. 2022 | Morgan et al. 2018 | Chew et al.’s health literacy questionnaire | NR | Adapted | Yes | No | No | No | No | N/A |
| Lafata et al. 2022 | Muluneh et al. 2018a | Single Item Measure | NR | NR | Yes | No | No | No | No | N/A |
| Lafata et al. 2022 | Todo M et al. 2019 | Diary | NR | NR | Yes | No | No | No | No | N/A |
| Lafata et al. 2022 | Tokdemir et al. 2017 | Medication Adherence Self-Efficacy Scale | MASES | Validated | Yes | No | No | No | No | N/A |
| Lasala & Santoleri 2021 [39] | Al-Dewik et al. 2016 | Morisky Medication Adherence Scale-9 | MMAS-9 | Validated | Yes | No | No | No | No | N/A |
| Lasala & Santoleri 2021 | Banna et al. 2020 | Basel Assessment of Adherence Scale | BAAS | Adapted | Yes | No | No | No | No | N/A |
| Lasala & Santoleri 2021 | de Oliveira et al. 2004 | Questionnaire | NR | NR | Yes | No | No | No | No | N/A |
| Lasala & Santoleri 2021 | Garcia- Queiruga 2016 | Morisky Medication Adherence Scale-8 | MMAS-8 | Validated | Yes | No | No | No | No | N/A |
| Lasala & Santoleri 2021 | Gupta et al. 2018 | Morisky Medication Adherence Scale-4 | MMAS-4 | Validated | Yes | No | No | No | No | N/A |
| Lasala & Santoleri 2021 | Jönsson et al. 2012 | Morisky Medication Adherence Scale-9 | MMAS-9 | Validated | Yes | No | No | No | No | N/A |
| Lasala & Santoleri 2021 | Le Saux et al. 2018 | Questionnaire | NR | NR | Yes | No | No | No | No | N/A |
| Lasala & Santoleri 2021 | Mulu Fentie et al. 2019 | Morisky Medication Adherence Scale-8 | MMAS-8 | Adapted | Yes | No | No | No | No | N/A |
| Lasala & Santoleri 2021 | Patel et al. 2013 | Single Item Measure | NR | NR | Yes | No | No | No | No | N/A |
| Lasala & Santoleri 2021 | Tsai et al. 2018 | Morisky Medication Adherence Scale-8 | MMAS-8 | Validated | Yes | No | No | No | No | N/A |
| Lasala & Santoleri 2021 | Unnikrishnan et al. 2016 | Morisky Medication Adherence Scale-8 | MMAS-8 | Validated | Yes | No | No | No | No | N/A |
| Lasala & Santoleri 2021 | Xu et al. 2012 | Questionnaire | NR | NR | Yes | No | No | No | No | N/A |
| Lasala & Santoleri 2021 | Yanamandra et al. 2017 | Morisky Medication Adherence Scale-9 | MMAS-9 | Adapted | Yes | No | No | No | No | N/A |
| Lazzari et al. 2021 [40] | Rief et al. 2014 | Diary | NR | NR | No | No | Yes | No | No | N/A |
| Lee 2018 [41] | Antosh et al. 2013 | Diary | NR | NR | No | No | No | No | Yes | Dilator use |
| Lee 2018 | Brand & Stenlake 2012 | Questionnaire | NR | NR | No | No | No | No | Yes | Dilator use |
| Lee 2018 | Edmonds et al. 2012 | Questionnaire | NR | NR | No | No | No | No | Yes | Dilator use |
| Lee 2018 | Friedmann et al. 2011 | Single Item Measure | NR | NR | No | No | No | No | Yes | Dilator use |
| Lee 2018 | Law et al. 2013 | Diary | NR | NR | No | No | No | No | Yes | Dilator use |
| Lee 2018 | Punt 2011 | Questionnaire | NR | NR | No | No | No | No | Yes | Dilator use |
| Lin et al. 2017 [42] | Arriola et al. 2014 | Medication Adherence Rating Scale-10 | MARS-10 | Validated | Yes | No | No | No | No | N/A |
| Lin et al. 2017 | Arriola et al. 2014 | Drug Attittude Inventory | DAI | Validated | Yes | No | No | No | No | N/A |
| Lin et al. 2017 | Bhatta et al. 2013 | Single Item Measure | NR | Validated | Yes | No | No | No | No | N/A |
| Lin et al. 2017 | Kahn et al. 2007 | Questionnaire | NR | NR | Yes | No | No | No | No | N/A |
| Lin et al. 2017 | Kimmick et al. 2015 | Morisky Medication Adherence Scale-8 | MMAS-8 | Validated | Yes | No | No | No | No | N/A |
| Lin et al. 2017 | Kirk & Hudis 2008 | Questionnaire | NR | Not Validated | Yes | No | No | No | No | N/A |
| Lin et al. 2017 | Lash et al. 2006 | Questionnaire | NR | NR | Yes | No | No | No | No | N/A |
| Lin et al. 2017 | Lebovits et al. 1990 | Questionnaire | NR | NR | Yes | No | No | No | No | N/A |
| Lin et al. 2017 | Liu et al. 2013 | Questionnaire | NR | NR | Yes | No | No | No | No | N/A |
| Lin et al. 2017 | Manning & Bettencourt 2011 | Questionnaire | NR | Adapted | Yes | No | No | No | No | N/A |
| Lin et al. 2017 | Walker et al. 2016 | Morisky Medication Adherence Scale | MMAS | Adapted | Yes | No | No | No | No | N/A |
| Luo et al. 2022 [43] | Kim et al. 2018 | Medication Adherence Rating Scale | K-MARS | Validated | Yes | No | No | No | No | N/A |
| Luo et al. 2022 | Liao et al. 2017 | Functional Exercise Adherence Scale | FEAS | Validated | No | No | Yes | No | No | N/A |
| Luo et al. 2022 | Qin 2018 | Functional Exercise Adherence Scale | FEAS | Validated | No | No | Yes | No | No | N/A |
| Luo et al. 2022 | Shi 2018 | Functional Exercise Adherence Scale | FEAS | Validated | No | No | Yes | No | No | N/A |
| Luo et al. 2022 | Uhm et al. 2017 | International Physical Activity Questionnaire, short form | IPAQ-SF | Validated | No | No | Yes | No | No | N/A |
| Ma et al. 2020 [44] | Edbrooke et al. 2019 | Diary | NR | NR | No | No | Yes | No | No | N/A |
| Mathes et al. 2014 [45] | Atkins & Fallowfield 2006 | Questionnaire | NR | NR | Yes | No | No | No | No | N/A |
| Mathes et al. 2014 | Bhattacharya et al. 2012 | Beliefs About Medicines Questionnaire | BMQ | Validated | Yes | No | No | No | No | N/A |
| Mathes et al. 2014 | Bhattacharya et al. 2012 | Medication Adherence Rating Scale | MARS | Validated | Yes | No | No | No | No | N/A |
| Mathes et al. 2014 | Grunfeld et al. 2005 | Beliefs About Medicines Questionnaire | BMQ | Validated | Yes | No | No | No | No | N/A |
| Mathes et al. 2014 | Grunfeld et al. 2005 | Medication Adherence Rating Scale-5 | MARS-5 | Validated | Yes | No | No | No | No | N/A |
| Mathes et al. 2014 | Grunfeld et al. 2005 | Single Item Measure | NR | NR | Yes | No | No | No | No | N/A |
| Mathes et al. 2014 | Larizza et al. 2006 | Morisky Medication Adherence Scale-4 | MMAS-4 | Validated | Yes | No | No | No | No | N/A |
| Mathes et al. 2014 | Lebovits et al. 1990 | Questionnaire | NR | NR | No | No | Yes | No | No | N/A |
| Mathes et al. 2014 | Marques & Pierin 2008 | Morisky Medication Adherence Scale-4 | MMAS-4 | Validated | Yes | No | No | No | No | N/A |
| Mathes et al. 2014 | Saratsiotou et al. 2010 | Questionnaire | NR | NR | Yes | No | No | No | No | N/A |
| Mausbach et al. 2015 [46] | Lash et al. 2006 | Questionnaire | NR | NR | Yes | No | No | No | No | N/A |
| Mausbach et al. 2015 | Manning & Bettencourt 2011 | Questionnaire | NR | Adapted | Yes | No | No | No | No | N/A |
| Mausbach et al. 2015 | Stanton et al. 2014 | Morisky Medication Adherence Scale-4 | MMAS-4 | Adapted | Yes | No | No | No | No | N/A |
| Mc Grath et al. 2022 [47] | Newton et al. 2011 | Diary | NR | NR | No | No | Yes | No | No | N/A |
| Mc Grath et al. 2022 | Von Gruenigen et al. 2011 | Diary | NR | NR | No | Yes | Yes | No | No | N/A |
| Mc Grath et al. 2022 | Zhang et al. 2017 | Diary | NR | NR | No | No | Yes | No | No | N/A |
| Mc Grath et al. 2022 | Zhang et al. 2018 | Diary | NR | NR | No | No | Yes | No | No | N/A |
| Mc Grath et al. 2022 | Zhou et al. 2017 | Diary | NR | NR | No | No | Yes | No | No | N/A |
| McGrady & Pai 2019 [48] | Pizzo et al. 1983 | Diary | NR | NR | Yes | No | No | No | No | N/A |
| McGrady & Pai 2019 | Unnikrishnan et al. 2016 | Morisky Medication Adherence Scale-8 | MMAS-8 | Validated | Yes | No | No | No | No | N/A |
| McHugh et al. 2024 [49] | Baguley et al. 2021 | Mediterranean Diet Adherence Score | MEDAS | Validated | No | Yes | No | No | No | N/A |
| McHugh et al. 2024 | Baguley et al. 2022 | Mediterranean Diet Adherence Score | MEDAS | Validated | No | Yes | No | No | No | N/A |
| McHugh et al. 2024 | Braakhuis et al. 2017 | Diary | NR | NR | No | Yes | No | No | No | N/A |
| McHugh et al. 2024 | Braakhuis et al. 2017 | Mediterranean Diet Adherence Score | MEDAS | Validated | No | Yes | No | No | No | N/A |
| McHugh et al. 2024 | Bruno et al. 2021 | Diary | NR | NR | No | Yes | No | No | No | N/A |
| McHugh et al. 2024 | Cho et al. 2020 | Mediterranean Diet Adherence Score | MEDAS | Validated | No | Yes | No | No | No | N/A |
| McHugh et al. 2024 | Gioxari et al. 2021 | Food Frequencies Questionnaire | FFQ | Validated | No | Yes | No | No | No | N/A |
| McHugh et al. 2024 | Harvie et al. 2019 | Diary | NR | NR | No | Yes | No | No | No | N/A |
| McHugh et al. 2024 | Kleckner et al. 2022 | Mediterranean Diet Adherence Score | MEDAS | Validated | No | Yes | No | No | No | N/A |
| McHugh et al. 2024 | Papandreou et al. 2021 | Food Frequencies Questionnaire | FFQ | Validated | No | Yes | Yes | No | No | N/A |
| McHugh et al. 2024 | Papandreou et al. 2021 | Diary | NR | NR | No | Yes | No | No | No | N/A |
| McHugh et al. 2024 | Parma et al. 2022 | Mediterranean Diet Adherence Score | MEDAS | Validated | No | Yes | No | No | No | N/A |
| McHugh et al. 2024 | Ruiz-Vozmediano et al. 2020 | Mediterranean Diet Adherence Score | MEDAS | Validated | No | Yes | No | No | No | N/A |
| McHugh et al. 2024 | Skouroliakou et al. 2017 | Food Frequencies Questionnaire | FFQ | Validated | No | Yes | No | No | No | N/A |
| McHugh et al. 2024 | Skouroliakou et al. 2017 | Diary | NR | NR | No | Yes | No | No | No | N/A |
| McHugh et al. 2024 | Villarini et al. 2012 | Diary | NR | NR | No | Yes | No | No | No | N/A |
| McHugh et al. 2024 | Zuniga et al. 2019 | Questionnaire | NR | Validated | No | Yes | No | No | No | N/A |
| McHugh et al. 2024 | Zuniga et al. 2019 | Spice And Herbs Questionnaire | NR | Validated | No | Yes | No | No | No | N/A |
| Molenaar et al. 2023 [50] | Bousquet-Dion et al. 2018 | Diary | NR | NR | No | Yes | No | No | No | N/A |
| Molenaar et al. 2023 | Bousquet-Dion et al. 2018 | Diary | NR | NR | No | No | Yes | No | No | N/A |
| Molenaar et al. 2023 | Carli et al. 2020 | Diary | NR | NR | No | No | Yes | No | No | N/A |
| Molenaar et al. 2023 | Carli et al. 2020 | Diary | NR | NR | No | Yes | No | No | No | N/A |
| Molenaar et al. 2023 | Gillis et al. 2014 | Diary | NR | NR | No | No | Yes | No | No | N/A |
| Molenaar et al. 2023 | Gillis et al. 2014 | Diary | NR | NR | No | Yes | No | No | No | N/A |
| Moon et al. 2017 [51] | Arriola et al. 2014 | Medication Adherence Rating Scale-10 | MARS-10 | Validated | Yes | No | No | No | No | N/A |
| Moon et al. 2017 | Arriola et al. 2014 | Drug Attittude Inventory | DAI | Validated | Yes | No | No | No | No | N/A |
| Moon et al. 2017 | Bhatta et al. 2013 | Single Item Measure | NR | Validated | Yes | No | No | No | No | N/A |
| Moon et al. 2017 | Friese et al. 2013 | Single Item Measure | NR | NR | Yes | No | No | No | No | N/A |
| Moon et al. 2017 | Grunfeld et al. 2005 | Beliefs About Medicines Questionnaire | BMQ | Validated | Yes | No | No | No | No | N/A |
| Moon et al. 2017 | Grunfeld et al. 2005 | Medication Adherence Rating Scale-5 | MARS-5 | Validated | Yes | No | No | No | No | N/A |
| Moon et al. 2017 | Grunfeld et al. 2005 | Single Item Measure | NR | NR | Yes | No | No | No | No | N/A |
| Moon et al. 2017 | Kahn et al. 2007 | Questionnaire | NR | NR | Yes | No | No | No | No | N/A |
| Moon et al. 2017 | Karmakar 2013 | Morisky Medication Adherence Scale-8 | MMAS-8 | Validated | Yes | No | No | No | No | N/A |
| Moon et al. 2017 | Kimmick et al. 2015 | Morisky Medication Adherence Scale-8 | MMAS-8 | Validated | Yes | No | No | No | No | N/A |
| Moon et al. 2017 | Liu et al. 2013 | Questionnaire | NR | NR | Yes | No | No | No | No | N/A |
| Moon et al. 2017 | Livaudais et al. 2013 | Breast Cancer Treatment Questionnaire | NR | NR | Yes | No | No | No | No | N/A |
| Moon et al. 2017 | Schover et al. 2014 | 3-item Adherence Estimator | NR | Validated | Yes | No | No | No | No | N/A |
| Moon et al. 2017 | Stanton et al. 2014 | Morisky Medication Adherence Scale-4 | MMAS-4 | Adapted | Yes | No | No | No | No | N/A |
| Moon et al. 2017 | Tinari et al. 2015 | Questionnaire | NR | NR | Yes | No | No | No | No | N/A |
| Moon et al. 2017 | Wouters et al. 2014 | Morisky Medication Adherence Scale-8 | MMAS-8 | Validated | Yes | No | No | No | No | N/A |
| Moon et al. 2017 | Wouters et al. 2014 | Medication Adherence Rating Scale-5 | MARS-5 | Validated | Yes | No | No | No | No | N/A |
| Moon et al. 2017 | Wouters et al. 2014 | Questionnaire | NR | NR | Yes | No | No | No | No | N/A |
| Mullan et al. 2023 [52] | McLaughlin et al. 2020 | Diary | NR | NR | No | No | Yes | No | Yes | Wearing Compression |
| Mullan et al. 2023 | Yao et al. 2020 | Food Frequencies Questionnaire | FFQ | NR | No | Yes | No | No | No | N/A |
| Murphy et al. 2012 [53] | Grunfeld et al. 2005 | Beliefs About Medicines Questionnaire | BMQ | Validated | Yes | No | No | No | No | N/A |
| Murphy et al. 2012 | Grunfeld et al. 2005 | Medication Adherence Rating Scale-5 | MARS-5 | Validated | Yes | No | No | No | No | N/A |
| Murphy et al. 2012 | Grunfeld et al. 2005 | Single Item Measure | NR | NR | Yes | No | No | No | No | N/A |
| Naser et al. 2022 [54] | Arber et al. 2017 | Questionnaire | NR | NR | Yes | No | No | No | No | N/A |
| Naser et al. 2022 | Cransac et al. 2019 | Questionnaire | NR | Validated | Yes | No | No | No | No | N/A |
| Naser et al. 2022 | Gupta et al. 2018 | Morisky Medication Adherence Scale-4 | MMAS-4 | Validated | Yes | No | No | No | No | N/A |
| Naser et al. 2022 | Silveira et al. 2021 | Treatment Adherence Measure | TAM | Validated | Yes | No | No | No | No | N/A |
| Naser et al. 2022 | Solano et al. 2021 | Girerd Medication Adherence Scale | NR | Validated | Yes | No | No | No | No | N/A |
| Noens et al. 2014 [55] | Doti et al. 2008 | Diary | NR | NR | Yes | No | No | No | No | N/A |
| Noens et al. 2014 | Eliasson et al. 2011 | Single Item Measure | NR | NR | Yes | No | No | No | No | N/A |
| Noens et al. 2014 | Gupta et al. 2011 | Questionnaire | NR | NR | Yes | No | No | No | No | N/A |
| Noens et al. 2014 | Johnson et al. 2010 | Questionnaire | NR | NR | Yes | No | No | No | No | N/A |
| Noens et al. 2014 | Jönsson et al. 2012 | Morisky Medication Adherence Scale-9 | MMAS-9 | Validated | Yes | No | No | No | No | N/A |
| Noens et al. 2014 | Kong et al. 2006 | Morisky Medication Adherence Scale-4 | MMAS-4 | Validated | Yes | No | No | No | No | N/A |
| Noens et al. 2014 | Koren-Michowitz et al. 2010 | Diary | NR | NR | Yes | No | No | No | No | N/A |
| Noens et al. 2014 | Noens et al. 2009 | Visual Analog Scale | VAS | Validated | Yes | No | No | No | No | N/A |
| Noens et al. 2014 | Noens et al. 2009 | Basel Assessment of Adherence Scale | BAAS | Validated | Yes | No | No | No | No | N/A |
| Oldenmenger et al. 2018 [56] | Anderson et al. 2004 | Questionnaire | NR | NR | Yes | No | No | No | No | N/A |
| Oldenmenger et al. 2018 | Jahn et al. 2014 | Morisky Medication Adherence Scale-4 | MMAS-4 | Validated | Yes | No | No | No | No | N/A |
| Oldenmenger et al. 2018 | Lin et al. 2006 | Morisky Medication Adherence Scale-4 | MMAS-4 | Validated | Yes | No | No | No | No | N/A |
| Oldenmenger et al. 2018 | Miaskowski et al. 2004 | Diary | NR | NR | Yes | No | No | No | No | N/A |
| Oldenmenger et al. 2018 | Oliver et al. 2001 | Single Item Measure | NR | NR | Yes | No | No | No | No | N/A |
| Onvani et al. 2016 [57] | Akbaraly et al. 2011 | Alternate Healthy Eating Index | AHEI | Validated | No | Yes | No | No | No | N/A |
| Onvani et al. 2016 | Fung et al. 2014 | Alternate Healthy Eating Index 2010 | AHEI-2010 | Validated | No | Yes | No | No | No | N/A |
| Onvani et al. 2016 | Fung et al. 2014 | Alternate Mediterranean Diet score | aMED | Validated | No | Yes | No | No | No | N/A |
| Onvani et al. 2016 | Fung et al. 2014 | Dietary Approaches to Stop Hypertension | DASH | Validated | No | Yes | No | No | No | N/A |
| Onvani et al. 2016 | Fung et al. 2014 | Semi Quantitative Food Frequency Questionnaire | SFFQ | NR | No | Yes | No | No | No | N/A |
| Onvani et al. 2016 | George et al. 2014a | Healthy Eating Index-2010 | HEI-2010 | Validated | No | Yes | No | No | No | N/A |
| Onvani et al. 2016 | Harmon et al. 2015 | Alternate Healthy Eating Index 2010 | AHEI-2010 | Validated | No | Yes | No | No | No | N/A |
| Onvani et al. 2016 | Harmon et al. 2015 | Healthy Eating Index-2010 | HEI-2010 | Validated | No | Yes | No | No | No | N/A |
| Onvani et al. 2016 | Izano et al. 2013 | Alternate Healthy Eating Index 2010 | AHEI-2010 | Validated | No | Yes | No | No | No | N/A |
| Onvani et al. 2016 | Izano et al. 2013 | Dietary Approaches to Stop Hypertension | DASH | Validated | No | Yes | No | No | No | N/A |
| Onvani et al. 2016 | Izano et al. 2013 | Food Frequencies Questionnaire | FFQ | Validated | No | Yes | No | No | No | N/A |
| Onvani et al. 2016 | Kapppeler et al. 2013 | Healthy Eating Index | HEI | Validated | No | Yes | No | No | No | N/A |
| Onvani et al. 2016 | Mursu et al. 2013 | Alternate Healthy Eating Index 2010 | AHEI-2010 | Validated | No | Yes | No | No | No | N/A |
| Onvani et al. 2016 | Reedy et al. 2014 | Healthy Eating Index-2010 | HEI-2010 | Validated | No | Yes | No | No | No | N/A |
| Onvani et al. 2016 | Reedy et al. 2014 | Alternate Healthy Eating Index 2010 | AHEI-2010 | Validated | No | Yes | No | No | No | N/A |
| Onvani et al. 2016 | Thomson et al. 2014 | Healthy Eating Index-2005 | HEI-2005 | Validated | No | Yes | No | No | No | N/A |
| Onvani et al. 2016 | Thomson et al. 2014 | Women’s Health Initiative Food Frequencies Questionnaire | WHI FFQ | Validated | No | Yes | No | No | No | N/A |
| Onvani et al. 2016 | Yu et al. 2014 | Alternate Healthy Eating Index 2010 | AHEI-2010 | Validated | No | Yes | No | No | No | N/A |
| Onvani et al. 2016 | Yu et al. 2015 | Healthy Eating Index-2010 | HEI-2010 | Validated | No | Yes | No | No | No | N/A |
| Ormel et al. 2017 [58] | Arem et al. 2016 | Diary | NR | NR | No | No | Yes | No | No | N/A |
| Ormel et al. 2017 | Kampshoff et al. 2016 | Diary | NR | NR | No | No | Yes | No | No | N/A |
| Ormel et al. 2017 | Kuehl et al. 2016 | Diary | NR | NR | No | No | Yes | No | No | N/A |
| Ormel et al. 2017 | Latka et al. 2009 | Diary | NR | NR | No | No | Yes | No | No | N/A |
| Ormel et al. 2017 | McGuire et al. 2011 | Diary | NR | NR | No | No | Yes | No | No | N/A |
| Ormel et al. 2017 | Pinto et al. 2009 | Diary | NR | NR | No | No | Yes | No | No | N/A |
| Ormel et al. 2017 | Shang et al. 2012 | Diary | NR | NR | No | No | Yes | No | No | N/A |
| Parjanjpe et al. 2019 [59] | Brett et al. 2018 | Medication Adherence Rating Scale-5 | MARS-5 | Validated | Yes | No | No | No | No | N/A |
| Parjanjpe et al. 2019 | Bright et al. 2016 | Morisky Medication Adherence Scale-8 | MMAS-8 | Adapted | Yes | No | No | No | No | N/A |
| Parjanjpe et al. 2019 | Karmakar et al. 2017 | Morisky Medication Adherence Scale-8 | MMAS-8 | Validated | Yes | No | No | No | No | N/A |
| Parjanjpe et al. 2019 | Liu et al. 2013 | Questionnaire | NR | NR | Yes | No | No | No | No | N/A |
| Parjanjpe et al. 2019 | Salgado et al. 2017 | Single Item Measure | NR | NR | Yes | No | No | No | No | N/A |
| Parjanjpe et al. 2019 | Stanton et al. 2014 | Morisky Medication Adherence Scale-4 | MMAS-4 | Adapted | Yes | No | No | No | No | N/A |
| Parjanjpe et al. 2019 | Tinari et al. 2015 | Questionnaire | NR | NR | Yes | No | No | No | No | N/A |
| Pezzolato et al. 2023 [60] | Arch et al. 2022 | Single Item Measure | NR | Validated | Yes | No | No | No | No | N/A |
| Pezzolato et al. 2023 | Chalela et al. 2018 | Morisky Medication Adherence Scale-4 | MMAS-4 | Validated | Yes | No | No | No | No | N/A |
| Pezzolato et al. 2023 | Getachew et al. 2022 | Simplified Medication Adherence Questionnaire | SMAQ | Validated | Yes | No | No | No | No | N/A |
| Pezzolato et al. 2023 | Graetz et al. 2018 | Medication Adherence Reasons | MARS-1 | Validated | Yes | No | No | No | No | N/A |
| Pezzolato et al. 2023 | Graetz et al. 2018 | Morisky Medication Adherence Scale-4 | MMAS-4 | Validated | Yes | No | No | No | No | N/A |
| Pezzolato et al. 2023 | Hadji et al. 2013 | Morisky Medication Adherence Scale-4 | MMAS-4 | Adapted | Yes | No | No | No | No | N/A |
| Pezzolato et al. 2023 | Heisig et al. 2015 | Questionnaire | NR | Validated | Yes | No | No | No | No | N/A |
| Pezzolato et al. 2023 | Jacobs et al. 2022 | Medication Adherence Rating Scale-5 | MARS-5 | Validated | Yes | No | No | No | No | N/A |
| Pezzolato et al. 2023 | Krok-Schoen et al. 2019 | Morisky Medication Adherence Scale | MMAS | Adapted | Yes | No | No | No | No | N/A |
| Pezzolato et al. 2023 | Mamguem Kamga et al. 2020 | Morisky Medication Adherence Scale-4 | MMAS-4 | Validated | Yes | No | No | No | No | N/A |
| Pezzolato et al. 2023 | Moon et al. 2019b | Medication Adherence Rating Scale-5 | MARS-5 | Validated | Yes | No | No | No | No | N/A |
| Pezzolato et al. 2023 | Mougalian et al. 2017 | Single Item Measure | NR | NR | Yes | No | No | No | No | N/A |
| Pezzolato et al. 2023 | Myers et al. 2022 | Domains of Subjective Extent of Nonadherence | DOSE | Validated | Yes | No | No | No | No | N/A |
| Pezzolato et al. 2023 | Paladino et al. 2019 | Health Beliefs and Medication Adherence in Breast Cancer | HBMABC | Validated | Yes | No | No | No | No | N/A |
| Pezzolato et al. 2023 | Ream et al. 2021 | Endocrine Therapy Medication Usage Questionnaire | ETMUQ | Validated | Yes | No | No | No | No | N/A |
| Pezzolato et al. 2023 | Shelby et al. 2019 | Morisky Medication Adherence Scale-8 | MMAS-8 | Adapted | Yes | No | No | No | No | N/A |
| Pezzolato et al. 2023 | Smith et al. 2022 | Adherence Starts with Knowledge-12 | ASK-12 | Validated | Yes | No | No | No | No | N/A |
| Pezzolato et al. 2023 | Tan et al. 2020 | Simplified Medication Adherence Questionnaire | SMAQ | Validated | Yes | No | No | No | No | N/A |
| Pezzolato et al. 2023 | Von Blanckenburg et al. 2013 | Medication Adherence Rating Scale | MARS-D | Validated | Yes | No | No | No | No | N/A |
| Pezzolato et al. 2023 | Yanez et al. 2022 | Adherence to Refills and Medications Scale | ARMS | Validated | Yes | No | No | No | No | N/A |
| Pezzolato et al. 2023 | Ziller et al. 2013 | Questionnaire | NR | Not Validated | Yes | No | No | No | No | N/A |
| Puspitasari et al. 2020 [61] | Bhattacharya et al. 2012 | Beliefs About Medicines Questionnaire | BMQ | Validated | Yes | No | No | No | No | N/A |
| Puspitasari et al. 2020 | Bhattacharya et al. 2012 | Medication Adherence Rating Scale | MARS | Validated | Yes | No | No | No | No | N/A |
| Puspitasari et al. 2020 | Hefner et al. 2018 | Medication Adherence Rating Scale-5 | MARS-5 | Validated | Yes | No | No | No | No | N/A |
| Puspitasari et al. 2020 | Hefner et al. 2018 | Beliefs About Medicines Questionnaire | BMQ | Validated | Yes | No | No | No | No | N/A |
| Puspitasari et al. 2020 | Kawakami et al. 2015 | Diary | NR | NR | Yes | No | No | No | No | N/A |
| Puspitasari et al. 2020 | Timmers et al. 2012 | Medication Adherence Rating scale-6 | MARS-6 | Validated | Yes | No | No | No | No | N/A |
| Puspitasari et al. 2020 | Winterhalder et al. 2011 | Diary | NR | NR | Yes | No | No | No | No | N/A |
| Puspitasari et al. 2020 | Zahrina et al. 2014 | Medication Compliance Questionnaire | MCQ | Validated | Yes | No | No | No | No | N/A |
| Puspitasari et al. 2021 [62] | Graetz et al. 2018 | Medication Adherence Reasons | MARS-1 | Validated | Yes | No | No | No | No | N/A |
| Puspitasari et al. 2021 | Graetz et al. 2018 | Morisky Medication Adherence Scale-4 | MMAS-4 | Validated | Yes | No | No | No | No | N/A |
| Puspitasari et al. 2021 | Greer et al. 2020 | Morisky Medication Adherence Scale-4 | MMAS-4 | Validated | Yes | No | No | No | No | N/A |
| Puspitasari et al. 2021 | Kim et al. 2014 | Medication Adherence Rating Scale | K-MARS | Validated | Yes | No | No | No | No | N/A |
| Puspitasari et al. 2021 | Krok-Schoen et al. 2019 | Morisky Medication Adherence Scale | MMAS | Adapted | Yes | No | No | No | No | N/A |
| Puspitasari et al. 2022 [63] | Birand et al. 2019 | Beliefs About Medicines Questionnaire | BMQ | Validated | Yes | No | No | No | No | N/A |
| Puspitasari et al. 2022 | Birand et al. 2019 | Morisky Medication Adherence Scale-4 | MMAS-4 | Validated | Yes | No | No | No | No | N/A |
| Puts et al. 2014 [64] | Lash et al. 2006 | Questionnaire | NR | NR | Yes | No | No | No | No | N/A |
| Puts et al. 2014 | Ruddy et al. 2010 | Diary | NR | NR | Yes | No | No | No | No | N/A |
| Puts et al. 2014 | Winterhalder et al. 2011 | Diary | NR | NR | Yes | No | No | No | No | N/A |
| Puts et al. 2014 | Ziller et al. 2009 | Questionnaire | NR | Validated | Yes | No | No | No | No | N/A |
| Ramos et al. 2025 [65] | Cadmus et al. 2009 | 7-day Physical Activity Log | 7-day PAL | Validated | No | No | Yes | No | No | N/A |
| Ramos et al. 2025 | Farajivafa et al. 2023 | Diary | NR | NR | No | Yes | Yes | No | No | N/A |
| Ramos et al. 2025 | Gokal et al. 2016 | Diary | NR | NR | No | No | Yes | No | No | N/A |
| Ramos et al. 2025 | Huang et al. 2019 | Diary | NR | NR | No | No | Yes | No | No | N/A |
| Ramos et al. 2025 | Husebø et al. 2014 | Diary | NR | NR | No | No | Yes | No | No | N/A |
| Ramos et al. 2025 | Kilgour et al. 2008 | Diary | NR | NR | No | Yes | Yes | No | No | N/A |
| Ramos et al. 2025 | Yuen & Sword 2007 | Diary | NR | NR | No | No | Yes | No | No | N/A |
| Schwedhelm et al. 2016 [66] | Aarts et al. 2013 | Questionnaire | NR | NR | No | Yes | Yes | Yes | No | N/A |
| Schwedhelm et al. 2016 | Arthur et al. 2013 | Food Frequencies Questionnaire | FFQ | Validated | No | Yes | No | No | No | N/A |
| Schwedhelm et al. 2016 | Bao et al. 2015 | Questionnaire | NR | NR | Yes | No | Yes | No | No | N/A |
| Schwedhelm et al. 2016 | Bao et al. 2015 | Questionnaire | NR | NR | No | Yes | No | Yes | No | N/A |
| Schwedhelm et al. 2016 | Battaglioli et al. 2006 | Questionnaire | NR | Validated | No | Yes | No | Yes | No | N/A |
| Schwedhelm et al. 2016 | Beasley et al. 2011 | Food Frequencies Questionnaire | FFQ | Validated | No | Yes | No | No | No | N/A |
| Schwedhelm et al. 2016 | Boffetta et al. 1997 | Questionnaire | NR | NR | No | Yes | No | Yes | No | N/A |
| Schwedhelm et al. 2016 | Dal Maso et al. 2008 | Food Frequencies Questionnaire | FFQ | Validated | No | Yes | No | No | No | N/A |
| Schwedhelm et al. 2016 | Dal Maso et al. 2008 | Questionnaire | NR | NR | No | No | Yes | Yes | No | N/A |
| Schwedhelm et al. 2016 | Duffy et al. 2009 | Willett Food Frequency Questionnaire | Willet FFQ | Validated | No | Yes | No | No | No | N/A |
| Schwedhelm et al. 2016 | Duffy et al. 2009 | Alcohol Use Disorders Identification Test | NR | Validated | No | Yes | No | No | No | N/A |
| Schwedhelm et al. 2016 | Ewertz et al. 1991 | Food Frequencies Questionnaire | FFQ | NR | No | Yes | No | No | No | N/A |
| Schwedhelm et al. 2016 | Ferronha et al. 2012 | Food Frequencies Questionnaire | FFQ | Adapted | No | Yes | No | No | No | N/A |
| Schwedhelm et al. 2016 | Fink et al. 2006 | Block Food Frequencies Questionnaire | Block FFQ | Adapted | No | Yes | No | No | No | N/A |
| Schwedhelm et al. 2016 | Flatt et al. 2010 | Arizona Food Frequency Questionnaire | AFFQ | Validated | No | Yes | No | No | No | N/A |
| Schwedhelm et al. 2016 | Franceschi et al. 2009 | Food Frequencies Questionnaire | FFQ | NR | No | Yes | No | No | No | N/A |
| Schwedhelm et al. 2016 | Fuchs et al. 2014 | Food Frequencies Questionnaire | FFQ | NR | No | Yes | No | No | No | N/A |
| Schwedhelm et al. 2016 | Fung et al. 2014 | Alternate Healthy Eating Index 2010 | AHEI-2010 | Validated | No | Yes | No | No | No | N/A |
| Schwedhelm et al. 2016 | Fung et al. 2014 | Alternate Mediterranean Diet score | aMED | Validated | No | Yes | No | No | No | N/A |
| Schwedhelm et al. 2016 | Fung et al. 2014 | Dietary Approaches to Stop Hypertension | DASH | Validated | No | Yes | No | No | No | N/A |
| Schwedhelm et al. 2016 | Fung et al. 2014 | Semi Quantitative Food Frequency Questionnaire | SFFQ | NR | No | Yes | No | No | No | N/A |
| Schwedhelm et al. 2016 | George et al. 2011 | Healthy Eating Index 2005 | HEI-2005 | Validated | No | Yes | No | No | No | N/A |
| Schwedhelm et al. 2016 | George et al. 2011 | Women’s Health Initiative Food Frequencies Questionnaire | WHI FFQ | Validated | No | Yes | No | No | No | N/A |
| Schwedhelm et al. 2016 | George et al. 2014b | Healthy Eating Index 2005 | HEI-2005 | Validated | No | Yes | No | No | No | N/A |
| Schwedhelm et al. 2016 | George et al. 2014b | Women’s Health Initiative Food Frequencies Questionnaire | WHI FFQ | Validated | No | Yes | No | No | No | N/A |
| Schwedhelm et al. 2016 | [Goodwin et al. 2003](about:blank) | Block Food Frequencies Questionnaire | Block FFQ | Validated | No | Yes | No | No | No | N/A |
| Schwedhelm et al. 2016 | Guercio et al. 2015 | Food Frequencies Questionnaire | FFQ | NR | No | Yes | No | No | No | N/A |
| Schwedhelm et al. 2016 | Harris & Wolk 2012 | Food Frequencies Questionnaire | FFQ | NR | No | Yes | No | No | No | N/A |
| Schwedhelm et al. 2016 | Harris & Wolk 2012 | Food Frequencies Questionnaire | FFQ | NR | No | Yes | No | No | No | N/A |
| Schwedhelm et al. 2016 | Hebert et al. 1998 | Food Frequencies Questionnaire | FFQ | NR | No | Yes | No | No | No | N/A |
| Schwedhelm et al. 2016 | Holmes et al. 1999 | Food Frequencies Questionnaire | FFQ | Validated | No | Yes | No | No | No | N/A |
| Schwedhelm et al. 2016 | Inoue-Choi et al. 2013 | Harvard Food Frequency Questionnaire | Harvard FFQ | Adapted | No | Yes | No | No | No | N/A |
| Schwedhelm et al. 2016 | Izano et al. 2013 | Alternate Healthy Eating Index 2010 | AHEI-2010 | Validated | No | Yes | No | No | No | N/A |
| Schwedhelm et al. 2016 | Izano et al. 2013 | Dietary Approaches to Stop Hypertension | DASH | Validated | No | Yes | No | No | No | N/A |
| Schwedhelm et al. 2016 | Izano et al. 2013 | Food Frequencies Questionnaire | FFQ | Validated | No | Yes | No | No | No | N/A |
| Schwedhelm et al. 2016 | Kenfield et al. 2014 | Mediterranean Diet Score | MDS | Validated | No | Yes | No | No | No | N/A |
| Schwedhelm et al. 2016 | Kenfield et al. 2014 | Semi Quantitative Food Frequency Questionnaire | SFFQ | Validated | No | Yes | No | No | No | N/A |
| Schwedhelm et al. 2016 | Khankari et al. 2015 | Block Food Frequencies Questionnaire | Block FFQ | Adapted | No | Yes | No | No | No | N/A |
| Schwedhelm et al. 2016 | Kim et al. 2011 | Alternate Healthy Eating Index | AHEI | Validated | No | Yes | No | No | No | N/A |
| Schwedhelm et al. 2016 | Kim et al. 2011 | Alternate Mediterranean Diet score | aMED | Validated | No | Yes | No | No | No | N/A |
| Schwedhelm et al. 2016 | Kim et al. 2011 | Diet Quality Index-Revised | DQIR | Validated | No | Yes | No | No | No | N/A |
| Schwedhelm et al. 2016 | Kim et al. 2011 | Food Frequencies Questionnaire | FFQ | Validated | No | Yes | No | No | No | N/A |
| Schwedhelm et al. 2016 | Kim et al. 2011 | Recommended Food Score | RFS | Validated | No | Yes | No | No | No | N/A |
| Schwedhelm et al. 2016 | Kroenke et al. 2005 | Food Frequencies Questionnaire | FFQ | Validated | No | Yes | No | No | No | N/A |
| Schwedhelm et al. 2016 | Kroenke et al. 2013 | Fred Hutchinson Cancer Research Center Food Questionnaire | FHCRC-FFQ | Validated | No | Yes | No | No | No | N/A |
| Schwedhelm et al. 2016 | Kwan et al. 2009 | Fred Hutchinson Cancer Research Center Food Questionnaire | FHCRC-FFQ | Validated | No | Yes | No | No | No | N/A |
| Schwedhelm et al. 2016 | Kwan et al. 2010 | Fred Hutchinson Cancer Research Center Food Questionnaire | FHCRC-FFQ | Validated | No | Yes | No | No | No | N/A |
| Schwedhelm et al. 2016 | Kwan et al. 2013 | Fred Hutchinson Cancer Research Center Food Questionnaire | FHCRC-FFQ | Validated | No | Yes | No | No | No | N/A |
| Schwedhelm et al. 2016 | Kwan et al. 2013 | Arizona Food Frequency Questionnaire | AFFQ | Validated | No | Yes | No | No | No | N/A |
| Schwedhelm et al. 2016 | Larsen et al. 2015 | Food Frequencies Questionnaire | FFQ | Validated | No | Yes | No | No | No | N/A |
| Schwedhelm et al. 2016 | Leo et al. 2015 | Food Frequencies Questionnaire | FFQ | NR | No | Yes | No | No | No | N/A |
| Schwedhelm et al. 2016 | [Lochhead et al. 2015](about:blank) | Food Frequencies Questionnaire | FFQ | Validated | No | Yes | No | No | No | N/A |
| Schwedhelm et al. 2016 | Meyerhardt et al. 2007 | Food Frequencies Questionnaire | FFQ | NR | No | Yes | No | No | No | N/A |
| Schwedhelm et al. 2016 | Nagle et al. 2003 | Nurses’ Health Study Questionnaire | NR | Adapted | No | Yes | No | No | No | N/A |
| Schwedhelm et al. 2016 | Nechuta et al. 2013 | Food Frequencies Questionnaire | FFQ | Validated | No | Yes | No | No | No | N/A |
| Schwedhelm et al. 2016 | Nechuta et al. 2016 | Food Frequencies Questionnaire | FFQ | NR | No | Yes | No | No | No | N/A |
| Schwedhelm et al. 2016 | Newcomb et al. 2013 | Questionnaire | NR | NR | No | Yes | No | No | No | N/A |
| Schwedhelm et al. 2016 | Ollberding et al. 2013 | Health Habits and History Questionnaire | HHHQ | Adapted | No | Yes | No | No | No | N/A |
| Schwedhelm et al. 2016 | Pelucchi et al. 2014 | Questionnaire | NR | NR | No | Yes | No | Yes | No | N/A |
| Schwedhelm et al. 2016 | Pettersson et al. 2012 | Food Frequencies Questionnaire | FFQ | Validated | No | Yes | No | No | No | N/A |
| Schwedhelm et al. 2016 | Richman et al. 2011 | Food Frequencies Questionnaire | FFQ | NR | No | Yes | No | No | No | N/A |
| Schwedhelm et al. 2016 | Rohan et al. 1993 | Food Frequencies Questionnaire | FFQ | NR | No | Yes | No | No | No | N/A |
| Schwedhelm et al. 2016 | Sandoval et al. 2009 | Questionnaire | NR | NR | No | Yes | No | No | No | N/A |
| Schwedhelm et al. 2016 | Shen et al. 2012 | Arizona Tea Questionnaire | NR | Validated | No | Yes | No | No | No | N/A |
| Schwedhelm et al. 2016 | Shen et al. 2012 | Arizona Smoking Assessment Questionnaire | NR | Adapted | No | No | No | Yes | No | N/A |
| Schwedhelm et al. 2016 | Shen et al. 2012 | Arizona Food Frequency Questionnaire | AFFQ | Validated | No | Yes | No | No | No | N/A |
| Schwedhelm et al. 2016 | Thomson et al. 2014 | Healthy Eating Index-2005 | HEI-2005 | Validated | No | Yes | No | No | No | N/A |
| Schwedhelm et al. 2016 | Thomson et al. 2014 | Women’s Health Initiative Food Frequencies Questionnaire | WHI FFQ | Validated | No | Yes | No | No | No | N/A |
| Schwedhelm et al. 2016 | Vrieling et al. 2013 | Food Frequencies Questionnaire | FFQ | NR | No | Yes | No | No | No | N/A |
| Schwedhelm et al. 2016 | Yang et al. 2014 | Food Frequencies Questionnaire | FFQ | NR | Yes | Yes | No | No | No | N/A |
| Schwedhelm et al. 2016 | Yang et al. 2015 | Food Frequencies Questionnaire | FFQ | NR | No | Yes | No | No | No | N/A |
| Schwedhelm et al. 2016 | Zhang et al. 2004 | Questionnaire | NR | NR | No | Yes | No | Yes | No | N/A |
| Schwedhelm et al. 2016 | Zhu et al. 2013 | Hawaii Food Frequencies Questionnaire | Hawaii FFQ | Adapted | No | Yes | No | No | No | N/A |
| Setyowibowo et al. 2022 [67] | Ruiz-Vozmediano et al. 2020 | Mediterranean Diet Adherence Score | MEDAS | Validated | No | Yes | No | No | No | N/A |
| Skrabal et al. 2021 [68] | Geissler et al. 2017 | Morisky Medication Adherence Scale-8 | MMAS-8 | Validated | Yes | No | No | No | No | N/A |
| Skrabal et al. 2021 | Muluneh et al. 2018b | Chew et al.’s health literacy questionnaire | NR | Adapted | Yes | No | No | No | No | N/A |
| Sotirova et al. 2021 [69] | Cnossen et al. 2016 | Diary | NR | NR | No | No | Yes | No | No | N/A |
| Sotirova et al. 2021 | Kanera et al. 2016 | Short Questionnaire to Assess Health Enhancing Physical Activity | SQUASH | Validated | No | No | Yes | No | No | N/A |
| Sotirova et al. 2021 | Kanera et al. 2016 | Dutch Measuring Instruments for Research on Smoking and Smoking Cessation | NR | Validated | No | No | No | Yes | No | N/A |
| Sotirova et al. 2021 | Kanera et al. 2016 | Dutch Standard Questionnaire on Food Consumption | NR | Adapted | No | Yes | No | No | No | N/A |
| Sotirova et al. 2021 | Kanera et al. 2017 | Short Questionnaire to Assess Health Enhancing Physical Activity | SQUASH | Validated | No | No | Yes | No | No | N/A |
| Sotirova et al. 2021 | Kanera et al. 2017 | Dutch Standard Questionnaire on Food Consumption | NR | Adapted | No | Yes | No | No | No | N/A |
| Sotirova et al. 2021 | Lee et al. 2013 | Diary | NR | NR | No | Yes | Yes | No | No | N/A |
| Sotirova et al. 2021 | Lee et al. 2014 | Diary | NR | NR | No | Yes | Yes | No | No | N/A |
| Spei et al. 2023 [70] | Al Ramadhani et al. 2021 | American Cancer Society Score | ACS Score | Validated | No | Yes | No | No | No | N/A |
| Spei et al. 2023 | Al Ramadhani et al. 2021 | Alternate Healthy Eating Index 2010 | AHEI-2010 | Validated | No | Yes | No | No | No | N/A |
| Spei et al. 2023 | Al Ramadhani et al. 2021 | Diet Quality Index | DQI | Validated | No | Yes | No | No | No | N/A |
| Spei et al. 2023 | Al Ramadhani et al. 2021 | Healthy Eating Index 2010 | HEI-2010 | Validated | No | Yes | No | No | No | N/A |
| Spei et al. 2023 | Al Ramadhani et al. 2021 | World Cancer Research Fund | WCRF | Validated | No | Yes | No | No | No | N/A |
| Spei et al. 2023 | Ergas et al. 2021 | Alternate Mediterranean Diet score | aMED | Validated | No | Yes | No | No | No | N/A |
| Spei et al. 2023 | Ergas et al. 2021 | American Cancer Society Score | ACS Score | Validated | No | Yes | No | No | No | N/A |
| Spei et al. 2023 | Ergas et al. 2021 | Block 2005 Food Frequency Questionnaire | Block 2005 FFQ | Validated | No | Yes | No | No | No | N/A |
| Spei et al. 2023 | Ergas et al. 2021 | Dietary Approaches to Stop Hypertension | DASH | Validated | No | Yes | No | No | No | N/A |
| Spei et al. 2023 | Ergas et al. 2021 | Healthy Eating Index-2015 | HEI-2015 | Validated | No | Yes | No | No | No | N/A |
| Spei et al. 2023 | Fung et al. 2014 | Alternate Healthy Eating Index 2010 | AHEI-2010 | Validated | No | Yes | No | No | No | N/A |
| Spei et al. 2023 | Fung et al. 2014 | Alternate Mediterranean Diet score | aMED | Validated | No | Yes | No | No | No | N/A |
| Spei et al. 2023 | Fung et al. 2014 | Dietary Approaches to Stop Hypertension | DASH | Validated | No | Yes | No | No | No | N/A |
| Spei et al. 2023 | Fung et al. 2014 | Semi Quantitative Food Frequency Questionnaire | SFFQ | NR | No | Yes | No | No | No | N/A |
| Spei et al. 2023 | George et al. 2011 | Healthy Eating Index 2005 | HEI-2005 | Validated | No | Yes | No | No | No | N/A |
| Spei et al. 2023 | George et al. 2011 | Women’s Health Initiative Food Frequencies Questionnaire | WHI FFQ | Validated | No | Yes | No | No | No | N/A |
| Spei et al. 2023 | George et al. 2014b | Healthy Eating Index 2005 | HEI-2005 | Validated | No | Yes | No | No | No | N/A |
| Spei et al. 2023 | George et al. 2014b | Women’s Health Initiative Food Frequencies Questionnaire | WHI FFQ | Validated | No | Yes | No | No | No | N/A |
| Spei et al. 2023 | Guinter et al. 2018 | American Cancer Society Score | ACS Score | Validated | No | Yes | No | No | No | N/A |
| Spei et al. 2023 | Guinter et al. 2018 | Dietary Approaches to Stop Hypertension | DASH | Validated | No | Yes | No | No | No | N/A |
| Spei et al. 2023 | Guinter et al. 2018 | Food Frequencies Questionnaire | FFQ | Validated | No | Yes | No | No | No | N/A |
| Spei et al. 2023 | Izano et al. 2013 | Alternate Healthy Eating Index 2010 | AHEI-2010 | Validated | No | Yes | No | No | No | N/A |
| Spei et al. 2023 | Izano et al. 2013 | Dietary Approaches to Stop Hypertension | DASH | Validated | No | Yes | No | No | No | N/A |
| Spei et al. 2023 | Izano et al. 2013 | Food Frequencies Questionnaire | FFQ | Validated | No | Yes | No | No | No | N/A |
| Spei et al. 2023 | Karavasiloglou et al. 2019 | Healthy Eating Index 2005 | HEI-2005 | Validated | No | Yes | No | No | No | N/A |
| Spei et al. 2023 | Karavasiloglou et al. 2019 | Mediterranean Diet Score | MDS | Validated | No | Yes | No | No | No | N/A |
| Spei et al. 2023 | Kenfield et al. 2014 | Mediterranean Diet Score | MDS | Validated | No | Yes | No | No | No | N/A |
| Spei et al. 2023 | Kenfield et al. 2014 | Semi Quantitative Food Frequency Questionnaire | SFFQ | Validated | No | Yes | No | No | No | N/A |
| Spei et al. 2023 | Kim et al. 2011 | Food Frequencies Questionnaire | FFQ | Validated | No | Yes | No | No | No | N/A |
| Spei et al. 2023 | Kim et al. 2011 | Alternate Healthy Eating Index | AHEI | Validated | No | Yes | No | No | No | N/A |
| Spei et al. 2023 | Kim et al. 2011 | Alternate Mediterranean Diet score | aMED | Validated | No | Yes | No | No | No | N/A |
| Spei et al. 2023 | Kim et al. 2011 | Diet Quality Index-Revised | DQIR | Validated | No | Yes | No | No | No | N/A |
| Spei et al. 2023 | Kim et al. 2011 | Recommended Food Score | RFS | Validated | No | Yes | No | No | No | N/A |
| Spei et al. 2023 | Kroenke et al. 2005 | Food Frequencies Questionnaire | FFQ | Validated | No | Yes | No | No | No | N/A |
| Spei et al. 2023 | Kwan et al. 2009 | Fred Hutchinson Cancer Research Center Food Questionnaire | FHCRC-FFQ | Validated | No | Yes | No | No | No | N/A |
| Spei et al. 2023 | McCullough et al. 2016 | Block Food Frequencies Questionnaire | Block FFQ | Adapted | No | Yes | No | No | No | N/A |
| Spei et al. 2023 | McCullough et al. 2016 | Harvard Food Frequency Questionnaire | Harvard FFQ | Adapted | No | Yes | No | No | No | N/A |
| Spei et al. 2023 | Meyerhardt et al. 2007 | Semi Quantitative Food Frequency Questionnaire | SFFQ | Validated | No | Yes | No | No | No | N/A |
| Spei et al. 2023 | Ratjen et al. 2017 | Food Frequencies Questionnaire | FFQ | Validated | No | Yes | No | No | No | N/A |
| Spei et al. 2023 | Ratjen et al. 2017 | Healthy Nordic Food Index | HNFI | Validated | No | Yes | No | No | No | N/A |
| Spei et al. 2023 | Ratjen et al. 2017 | Modified Mediterranean Diet Score | MMDS | Validated | No | Yes | No | No | No | N/A |
| Spei et al. 2023 | Sasamoto et al. 2022 | Alternate Healthy Eating Index | AHEI | Validated | No | Yes | No | No | No | N/A |
| Spei et al. 2023 | Sasamoto et al. 2022 | Alternate Healthy Eating Index 2010 | AHEI-2010 | Validated | No | Yes | No | No | No | N/A |
| Spei et al. 2023 | Sasamoto et al. 2022 | Empirical Dietary Inflammatory Pattern | EDIP | Validated | No | Yes | No | No | No | N/A |
| Spei et al. 2023 | Sun et al. 2018 | Healthy Eating Index 2010 | HEI-2010 | Validated | No | Yes | No | No | No | N/A |
| Spei et al. 2023 | Sun et al. 2018 | Women’s Health Initiative Food Frequencies Questionnaire | WHI FFQ | Validated | No | Yes | No | No | No | N/A |
| Spei et al. 2023 | Wang et al. 2020 | Food Frequencies Questionnaire | FFQ | Validated | No | Yes | No | No | No | N/A |
| Spei et al. 2023 | Wang et al. 2020 | Chinese Food Pagoda-2007 | CHFP-2007 | Validated | No | Yes | No | No | No | N/A |
| Spei et al. 2023 | Wang et al. 2020 | Chinese Food Pagoda-2016 | CHFP-2016 | Validated | No | Yes | No | No | No | N/A |
| Spei et al. 2023 | Wang et al. 2020 | Dietary Approaches to Stop Hypertension | DASH | Validated | No | Yes | No | No | No | N/A |
| Spei et al. 2023 | Wang et al. 2020 | Healthy Eating Index-2015 | HEI-2015 | Validated | No | Yes | No | No | No | N/A |
| Spei et al. 2023 | Yang et al. 2015 | Food Frequencies Questionnaire | FFQ | Adapted | No | Yes | No | No | No | N/A |
| Spence et al. 2010 [71] | Matthews et al. 2007 | Diary | NR | NR | No | No | Yes | No | No | N/A |
| Spence et al. 2010 | Thorsen et al. 2006 | Questionnaire | NR | NR | Yes | No | No | No | No | N/A |
| Stalsberg & Darvik 2024 [72] | Arem et al. 2016 | Diary | NR | NR | No | No | Yes | No | No | N/A |
| Stalsberg & Darvik 2024 | Cadmus et al. 2009 | 7-day Physical Activity Log | 7-day PAL | Validated | No | No | Yes | No | No | N/A |
| Stalsberg & Darvik 2024 | Carayol et al. 2019 | Diary | NR | NR | No | No | Yes | No | No | N/A |
| Stalsberg & Darvik 2024 | Demark-Wahnefried et al. 2008 | Diary | NR | NR | No | Yes | Yes | No | No | N/A |
| Stalsberg & Darvik 2024 | Dieli-Conwright et al. 2018 | 7-day Physical Activity Log | 7-day PAL | Validated | No | No | Yes | No | No | N/A |
| Stalsberg & Darvik 2024 | Dieli-Conwright et al. 2018 | Diary | NR | NR | No | Yes | No | No | No | N/A |
| Stalsberg & Darvik 2024 | Gokal et al. 2016 | Diary | NR | NR | No | No | Yes | No | No | N/A |
| Stalsberg & Darvik 2024 | Lund et al. 2019 | Diary | NR | NR | No | No | Yes | No | No | N/A |
| Stalsberg & Darvik 2024 | Matthews et al. 2007 | Diary | NR | NR | No | No | Yes | No | No | N/A |
| Stalsberg & Darvik 2024 | Mock et al. 2005 | Diary | NR | NR | No | No | Yes | No | No | N/A |
| Stalsberg & Darvik 2024 | Pickett et al. 2002 | Diary | NR | NR | No | No | Yes | No | No | N/A |
| Stalsberg & Darvik 2024 | Rogers et al. 2009 | Diary | NR | NR | No | No | Yes | No | No | N/A |
| Stalsberg & Darvik 2024 | Travier et al. 2015 | Diary | NR | NR | No | No | Yes | No | No | N/A |
| Stalsberg & Darvik 2024 | Vallance et al. 2016 | Diary | NR | NR | No | No | Yes | No | No | N/A |
| Stalsberg & Darvik 2024 | van Waart et al. 2015 | Diary | NR | NR | No | No | Yes | No | No | N/A |
| Stalsberg & Darvik 2024 | Wang et al. 2011 | Diary | NR | NR | No | No | Yes | No | No | N/A |
| Stalsberg & Darvik 2024 | Yang et al. 2011 | 7-Day Physical Activity Recall | 7D-PAR | Validated | No | No | Yes | No | No | N/A |
| Sun et al. 2024 [73] | Jacobs et al. 2022 | Medication Adherence Rating Scale-5 | MARS-5 | Validated | Yes | No | No | No | No | N/A |
| Sun et al. 2024 | Tan et al. 2020 | Simplified Medication Adherence Questionnaire | SMAQ | Validated | Yes | No | No | No | No | N/A |
| Sun et al. 2024 | Wang et al. 2017 | Morisky Medication Adherence Scale-8 | MMAS-8 | Validated | Yes | No | No | No | No | N/A |
| Sun et al. 2024 | Xu et al. 2020a | Morisky Medication Adherence Scale-8 | MMAS-8 | Validated | Yes | No | No | No | No | N/A |
| Sun et al. 2024 | Ziller et al. 2013 | Questionnaire | NR | Not Validated | Yes | No | No | No | No | N/A |
| Tan et al. 2021 [74] | Dennison et al. 2021 | Morisky Medication Adherence Scale-4 | MMAS-4 | Validated | Yes | No | No | No | No | N/A |
| Tan et al. 2021 | Kekale et al. 2016 | Morisky Medication Adherence Scale-8 | MMAS-8 | Validated | Yes | No | No | No | No | N/A |
| Tan et al. 2021 | Moulin et al. 2017 | Morisky Medication Adherence Scale-4 | MMAS-4 | Validated | Yes | No | No | No | No | N/A |
| Trauchburg et al. 2023 [75] | Al Ramadhani et al. 2021 | Alternate Healthy Eating Index 2010 | AHEI-2010 | Validated | No | Yes | No | No | No | N/A |
| Trauchburg et al. 2023 | Al Ramadhani et al. 2021 | American Cancer Society Score | ACS Score | Validated | No | Yes | No | No | No | N/A |
| Trauchburg et al. 2023 | Al Ramadhani et al. 2021 | Diet Quality Index | DQI | Validated | No | Yes | No | No | No | N/A |
| Trauchburg et al. 2023 | Al Ramadhani et al. 2021 | Healthy Eating Index 2010 | HEI-2010 | Validated | No | Yes | No | No | No | N/A |
| Trauchburg et al. 2023 | Al Ramadhani et al. 2021 | World Cancer Research Fund | WCRF | Validated | No | Yes | No | No | No | N/A |
| Trauchburg et al. 2023 | Anyene et al. 2021 | Block 2005 Food Frequency Questionnaire | Block 2005 FFQ, modified | Adapted | No | Yes | No | No | No | N/A |
| Trauchburg et al. 2023 | Arthur et al. 2013 | Harvard Food Frequency Questionnaire | Harvard FFQ | Validated | No | Yes | No | No | No | N/A |
| Trauchburg et al. 2023 | Deshmukh et al. 2018 | Questionnaire | NR | NR | No | Yes | No | No | No | N/A |
| Trauchburg et al. 2023 | Di Maso et al. 2020 | Mediterranean Diet Score | MDS | Validated | No | Yes | No | No | No | N/A |
| Trauchburg et al. 2023 | Di Maso et al. 2020 | Food Frequencies Questionnaire | FFQ | Validated | No | Yes | No | No | No | N/A |
| Trauchburg et al. 2023 | Ergas et al. 2021 | Alternate Mediterranean Diet score | aMED | Validated | No | Yes | No | No | No | N/A |
| Trauchburg et al. 2023 | Ergas et al. 2021 | American Cancer Society Score | ACS Score | Validated | No | Yes | No | No | No | N/A |
| Trauchburg et al. 2023 | Ergas et al. 2021 | Block 2005 Food Frequency Questionnaire | Block 2005 FFQ | Validated | No | Yes | No | No | No | N/A |
| Trauchburg et al. 2023 | Ergas et al. 2021 | Dietary Approaches to Stop Hypertension | DASH | Validated | No | Yes | No | No | No | N/A |
| Trauchburg et al. 2023 | Ergas et al. 2021 | Healthy Eating Index-2015 | HEI-2015 | Validated | No | Yes | No | No | No | N/A |
| Trauchburg et al. 2023 | Ferronha et al. 2012 | Food Frequencies Questionnaire | FFQ | Adapted | No | Yes | No | No | No | N/A |
| Trauchburg et al. 2023 | Fung et al. 2014 | Semi Quantitative Food Frequency Questionnaire | SFFQ | NR | No | Yes | No | No | No | N/A |
| Trauchburg et al. 2023 | Fung et al. 2014 | Alternate Healthy Eating Index 2010 | AHEI-2010 | Validated | No | Yes | No | No | No | N/A |
| Trauchburg et al. 2023 | Fung et al. 2014 | Alternate Mediterranean Diet score | aMED | Validated | No | Yes | No | No | No | N/A |
| Trauchburg et al. 2023 | Fung et al. 2014 | Dietary Approaches to Stop Hypertension | DASH | Validated | No | Yes | No | No | No | N/A |
| Trauchburg et al. 2023 | George et al. 2011 | Women’s Health Initiative Food Frequencies Questionnaire | WHI FFQ | Validated | No | Yes | No | No | No | N/A |
| Trauchburg et al. 2023 | George et al. 2011 | Healthy Eating Index 2005 | HEI-2005 | Validated | No | Yes | No | No | No | N/A |
| Trauchburg et al. 2023 | Guinter et al. 2018 | American Cancer Society Score | ACS Score | Validated | No | Yes | No | No | No | N/A |
| Trauchburg et al. 2023 | Guinter et al. 2018 | Dietary Approaches to Stop Hypertension | DASH | Validated | No | Yes | No | No | No | N/A |
| Trauchburg et al. 2023 | Guinter et al. 2018 | Food Frequencies Questionnaire | FFQ | Validated | No | Yes | No | No | No | N/A |
| Trauchburg et al. 2023 | Inoue-Choi et al. 2013 | Harvard Food Frequency Questionnaire | Harvard FFQ | Adapted | No | Yes | No | No | No | N/A |
| Trauchburg et al. 2023 | Izano et al. 2013 | Alternate Healthy Eating Index 2010 | AHEI-2010 | Validated | No | Yes | No | No | No | N/A |
| Trauchburg et al. 2023 | Izano et al. 2013 | Dietary Approaches to Stop Hypertension | DASH | Validated | No | Yes | No | No | No | N/A |
| Trauchburg et al. 2023 | Izano et al. 2013 | Food Frequencies Questionnaire | FFQ | Validated | No | Yes | No | No | No | N/A |
| Trauchburg et al. 2023 | Jacobs et al. 2016 | Healthy Eating Index 2010 | HEI 2010 | Validated | No | Yes | No | No | No | N/A |
| Trauchburg et al. 2023 | Jacobs et al. 2016 | Alternate Healthy Eating Index 2010 | AHEI-2010 | Validated | No | Yes | No | No | No | N/A |
| Trauchburg et al. 2023 | Jacobs et al. 2016 | Alternate Mediterranean Diet score | aMED | Validated | No | Yes | No | No | No | N/A |
| Trauchburg et al. 2023 | Jacobs et al. 2016 | Dietary Approaches to Stop Hypertension | DASH | Validated | No | Yes | No | No | No | N/A |
| Trauchburg et al. 2023 | Karavasiloglou et al. 2019 | Mediterranean Diet Score | MDS | Validated | No | Yes | No | No | No | N/A |
| Trauchburg et al. 2023 | Karavasiloglou et al. 2019 | Healthy Eating Index 2005 | HEI-2005 | Validated | No | Yes | No | No | No | N/A |
| Trauchburg et al. 2023 | Kenfield et al. 2014 | Mediterranean Diet Score | MDS | Validated | No | Yes | No | No | No | N/A |
| Trauchburg et al. 2023 | Kenfield et al. 2014 | Semi Quantitative Food Frequency Questionnaire | SFFQ | Validated | No | Yes | No | No | No | N/A |
| Trauchburg et al. 2023 | Kim et al. 2011 | Alternate Healthy Eating Index | AHEI | Validated | No | Yes | No | No | No | N/A |
| Trauchburg et al. 2023 | Kim et al. 2011 | Alternate Mediterranean Diet score | aMED | Validated | No | Yes | No | No | No | N/A |
| Trauchburg et al. 2023 | Kim et al. 2011 | Diet Quality Index-Revised | DQIR | Validated | No | Yes | No | No | No | N/A |
| Trauchburg et al. 2023 | Kim et al. 2011 | Food Frequencies Questionnaire | FFQ | Validated | No | Yes | No | No | No | N/A |
| Trauchburg et al. 2023 | Kim et al. 2011 | Recommended Food Score | RFS | Validated | No | Yes | No | No | No | N/A |
| Trauchburg et al. 2023 | Kroenke et al. 2005 | Food Frequencies Questionnaire | FFQ | Validated | No | Yes | No | No | No | N/A |
| Trauchburg et al. 2023 | Kwan et al. 2009 | Fred Hutchinson Cancer Research Center Food Questionnaire | FHCRC-FQ | Validated | No | Yes | No | No | No | N/A |
| Trauchburg et al. 2023 | Lee et al. 2020 | Food Frequencies Questionnaire | FFQ | Validated | No | Yes | No | No | No | N/A |
| Trauchburg et al. 2023 | Lei et al. 2021 | Food Frequencies Questionnaire | FFQ | Validated | No | Yes | No | No | No | N/A |
| Trauchburg et al. 2023 | Luo et al. 2020 | Chinese Healthy Eating Index | CHEI | Validated | No | Yes | No | No | No | N/A |
| Trauchburg et al. 2023 | Luo et al. 2020 | Healthy Eating Index-2015 | HEI-2015 | Validated | No | Yes | No | No | No | N/A |
| Trauchburg et al. 2023 | McCullough et al. 2016 | Harvard Food Frequency Questionnaire | Harvard FFQ | Adapted | No | Yes | No | No | No | N/A |
| Trauchburg et al. 2023 | McCullough et al. 2016 | Block Food Frequencies Questionnaire | Block FFQ | Adapted | No | Yes | No | No | No | N/A |
| Trauchburg et al. 2023 | Meyerhardt et al. 2007 | Semi Quantitative Food Frequency Questionnaire | SFFQ | Validated | No | Yes | No | No | No | N/A |
| Trauchburg et al. 2023 | Ollberding et al. 2013 | Health Habits and History Questionnaire | HHHQ | Adapted | No | Yes | No | No | No | N/A |
| Trauchburg et al. 2023 | Park et al. 2022b | Quantitative Food Frequency Questionnaire | QFFQ | Validated | No | Yes | No | No | No | N/A |
| Trauchburg et al. 2023 | Pelser et al. 2014 | Healthy Eating Index-2005 | HEI-2005 | Validated | No | Yes | No | No | No | N/A |
| Trauchburg et al. 2023 | Ratjen et al. 2017 | Food Frequencies Questionnaire | FFQ | Validated | No | Yes | No | No | No | N/A |
| Trauchburg et al. 2023 | Ratjen et al. 2017 | Healthy Nordic Food Index | HNFI | Validated | No | Yes | No | No | No | N/A |
| Trauchburg et al. 2023 | Ratjen et al. 2017 | Modified Mediterranean Diet Score | MMDS | Validated | No | Yes | No | No | No | N/A |
| Trauchburg et al. 2023 | Ratjen et al. 2021 | Food Frequencies Questionnaire | FFQ | NR | No | Yes | No | No | No | N/A |
| Trauchburg et al. 2023 | Sharma et al. 2018 | Food Frequencies Questionnaire | FFQ | Adapted | No | Yes | No | No | No | N/A |
| Trauchburg et al. 2023 | Sharma et al. 2018 | Alternate Mediterranean Diet score | aMED | Validated | No | Yes | No | No | No | N/A |
| Trauchburg et al. 2023 | Song et al. 2021 | Food Frequencies Questionnaire | FFQ | Validated | No | Yes | No | No | No | N/A |
| Trauchburg et al. 2023 | Sun et al. 2018 | Healthy Eating Index 2010 | HEI-2010 | Validated | No | Yes | No | No | No | N/A |
| Trauchburg et al. 2023 | Sun et al. 2018 | Women’s Health Initiative Food Frequencies Questionnaire | WHI FFQ | Validated | No | Yes | No | No | No | N/A |
| Trauchburg et al. 2023 | Thomson et al. 2014 | Healthy Eating Index-2005 | HEI-2005 | Validated | No | Yes | No | No | No | N/A |
| Trauchburg et al. 2023 | Thomson et al. 2014 | Women’s Health Initiative Food Frequencies Questionnaire | WHI FFQ | Validated | No | Yes | No | No | No | N/A |
| Trauchburg et al. 2023 | van Blarigan et al. 2020 | Food Frequencies Questionnaire | FFQ | Validated | No | Yes | No | No | No | N/A |
| Trauchburg et al. 2023 | van Zutphen et al. 2021 | Food Frequencies Questionnaire | FFQ | NR | No | Yes | No | No | No | N/A |
| van Gestel et al. 2022 [76] | Bousquet-Dion et al. 2018 | Diary | NR | NR | No | No | Yes | No | No | N/A |
| van Gestel et al. 2022 | Bousquet-Dion et al. 2018 | Diary | NR | NR | No | Yes | No | No | No | N/A |
| van Gestel et al. 2022 | Gillis et al. 2014 | Diary | NR | NR | No | Yes | No | No | No | N/A |
| van Gestel et al. 2022 | Gillis et al. 2014 | Diary | NR | NR | No | No | Yes | No | No | N/A |
| van Gestel et al. 2022 | Kahn et al. 2007 | Questionnaire | NR | NR | Yes | No | No | No | No | N/A |
| van Gestel et al. 2022 | Liu et al. 2019 | Diary | NR | NR | No | No | Yes | No | No | N/A |
| van Gestel et al. 2022 | Minnella et al. 2018 | Diary | NR | NR | No | No | Yes | No | No | N/A |
| van Gestel et al. 2022 | Minnella et al. 2019 | Diary | NR | NR | No | No | Yes | No | No | N/A |
| Van Liew et al. 2014 [77] | Kyvernitakis et al. 2014 | Questionnaire | NR | NR | Yes | No | No | No | No | N/A |
| Van Liew et al. 2014 | Lash et al. 2006 | Questionnaire | NR | NR | Yes | No | No | No | No | N/A |
| Van Liew et al. 2014 | Liu et al. 2013 | Questionnaire | NR | NR | Yes | No | No | No | No | N/A |
| Van Liew et al. 2014 | Xu et al. 2012 | Questionnaire | NR | NR | Yes | No | No | No | No | N/A |
| Verbrugghe et al. 2013 [78] | Atkins & Fallowfield 2006 | Questionnaire | NR | NR | Yes | No | No | No | No | N/A |
| Verbrugghe et al. 2013 | Barchitta et al. 2020 | Mediterranean Diet Adherence Score | MEDAS | Validated | No | Yes | No | No | No | N/A |
| Verbrugghe et al. 2013 | Di Maso et al. 2020 | Food Frequencies Questionnaire | FFQ | Validated | No | Yes | No | No | No | N/A |
| Verbrugghe et al. 2013 | Di Maso et al. 2020 | Mediterranean Diet Score | MDS | Validated | No | Yes | No | No | No | N/A |
| Verbrugghe et al. 2013 | Eliasson et al. 2011 | Questionnaire | NR | NR | Yes | No | No | No | No | N/A |
| Verbrugghe et al. 2013 | Grunfeld et al. 2005 | Beliefs About Medicines Questionnaire | BMQ | Validated | Yes | No | No | No | No | N/A |
| Verbrugghe et al. 2013 | Grunfeld et al. 2005 | Medication Adherence Rating Scale-5 | MARS-5 | Validated | Yes | No | No | No | No | N/A |
| Verbrugghe et al. 2013 | Grunfeld et al. 2005 | Single Item Measure | NR | NR | Yes | No | No | No | No | N/A |
| Verbrugghe et al. 2013 | Heidari et al. 2020 | Dietary Approaches to Stop Hypertension | DASH | Validated | No | Yes | No | No | No | N/A |
| Verbrugghe et al. 2013 | Kahn et al. 2007 | Questionnaire | NR | NR | Yes | No | No | No | No | N/A |
| Verbrugghe et al. 2013 | Kirk & Hudis 2008 | Questionnaire | NR | Not Validated | Yes | No | No | No | No | N/A |
| Verbrugghe et al. 2013 | Lash et al. 2006 | Questionnaire | NR | NR | Yes | No | No | No | No | N/A |
| Verbrugghe et al. 2013 | Lebovits et al. 1990 | Questionnaire | NR | NR | Yes | No | No | No | No | N/A |
| Verbrugghe et al. 2013 | Marques & Pierin 2008 | Morisky Medication Adherence Scale-4 | MMAS-4 | Validated | Yes | No | No | No | No | N/A |
| Verbrugghe et al. 2013 | Noens et al. 2009 | Basel Assessment of Adherence Scale | BAAS | Validated | Yes | No | No | No | No | N/A |
| Verbrugghe et al. 2013 | Noens et al. 2009 | Visual Analog Scale | VAS | Validated | Yes | No | No | No | No | N/A |
| Virani et al. 2024 [79] | Montagnese et al. 2020 | Mediterranean Diet Adherence Score | MEDAS | Validated | No | Yes | No | No | No | N/A |
| Virani et al. 2024 | Negrati et al. 2021 | Mediterranean Diet Score | MDS | Validated | No | Yes | No | No | No | N/A |
| Virani et al. 2024 | Rein et al. 2022 | Mediterranean Diet Score | MDS | Validated | No | Yes | No | No | No | N/A |
| Visintini et al. 2023 [80] | Gresch et al. 2017 | Basel Assessment of Adherence to Immunosuppressive Medication Scale | BAASIS | Validated | Yes | No | No | No | No | N/A |
| Visintini et al. 2023 | Ice et al. 2020 | Immunosuppressant Therapy Adherence Scale | ITAS | Validated | Yes | No | No | No | No | N/A |
| Visintini et al. 2023 | Ice et al. 2020 | Morisky Medication Adherence Scale-8 | MMAS-8 | Validated | Yes | No | No | No | No | N/A |
| Visintini et al. 2023 | Scherer et al. 2021 | Medication Experience Scale for Immunosuppressants | MESI | Validated | Yes | No | No | No | No | N/A |
| Visintini et al. 2023 | Zanetti et al. 2022 | Brief Medication Questionnaire | BMQ | Validated | Yes | No | No | No | No | N/A |
| Visintini et al. 2023 | Belaiche et al. 2021 | Compliance Evaluation Test | CET | Adapted | Yes | No | No | No | No | N/A |
| Visintini et al. 2023 | Belaiche et al. 2021 | Compliance Evaluation Test | CET | Validated | Yes | No | No | No | No | N/A |
| Visintini et al. 2023 | Lehrer et al. 2018 | Morisky Medication Adherence Scale-8 | MMAS-8 | Validated | Yes | No | No | No | No | N/A |
| Wang et al. 2023 [81] | Bauer et al. 2005 | Diary | NR | NR | No | Yes | No | No | No | N/A |
| Wang et al. 2023 | Chu et al. 2020b | Diary | NR | NR | Yes | No | No | No | No | N/A |
| Wang et al. 2023 | Grass et al. 2015 | Diary | NR | NR | No | Yes | No | No | No | N/A |
| Wang et al. 2023 | Hopanci et al. 2017 | Diary | NR | NR | No | Yes | No | No | No | N/A |
| Wang et al. 2023 | Kobayashi et al. 2017 | Questionnaire | NR | NR | No | Yes | No | No | No | N/A |
| Wang et al. 2023 | Lidoriki et al. 2020 | Diary | NR | NR | No | Yes | No | No | No | N/A |
| Wang et al. 2023 | Lidoriki et al. 2020 | Questionnaire | NR | NR | No | Yes | No | No | No | N/A |
| Wang et al. 2023 | McGough et al. 2006 | Diary | NR | NR | No | Yes | No | No | No | N/A |
| Wang et al. 2023 | Qiao et al. 2018 | Questionnaire | NR | NR | No | Yes | No | No | No | N/A |
| Wang et al. 2023 | Read et al. 2007 | Diary | NR | NR | No | Yes | No | No | No | N/A |
| Wang et al. 2023 | Sandmael et al. 2017 | Questionnaire | NR | NR | No | Yes | No | No | No | N/A |
| Wang et al. 2023 | Sandmael et al. 2017 | Diary | NR | NR | No | No | Yes | No | No | N/A |
| Wang et al. 2023 | Schmidt et al. 2020 | Diary | NR | NR | No | Yes | No | No | No | N/A |
| Waseem et al. 2022 [82] | Berry et al. 2015 | Morisky Medication Adherence Scale-8 | MMAS-8 | Adapted | Yes | No | No | No | No | N/A |
| Waseem et al. 2022 | Byrne et al. 2018 | Leuven Questionnaire | NR | Adapted | Yes | No | No | No | No | N/A |
| Waseem et al. 2022 | Gönderen Çakmak & Kapucu 2021 | Oral Chemotherapy Adherence Scale | OCAS | Validated | Yes | No | No | No | No | N/A |
| Waseem et al. 2022 | Gönderen Çakmak & Kapucu 2021 | Medication Adherence Self-Efficacy Scale | MASES | Validated | Yes | No | No | No | No | N/A |
| Waseem et al. 2022 | Krikorian et al. 2019 | Beliefs About Medicines Questionnaire | BMQ | Validated | Yes | No | No | No | No | N/A |
| Waseem et al. 2022 | Lin et al. 2020 | Questionnaire | NR | NR | Yes | No | No | No | No | N/A |
| Waseem et al. 2022 | Morgan et al. 2018 | Chew et al.’s health literacy questionnaire | NR | Adapted | Yes | No | No | No | No | N/A |
| Waseem et al. 2022 | Suttmann et al. 2020 | Morisky Medication Adherence Scale-4 | MMAS-4 | Validated | Yes | No | No | No | No | N/A |
| Waseem et al. 2022 | Zerbit et al. 2020 | Diary | NR | NR | Yes | No | No | No | No | N/A |
| Waseem et al. 2022 | Ziller et al. 2013 | Questionnaire | NR | Not Validated | Yes | No | No | No | No | N/A |
| Watts et al. 2024 [83] | Brahmbhatt 2020 | Diary | NR | NR | No | No | Yes | No | No | N/A |
| Watts et al. 2024 | Burden 2017 | Diary | NR | NR | No | Yes | No | No | No | N/A |
| Watts et al. 2024 | Ferreira 2021 | Diary | NR | NR | No | Yes | Yes | No | No | N/A |
| Watts et al. 2024 | Machado 2023 | Diary | NR | NR | No | No | Yes | No | No | N/A |
| Watts et al. 2024 | Minnella et al. 2019 | Diary | NR | NR | No | No | Yes | No | No | N/A |
| Watts et al. 2024 | Moorthy et al. 2023 | International Physical Activity Questionnaire | IPAQ | Validated | No | No | Yes | No | No | N/A |
| Watts et al. 2024 | Naito et al. 2019 | Diary | NR | NR | No | Yes | Yes | No | No | N/A |
| Watts et al. 2024 | Piraux et al. 2020 | Diary | NR | NR | No | No | Yes | No | No | N/A |
| Watts et al. 2024 | Rupnik et al. 2020 | Diary | NR | NR | No | Yes | Yes | No | No | N/A |
| Watts et al. 2024 | Santa Mina et al. 2018 | Diary | NR | NR | No | No | Yes | No | No | N/A |
| Watts et al. 2024 | Steffens et al. 2021 | Diary | NR | NR | No | No | Yes | No | No | N/A |
| Yang et al. 2023 [84] | Ali et al. 2017 | Morisky Medication Adherence Scale-4 | MMAS-4 | Validated | Yes | No | No | No | No | N/A |
| Yang et al. 2023 | Arriola et al. 2014 | Drug Attittude Inventory | DAI | Validated | Yes | No | No | No | No | N/A |
| Yang et al. 2023 | Arriola et al. 2014 | Medication Adherence Rating Scale-10 | MARS-10 | Validated | Yes | No | No | No | No | N/A |
| Yang et al. 2023 | Bhatta et al. 2013 | Single Item Measure | NR | Validated | Yes | No | No | No | No | N/A |
| Yang et al. 2023 | Brett et al. 2018 | Medication Adherence Rating Scale-5 | MARS-5 | Validated | Yes | No | No | No | No | N/A |
| Yang et al. 2023 | Bright et al. 2016 | Morisky Medication Adherence Scale-8 | MMAS-8 | Adapted | Yes | No | No | No | No | N/A |
| Yang et al. 2023 | Corter et al. 2018 | Beliefs About Medicines Questionnaire | BMQ | Adapted | Yes | No | No | No | No | N/A |
| Yang et al. 2023 | Font et al. 2012 | Single Item Measure | NR | Not Validated | Yes | No | No | No | No | N/A |
| Yang et al. 2023 | Friese et al. 2013 | Single Item Measure | NR | NR | Yes | No | No | No | No | N/A |
| Yang et al. 2023 | Graetz et al. 2018 | Medication Adherence Reasons | MARS-1 | Validated | Yes | No | No | No | No | N/A |
| Yang et al. 2023 | Graetz et al. 2018 | Morisky Medication Adherence Scale-4 | MMAS-4 | Validated | Yes | No | No | No | No | N/A |
| Yang et al. 2023 | Grunfeld et al. 2005 | Beliefs About Medicines Questionnaire | BMQ | Validated | Yes | No | No | No | No | N/A |
| Yang et al. 2023 | Grunfeld et al. 2005 | Medication Adherence Rating Scale-5 | MARS-5 | Validated | Yes | No | No | No | No | N/A |
| Yang et al. 2023 | Grunfeld et al. 2005 | Single Item Measure | NR | NR | Yes | No | No | No | No | N/A |
| Yang et al. 2023 | Hadji et al. 2013 | Morisky Medication Adherence Scale-4 | MMAS-4 | Adapted | Yes | No | No | No | No | N/A |
| Yang et al. 2023 | Hadji et al. 2014 | Questionnaire | NR | NR | Yes | No | No | No | No | N/A |
| Yang et al. 2023 | Haji-Hersi et al. 2021 | Morisky Medication Adherence Scale-8 | MMAS-8 | Adapted | Yes | No | No | No | No | N/A |
| Yang et al. 2023 | Heisig et al. 2015 | Questionnaire | NR | Validated | Yes | No | No | No | No | N/A |
| Yang et al. 2023 | Hurtado-de-Mendoza et al. 2019b | Morisky Medication Adherence Scale-8 | MMAS-8 | Validated | Yes | No | No | No | No | N/A |
| Yang et al. 2023 | Kahn et al. 2007 | Questionnaire | NR | NR | Yes | No | No | No | No | N/A |
| Yang et al. 2023 | Karmakar et al. 2017 | Morisky Medication Adherence Scale-8 | MMAS-8 | Validated | Yes | No | No | No | No | N/A |
| Yang et al. 2023 | Kimmick et al. 2015 | Morisky Medication Adherence Scale-8 | MMAS-8 | Validated | Yes | No | No | No | No | N/A |
| Yang et al. 2023 | Kyvernitakis et al. 2014 | Questionnaire | NR | NR | Yes | No | No | No | No | N/A |
| Yang et al. 2023 | lacorossi et al. 2016 | Morisky Medication Adherence Scale-8 | MMAS-8 | Validated | Yes | No | No | No | No | N/A |
| Yang et al. 2023 | Lee & Min 2018 | Morisky Medication Adherence Scale-8 | MMAS-8 | Validated | Yes | No | No | No | No | N/A |
| Yang et al. 2023 | Livaudais et al. 2012a | Single Item Measure | NR | NR | Yes | No | No | No | No | N/A |
| Yang et al. 2023 | Markopoulos et al. 2015 | Questionnaire | NR | NR | Yes | No | No | No | No | N/A |
| Yang et al. 2023 | Markovitz et al. 2017 | Morisky Medication Adherence Scale-4 | MMAS-4 | Validated | Yes | No | No | No | No | N/A |
| Yang et al. 2023 | Martinez-Cannon 2021 | Questionnaire | NR | NR | Yes | No | No | No | No | N/A |
| Yang et al. 2023 | Moon et al. 2017 | Medication Adherence Rating Scale-5 | MARS-5 | Validated | Yes | No | No | No | No | N/A |
| Yang et al. 2023 | Moon et al. 2019a | Medication Adherence Rating Scale-5 | MARS-5 | Validated | Yes | No | No | No | No | N/A |
| Yang et al. 2023 | Nestoriuc et al. 2016 | Questionnaire | NR | Validated | Yes | No | No | No | No | N/A |
| Yang et al. 2023 | Neven et al. 2014 | Questionnaire | NR | NR | Yes | No | No | No | No | N/A |
| Yang et al. 2023 | Pan et al. 2018 | Single Item Measure | NR | Validated | Yes | No | No | No | No | N/A |
| Yang et al. 2023 | Philipovskiy et al. 2020 | Morisky Medication Adherence Scale-8 | MMAS-8 | Validated | Yes | No | No | No | No | N/A |
| Yang et al. 2023 | Pinheiro et al. 2017 | Morisky Medication Adherence Scale | MMAS | Adapted | Yes | No | No | No | No | N/A |
| Yang et al. 2023 | Pinheiro et al. 2017 | Questionnaire | NR | NR | Yes | No | No | No | No | N/A |
| Yang et al. 2023 | Pourcelot et al. 2018 | Morisky Medication Adherence Scale-4 | MMAS-4 | Validated | Yes | No | No | No | No | N/A |
| Yang et al. 2023 | Quinn et al. 2016 | Morisky Medication Adherence Scale-8 | MMAS-8 | Validated | Yes | No | No | No | No | N/A |
| Yang et al. 2023 | Schover et al. 2014 | 3-item Adherence Estimator | NR | Validated | Yes | No | No | No | No | N/A |
| Yang et al. 2023 | Shinn et al. 2019 | Questionnaire | NR | Not Validated | Yes | No | No | No | No | N/A |
| Yang et al. 2023 | Spencer et al. 2020 | Questionnaire | NR | NR | Yes | No | No | No | No | N/A |
| Yang et al. 2023 | Stahlschmidt et al. 2019 | Morisky Medication Adherence Scale-4 | MMAS-4 | Validated | Yes | No | No | No | No | N/A |
| Yang et al. 2023 | Stanton et al. 2014 | Morisky Medication Adherence Scale-4 | MMAS-4 | Adapted | Yes | No | No | No | No | N/A |
| Yang et al. 2023 | Tan et al. 2020 | Simplified Medication Adherence Questionnaire | SMAQ | Validated | Yes | No | No | No | No | N/A |
| Yang et al. 2023 | Tan et al. 2021 | Simplified Medication Adherence Questionnaire | SMAQ | Validated | Yes | No | No | No | No | N/A |
| Yang et al. 2023 | Tinari et al. 2015 | Questionnaire | NR | NR | Yes | No | No | No | No | N/A |
| Yang et al. 2023 | Walker et al. 2016 | Morisky Medication Adherence Scale | MMAS | Adapted | Yes | No | No | No | No | N/A |
| Yang et al. 2023 | Wassermann et al. 2019 | Morisky Medication Adherence Scale | MMAS | Adapted | Yes | No | No | No | No | N/A |
| Yang et al. 2023 | Wheeler et al. 2019 | Questionnaire | NR | Not Validated | Yes | No | No | No | No | N/A |
| Yang et al. 2023 | Wouters et al. 2014 | Medication Adherence Rating Scale-5 | MARS-5 | Validated | Yes | No | No | No | No | N/A |
| Yang et al. 2023 | Wouters et al. 2014 | Morisky Medication Adherence Scale-8 | MMAS-8 | Validated | Yes | No | No | No | No | N/A |
| Yang et al. 2023 | Wouters et al. 2014 | Questionnaire | NR | NR | Yes | No | No | No | No | N/A |
| Yang et al. 2023 | Yusufov et al. 2021 | Questionnaire | NR | NR | Yes | No | No | No | No | N/A |
| Yang et al. 2023 | Ziller et al. 2009 | Questionnaire | NR | Validated | Yes | No | No | No | No | N/A |
| Yang et al. 2023 | Ziller et al. 2013 | Questionnaire | NR | Not Validated | Yes | No | No | No | No | N/A |
| Yussof et al. 2022 [85] | Bhatta et al. 2013 | Single Item Measure | NR | Validated | Yes | No | No | No | No | N/A |
| Yussof et al. 2022 | Font et al. 2012 | Single Item Measure | NR | NR | Yes | No | No | No | No | N/A |
| Yussof et al. 2022 | Hagen et al. 2019 | Morisky Medication Adherence Scale-8 | MMAS-8 | Adapted | Yes | No | No | No | No | N/A |
| Zerillo et al. 2018 [86] | Ramesh et al. 2015 | Beliefs About Medicines Questionnaire | BMQ | Validated | Yes | No | No | No | No | N/A |
| Zerillo et al. 2018 | Ramesh et al. 2015 | Medication Adherence Rating Scale | MARS-1 | Validated | Yes | No | No | No | No | N/A |
| Zerillo et al. 2018 | Schneider et al. 2014 | Questionnaire | NR | Not Validated | Yes | No | No | No | No | N/A |
| Zerillo et al. 2018 | Sitaresmi et al. 2013 | Diary | NR | NR | Yes | No | No | No | No | N/A |
| Zhong et al. 2020 [87] | Bamia et al. 2013 | Modified Mediterranean Diet Score | MMDS | Validated | No | Yes | No | No | No | N/A |
| Zhong et al. 2020 | Cheng et al. 2018 | Mediterranean Diet Pattern Score | NR | Validated | No | Yes | No | No | No | N/A |
| Zhong et al. 2020 | Fung et al. 2014 | Alternate Healthy Eating Index 2010 | AHEI-2010 | Validated | No | Yes | No | No | No | N/A |
| Zhong et al. 2020 | Fung et al. 2014 | Alternate Mediterranean Diet score | aMED | Validated | No | Yes | No | No | No | N/A |
| Zhong et al. 2020 | Fung et al. 2014 | Dietary Approaches to Stop Hypertension | DASH | Validated | No | Yes | No | No | No | N/A |
| Zhong et al. 2020 | Fung et al. 2014 | Semi Quantitative Food Frequency Questionnaire | SFFQ | NR | No | Yes | No | No | No | N/A |
| Zhong et al. 2020 | Jacobs et al. 2016 | Alternate Healthy Eating Index 2010 | AHEI-2010 | Validated | No | Yes | No | No | No | N/A |
| Zhong et al. 2020 | Jacobs et al. 2016 | Alternate Mediterranean Diet score | aMED | Validated | No | Yes | No | No | No | N/A |
| Zhong et al. 2020 | Jacobs et al. 2016 | Dietary Approaches to Stop Hypertension | DASH | Validated | No | Yes | No | No | No | N/A |
| Zhong et al. 2020 | Jacobs et al. 2016 | Healthy Eating Index 2010 | HEI 2010 | Validated | No | Yes | No | No | No | N/A |
| Zhong et al. 2020 | Jones et al. 2017 | Modified Mediterranean Diet Score | MMDS | Validated | No | Yes | No | No | No | N/A |
| Zhong et al. 2020 | Lavalette et al. 2018 | Adherence Score to Mediterranean Diet | MEDI-LITE | Validated | No | Yes | No | No | No | N/A |
| Zhong et al. 2020 | Park et al. 2017 | Alternate Mediterranean Diet score | aMED | Validated | No | Yes | No | No | No | N/A |
| Zhong et al. 2020 | Petimar et al. 2018 | Alternate Mediterranean Diet score | aMED | Validated | No | Yes | No | No | No | N/A |
| Zhong et al. 2020 | Ratjen et al. 2017 | Food Frequencies Questionnaire | FFQ | Validated | No | Yes | No | No | No | N/A |
| Zhong et al. 2020 | Ratjen et al. 2017 | Healthy Nordic Food Index | HNFI | Validated | No | Yes | No | No | No | N/A |
| Zhong et al. 2020 | Ratjen et al. 2017 | Modified Mediterranean Diet Score | MMDS | Validated | No | Yes | No | No | No | N/A |
| Zhong et al. 2020 | Reedy et al. 2008 | Modified Mediterranean Diet Score | MMDS | Validated | No | Yes | No | No | No | N/A |
| Zhong et al. 2020 | Schulpen et al. 2020 | Alternate Mediterranean Diet score | aMED | Validated | No | Yes | No | No | No | N/A |
| Zhong et al. 2020 | Sharma et al. 2018 | Alternate Mediterranean Diet score | aMED | Validated | No | Yes | No | No | No | N/A |
| Zhong et al. 2020 | Sharma et al. 2018 | Food Frequencies Questionnaire | FFQ | Adapted | No | Yes | No | No | No | N/A |
| Zhong et al. 2020 | Vargas et al. 2016 | Alternate Mediterranean Diet score | aMED | Validated | No | Yes | No | No | No | N/A |
| Zou et al. 2023 [88] | Zhang et al. 2021 | Morisky Medication Adherence Scale-4 | MMAS-4 | Validated | Yes | No | No | No | No | N/A |
| Zullig et al. 2023 [89] | Birand et al. 2019 | Beliefs About Medicines Questionnaire | BMQ | Validated | Yes | No | No | No | No | N/A |
| Zullig et al. 2023 | Birand et al. 2019 | Morisky Medication Adherence Scale-4 | MMAS-4 | Validated | Yes | No | No | No | No | N/A |
| Zullig et al. 2023 | Drzayich Antol et al. 2018 | Morisky Medication Adherence Scale-8 | MMAS-8 | Validated | Yes | No | No | No | No | N/A |
| Zullig et al. 2023 | James et al. 2018 | Morisky Medication Adherence Scale-8 | MMAS-8 | Validated | Yes | No | No | No | No | N/A |
| Zullig et al. 2023 | Shin et al. 2012 | Single Item Measure | NR | Not Validated | Yes | No | No | No | No | N/A |

*Note. NR = Not Reported, N/A = Not Applicable*

References

[1]	Abboud M, AlAnouti F, Georgaki E, Papandreou D. Effect of Ketogenic Diet on Quality of Life in Adults with Chronic Disease: A Systematic Review of Randomized Controlled Trials. Nutrients 2021; 13(12)
[https://doi.org/10.3390/nu13124463][PMID: 34960015]

[2]	Agirre-Elordui S, Fernández-Landa J, Olasagasti-Ibargoien J, Castañeda-Babarro A. Physical activity maintenance in colorectal cancer survivors after an exercise intervention applying behaviour change techniques: a systematic review and meta-analysis. J Cancer Surviv 2024
[https://doi.org/10.1007/s11764-024-01654-8][PMID: 39098882]

[3]	Ahmed S, Loiselle CG. Patient Adherence to Oral Anticancer Agents: A Mapping Review of Supportive Interventions. Curr Oncol 2023; 30(12): 10224–36
[https://doi.org/10.3390/curroncol30120744][PMID: 38132378]

[4]	Alves AR, Lima WG, Nagai MM, Rodrigues JPV, Ayres LR. Adherence and/or discontinuation of imatinib mesylate in patients with chronic myeloid leukemia. Braz. J. Pharm. Sci. 2016; 52(4): 581–9
[https://doi.org/10.1590/S1984-82502016000400001]

[5]	Atkinson TM, Rodríguez VM, Gordon M*, et al.* The Association Between Patient-Reported and Objective Oral Anticancer Medication Adherence Measures: A Systematic Review. Oncol Nurs Forum 2016; 43(5): 576–82
[https://doi.org/10.1188/16.ONF.576-582][PMID: 27541550]

[6]	Barnes KA, Ball LE, Galvão DA, Newton RU, Chambers SK. Nutrition care guidelines for men with prostate cancer undergoing androgen deprivation therapy: do we have enough evidence? Prostate Cancer Prostatic Dis 2019; 22(2): 221–34
[https://doi.org/10.1038/s41391-018-0099-9][PMID: 30279584]

[7]	Baydoun M, Moran C, McLennan A, Piedalue K-AL, Oberoi D, Carlson LE. Mindfulness-Based Interventions in Cancer Survivors: A Systematic Review of Participants' Adherence to Home Practice. Patient Prefer Adherence 2021; 15: 1225–42
[https://doi.org/10.2147/PPA.S267064][PMID: 34135575]

[8]	Bright EE, Finkelstein LB, Nealis MS*, et al.* A Systematic Review and Meta-Analysis of Interventions to Promote Adjuvant Endocrine Therapy Adherence Among Breast Cancer Survivors. J Clin Oncol 2023; 41(28): 4548–61
[https://doi.org/10.1200/JCO.23.00697][PMID: 37531593]

[9]	Bruns ERJ, van den Heuvel B, Buskens CJ*, et al.* The effects of physical prehabilitation in elderly patients undergoing colorectal surgery: a systematic review. Colorectal Dis 2016; 18(8): O267-77
[https://doi.org/10.1111/codi.13429][PMID: 27332897]

[10]	Burke D, Brown M, O'Neill C*, et al.* The effect of lifestyle interventions on sarcopenia in advanced colorectal cancer: A systematic review. J Geriatr Oncol 2025; 16(1): 102143
[https://doi.org/10.1016/j.jgo.2024.102143][PMID: 39472241]

[11]	Cahir C, Guinan E, Dombrowski SU, Sharp L, Bennett K. Identifying the determinants of adjuvant hormonal therapy medication taking behaviour in women with stages I-III breast cancer: A systematic review and meta-analysis. Patient Educ Couns 2015
[https://doi.org/10.1016/j.pec.2015.05.013][PMID: 26054455]

[12]	Carr E, Rosengarten L. Teenagers and Young Adults with Cancer: An Exploration of Factors Contributing to Treatment Adherence. J Pediatr Oncol Nurs 2021; 38(3): 190–204
[https://doi.org/10.1177/1043454221992302][PMID: 33769898]

[13]	Charters E, Ricketts V, Sharman AR, Clark J. Systematic review of adherence to swallow and trismus exercises during radiation therapy for head and neck cancer. Head Neck 2024; 46(9): 2348–62
[https://doi.org/10.1002/hed.27849][PMID: 38895913]

[14]	Chen J, Jia L, Li Y*, et al.* Factors influencing adherence to healthy eating behaviors among adult colorectal cancer survivors: a systematic mixed method review. Support Care Cancer 2022; 31(1): 88
[https://doi.org/10.1007/s00520-022-07475-8][PMID: 36574042]

[15]	Chen G, Leary S, Niu J, Perry R, Papadaki A. The Role of the Mediterranean Diet in Breast Cancer Survivorship: A Systematic Review and Meta-Analysis of Observational Studies and Randomised Controlled Trials. Nutrients 2023; 15(9)
[https://doi.org/10.3390/nu15092099][PMID: 37432242]

[16]	Cheung C, Boocock E, Grande AJ, Maddocks M. Exercise-based interventions for cancer cachexia: A systematic review of randomised and non-randomised controlled trials. Asia Pac J Oncol Nurs 2023; 10(Suppl 1): 100335
[https://doi.org/10.1016/j.apjon.2023.100335][PMID: 38197041]

[17]	Chlebowski RT, Geller ML. Adherence to endocrine therapy for breast cancer. Oncology 2006; 71(1-2): 1–9
[https://doi.org/10.1159/000100444][PMID: 17344666]

[18]	Chow SM, Tan BK. Effectiveness of mHealth apps on adherence and symptoms to oral anticancer medications: a systematic review and meta-analysis. Support Care Cancer 2024; 32(7): 426
[https://doi.org/10.1007/s00520-024-08635-8][PMID: 38864924]

[19]	Claros MP, Messa CVM, García-Perdomo HA. Adherence to oral pharmacological treatment in cancer patients: Systematic review. Oncol Rev 2019; 13(1): 402
[https://doi.org/10.4081/oncol.2019.402][PMID: 31044026]

[20]	Deng T, Song J, Tuo J*, et al.* Incidence and risk factors of pulmonary complications after lung cancer surgery: A systematic review and meta-analysis. Heliyon 2024; 10(12): e32821
[https://doi.org/10.1016/j.heliyon.2024.e32821][PMID: 38975138]

[21]	Eliasson L. Treatment adherence in chronic myeloid leukemia: a systematic review of the literature. Clinical Practice 2012; 9(1): 87–100
[https://doi.org/10.2217/CPR.11.127]

[22]	Finitsis DJ, Vose BA, Mahalak JG, Salner AL. Interventions to promote adherence to endocrine therapy among breast cancer survivors: A meta-analysis. Psychooncology 2018; 28(2): 255–63
[https://doi.org/10.1002/pon.4959][PMID: 30511789]

[23]	Fleming L, Agnew S, Peddie N, Crawford M, Dixon D, MacPherson I. The impact of medication side effects on adherence and persistence to hormone therapy in breast cancer survivors: A quantitative systematic review. Breast 2022; 64: 63–84
[https://doi.org/10.1016/j.breast.2022.04.010][PMID: 35609380]

[24]	Ganna S, Rahimi S, Lu A, Laborde K, Trivedi M. Interventions to improve oral endocrine therapy adherence in breast cancer patients. J Cancer Surviv 2025; 19(3): 930–9
[https://doi.org/10.1007/s11764-023-01513-y][PMID: 38233637]

[25]	Greer JA, Amoyal N, Nisotel L*, et al.* A Systematic Review of Adherence to Oral Antineoplastic Therapies. Oncologist 2016; 21(3): 354–76
[https://doi.org/10.1634/theoncologist.2015-0405][PMID: 26921292]

[26]	Haddad NC, Soares Brollo LC, Pinho Oliveira MA, Bernardo-Filho M. Diagnostic Methods for Vaginal Stenosis and Compliance to Vaginal Dilator Use: A Systematic Review. J Sex Med 2021; 18(3): 493–514
[https://doi.org/10.1016/j.jsxm.2020.12.013][PMID: 33526400]

[27]	Hajjar M, Rezazadeh A. Recommended Food Score and Healthy Nordic Food Index in Cancer: A Systematic Review. Nutr Cancer 2021; 73(3): 369–75
[https://doi.org/10.1080/01635581.2020.1761406][PMID: 32400196]

[28]	Haynes DA, Vanison CC, Gillespie MB. The Impact of Dental Care in Head and Neck Cancer Outcomes: A Systematic Review and Meta-Analysis. Laryngoscope 2021; 132(1): 45–52
[https://doi.org/10.1002/lary.29494][PMID: 33635587]

[29]	Heiney SP, Parker PD, Felder TM, Adams SA, Omofuma OO, Hulett JM. A systematic review of interventions to improve adherence to endocrine therapy. Breast Cancer Res Treat 2018; 173(3): 499–510
[https://doi.org/10.1007/s10549-018-5012-7][PMID: 30387003]

[30]	Heiney SP, Sorrell M, Sheng J*, et al.* Interventions to Improve Adherence to Tyrosine Kinase Inhibitors in Chronic Myeloid Leukemia: A Systematic Review. Am J Clin Oncol 2021; 44(6): 291–8
[https://doi.org/10.1097/COC.0000000000000818][PMID: 33867480]

[31]	Horne R, Chapman SCE, Parham R, Freemantle N, Forbes A, Cooper V. Understanding patients' adherence-related beliefs about medicines prescribed for long-term conditions: a meta-analytic review of the Necessity-Concerns Framework. PLoS One 2013; 8(12): e80633
[https://doi.org/10.1371/journal.pone.0080633][PMID: 24312488]

[32]	Huang W-C, Chen C-Y, Lin S-J, Chang C-S. Medication adherence to oral anticancer drugs: systematic review. Expert Rev Anticancer Ther 2016; 16(4): 423–32
[https://doi.org/10.1586/14737140.2016.1159515][PMID: 26935964]

[33]	Hurtado-de-Mendoza A, Cabling ML, Lobo T, Dash C, Sheppard VB. Behavioral Interventions to Enhance Adherence to Hormone Therapy in Breast Cancer Survivors: A Systematic Literature Review. Clin Breast Cancer 2016; 16(4): 247-255.e3
[https://doi.org/10.1016/j.clbc.2016.03.006][PMID: 27133733]

[34]	Kaptein AA, Schoones JW, van der Meer PB*, et al.* Psychosocial determinants of adherence with oral anticancer treatment: 'we don't need no education'. Acta Oncol 2021; 60(1): 87–95
[https://doi.org/10.1080/0284186X.2020.1843190][PMID: 33151764]

[35]	Kavookjian J, Wittayanukorn S. Interventions for adherence with oral chemotherapy in hematological malignancies: A systematic review. Res Social Adm Pharm 2015; 11(3): 303–14
[https://doi.org/10.1016/j.sapharm.2014.08.006][PMID: 25262600]

[36]	Kearney N, Connolly D, Begic S, Mockler D, Guinan E. Feasibility metrics of exercise interventions during chemotherapy: A systematic review. Crit Rev Oncol Hematol 2024; 195: 104272
[https://doi.org/10.1016/j.critrevonc.2024.104272][PMID: 38272152]

[37]	Krekeler BN, Broadfoot CK, Johnson S, Connor NP, Rogus-Pulia N. Patient Adherence to Dysphagia Recommendations: A Systematic Review. Dysphagia 2018; 33(2): 173–84
[https://doi.org/10.1007/s00455-017-9852-9][PMID: 28965240]

[38]	Lafata JE, Nguyen B, Staresinic C, Johnson M, Gratie D, Muluneh B. Interpersonal communication-, education- and counselling-based interventions to support adherence to oral anticancer therapy: a systematic review. J Oncol Pharm Pract 2023; 29(2): 358–69
[https://doi.org/10.1177/10781552211073576][PMID: 35048768]

[39]	Lasala R, Santoleri F. Association between adherence to oral therapies in cancer patients and clinical outcome: A systematic review of the literature. Br J Clin Pharmacol 2022; 88(5): 1999–2018
[https://doi.org/10.1111/bcp.15147][PMID: 34784654]

[40]	Lazzari N de, Niels T, Tewes M, Götte M. A Systematic Review of the Safety, Feasibility and Benefits of Exercise for Patients with Advanced Cancer. Cancers (Basel) 2021; 13(17)
[https://doi.org/10.3390/cancers13174478][PMID: 34503288]

[41]	Lee Y. Patients' perception and adherence to vaginal dilator therapy: a systematic review and synthesis employing symbolic interactionism. Patient Prefer Adherence 2018; 12: 551–60
[https://doi.org/10.2147/PPA.S163273][PMID: 29695897]

[42]	Lin C, Clark R, Tu P, Bosworth HB, Zullig LL. Breast cancer oral anti-cancer medication adherence: a systematic review of psychosocial motivators and barriers. Breast Cancer Res Treat 2017; 165(2): 247–60
[https://doi.org/10.1007/s10549-017-4317-2][PMID: 28573448]

[43]	Luo X, Chen Y, Chen J*, et al.* Effectiveness of mobile health-based self-management interventions in breast cancer patients: a meta-analysis. Support Care Cancer 2022; 30(3): 2853–76
[https://doi.org/10.1007/s00520-021-06568-0][PMID: 34561732]

[44]	Ma R-C, Yin Y-Y, Liu X, Wang Y-Q, Xie J. Effect of Exercise Interventions on Quality of Life in Patients With Lung Cancer: A Systematic Review of Randomized Controlled Trials. Oncol Nurs Forum 2020; 47(3): E58-E72
[https://doi.org/10.1188/20.ONF.E58-E72][PMID: 32301933]

[45]	Mathes T, Pieper D, Antoine S-L, Eikermann M. Adherence influencing factors in patients taking oral anticancer agents: a systematic review. Cancer Epidemiol 2014; 38(3): 214–26
[https://doi.org/10.1016/j.canep.2014.03.012][PMID: 24768601]

[46]	Mausbach BT, Schwab RB, Irwin SA. Depression as a predictor of adherence to adjuvant endocrine therapy (AET) in women with breast cancer: a systematic review and meta-analysis. Breast Cancer Res Treat 2015; 152(2): 239–46
[https://doi.org/10.1007/s10549-015-3471-7][PMID: 26077640]

[47]	McGrath D, O'Halloran P, Prue G*, et al.* Exercise Interventions for Women with Ovarian Cancer: A Realist Review. Healthcare (Basel) 2022; 10(4)
[https://doi.org/10.3390/healthcare10040720][PMID: 35455897]

[48]	McGrady ME, Pai ALH. A Systematic Review of Rates, Outcomes, and Predictors of Medication Non-Adherence Among Adolescents and Young Adults with Cancer. J Adolesc Young Adult Oncol 2019; 8(5): 485–94
[https://doi.org/10.1089/jayao.2018.0160][PMID: 31038372]

[49]	McHugh A, O'Connell E, Gurd B*, et al.* Mediterranean-style dietary interventions in adults with cancer: a systematic review of the methodological approaches, feasibility, and preliminary efficacy. Eur J Clin Nutr 2024; 78(6): 463–76
[https://doi.org/10.1038/s41430-024-01426-8][PMID: 38459161]

[50]	Molenaar CJ, van Rooijen SJ, Fokkenrood HJ, Roumen RM, Janssen L, Slooter GD. Prehabilitation versus no prehabilitation to improve functional capacity, reduce postoperative complications and improve quality of life in colorectal cancer surgery. Cochrane Database Syst Rev 2023; 5(5): CD013259
[https://doi.org/10.1002/14651858.CD013259.pub2][PMID: 35588252]

[51]	Moon Z, Moss-Morris R, Hunter MS, Carlisle S, Hughes LD. Barriers and facilitators of adjuvant hormone therapy adherence and persistence in women with breast cancer: a systematic review. Patient Prefer Adherence 2017; 11: 305–22
[https://doi.org/10.2147/PPA.S126651][PMID: 28260867]

[52]	Mullan LJ, Blackburn NE, Gracey J, Dunwoody L, Lorimer J, Semple CJ. Evaluating the effects of lymphoedema management strategies on functional status and health-related quality of life following treatment for head and neck cancer: a systematic review. J Cancer Surviv 2023; 19(1): 121–39
[https://doi.org/10.1007/s11764-023-01453-7][PMID: 37648875]

[53]	Murphy CC, Bartholomew LK, Carpentier MY, Bluethmann SM, Vernon SW. Adherence to adjuvant hormonal therapy among breast cancer survivors in clinical practice: a systematic review. Breast Cancer Res Treat 2012; 134(2): 459–78
[https://doi.org/10.1007/s10549-012-2114-5][PMID: 22689091]

[54]	Naser AY, Ofori-Asenso R, Awawdeh SA, Qadus S, Alwafi H, Liew D. Real World Adherence to and Persistence With Oral Oncolytics in Multiple Myeloma: A Systematic Review and Meta-analysis. Clin Lymphoma Myeloma Leuk 2022; 22(10): 760–73
[https://doi.org/10.1016/j.clml.2022.05.003][PMID: 35764491]

[55]	Noens L, Hensen M, Kucmin-Bemelmans I, Lofgren C, Gilloteau I, Vrijens B. Measurement of adherence to BCR-ABL inhibitor therapy in chronic myeloid leukemia: current situation and future challenges. Haematologica 2014; 99(3): 437–47
[https://doi.org/10.3324/haematol.2012.082511][PMID: 24598855]

[56]	Oldenmenger WH, Geerling JI, Mostovaya I*, et al.* A systematic review of the effectiveness of patient-based educational interventions to improve cancer-related pain. Cancer Treat Rev 2018; 63: 96–103
[https://doi.org/10.1016/j.ctrv.2017.12.005][PMID: 29272781]

[57]	Onvani S, Haghighatdoost F, Surkan PJ, Larijani B, Azadbakht L. Adherence to the Healthy Eating Index and Alternative Healthy Eating Index dietary patterns and mortality from all causes, cardiovascular disease and cancer: a meta-analysis of observational studies. J Hum Nutr Diet 2016; 30(2): 216–26
[https://doi.org/10.1111/jhn.12415][PMID: 27620213]

[58]	Ormel HL, van der Schoot GGF, Sluiter WJ, Jalving M, Gietema JA, Walenkamp AME. Predictors of adherence to exercise interventions during and after cancer treatment: A systematic review. Psychooncology 2018; 27(3): 713–24
[https://doi.org/10.1002/pon.4612][PMID: 29247584]

[59]	Paranjpe R, John G, Trivedi M, Abughosh S. Identifying adherence barriers to oral endocrine therapy among breast cancer survivors. Breast Cancer Res Treat 2019; 174(2): 297–305
[https://doi.org/10.1007/s10549-018-05073-z][PMID: 30523459]

[60]	Pezzolato M, Marzorati C, Lanzoni L*, et al.* "Interventions to increase adherence to oral therapies in breast cancer patients: A systematic review based on the behavior change technique taxonomy". Psychooncology 2023; 32(10): 1481–502
[https://doi.org/10.1002/pon.6203][PMID: 37571974]

[61]	Puspitasari Aw. Overview of Adherence and Influencing Factors to Capecitabine In Cancer Patients: Systematic Review. IJPR 2020; 13(01)
[https://doi.org/10.31838/ijpr/2021.13.01.476]

[62]	Puspitasari Aw. Digital Intervention to Improve Oral Chemotherapy Adherence: Systematic Review. IJPR 2021; 13(02)
[https://doi.org/10.31838/ijpr/2021.13.02.185]

[63]	Puspitasari Aw, Kristina SA, Satibi, Prabandari YS. Effect of Pharmacist Interventions on Medication Adherence to Capecitabine in Cancer Patients: A Systematic Review. Indonesian J Pharm 2022: 22–33
[https://doi.org/10.22146/ijp.2284]

[64]	Puts MTE, Tu HA, Tourangeau A*, et al.* Factors influencing adherence to cancer treatment in older adults with cancer: a systematic review. Ann Oncol 2014; 25(3): 564–77
[https://doi.org/10.1093/annonc/mdt433][PMID: 24285020]

[65]	Ramos PGF, Júdice PB, Nobre I, Carraça EV. Home-based exercise interventions' impact on breast cancer survivors' functional performance: a systematic review. J Cancer Surviv 2024
[https://doi.org/10.1007/s11764-024-01545-y][PMID: 38356019]

[66]	Schwedhelm C, Boeing H, Hoffmann G, Aleksandrova K, Schwingshackl L. Effect of diet on mortality and cancer recurrence among cancer survivors: a systematic review and meta-analysis of cohort studies. Nutr Rev 2016; 74(12): 737–48
[https://doi.org/10.1093/nutrit/nuw045][PMID: 27864535]

[67]	Setyowibowo H, Yudiana W, Hunfeld JAM*, et al.* Psychoeducation for breast cancer: A systematic review and meta-analysis. Breast 2022; 62: 36–51
[https://doi.org/10.1016/j.breast.2022.01.005][PMID: 35121502]

[68]	Skrabal Ross X, Gunn KM, Suppiah V, Patterson P, Olver I. A review of factors influencing non-adherence to oral antineoplastic drugs. Support Care Cancer 2020; 28(9): 4043–50
[https://doi.org/10.1007/s00520-020-05469-y][PMID: 32335731]

[69]	Sotirova MB, McCaughan EM, Ramsey L*, et al.* Acceptability of online exercise-based interventions after breast cancer surgery: systematic review and narrative synthesis. J Cancer Surviv 2021; 15(2): 281–310
[https://doi.org/10.1007/s11764-020-00931-6][PMID: 32930924]

[70]	Spei M-E, Bellos I, Samoli E, Benetou V. Post-Diagnosis Dietary Patterns among Cancer Survivors in Relation to All-Cause Mortality and Cancer-Specific Mortality: A Systematic Review and Meta-Analysis of Cohort Studies. Nutrients 2023; 15(17)
[https://doi.org/10.3390/nu15173860][PMID: 37686892]

[71]	Spence, Rosalind R. and Heesch, Kristiann and Brown, Wendy J. Exercise and cancer rehabilitation : a systematic review. Cancer Treat Rev 2010; (36(2)): 185–94.

[72]	Stalsberg R, Darvik MD. Social Representativeness and Intervention Adherence-A Systematic Review of Clinical Physical Activity Trials in Breast Cancer Patients. Int J Public Health 2024; 69: 1607002
[https://doi.org/10.3389/ijph.2024.1607002][PMID: 38784387]

[73]	Sun S, Zhang B, Zhang N, Zhang Y, Zhu M, Zhang M. Effect of telehealth interventions on adherence to endocrine therapy among patients with breast cancer: a systematic review and meta-analysis. Support Care Cancer 2024; 32(3): 151
[https://doi.org/10.1007/s00520-024-08339-z][PMID: 38332357]

[74]	Tan BK, Bee PC, Chua SS, Chen L-C. Monitoring and Improving Adherence to Tyrosine Kinase Inhibitors in Patients with Chronic Myeloid Leukemia: A Systematic Review. Patient Prefer Adherence 2021; 15: 2563–75
[https://doi.org/10.2147/PPA.S269355][PMID: 34819724]

[75]	Trauchburg A, Schwingshackl L, Hoffmann G. Association between Dietary Indices and Dietary Patterns and Mortality and Cancer Recurrence among Cancer Survivors: An Updated Systematic Review and Meta-Analysis of Cohort Studies. Nutrients 2023; 15(14)
[https://doi.org/10.3390/nu15143151][PMID: 37513568]

[76]	van Gestel T, Groen LCB, Puik JR*, et al.* Fit4Surgery for cancer patients during covid-19 lockdown - A systematic review and meta-analysis. Eur J Surg Oncol 2022; 48(6): 1189–97
[https://doi.org/10.1016/j.ejso.2022.02.010][PMID: 35183411]

[77]	van Liew JR, Christensen AJ, Moor JS de. Psychosocial factors in adjuvant hormone therapy for breast cancer: an emerging context for adherence research. J Cancer Surviv 2014; 8(3): 521–31
[https://doi.org/10.1007/s11764-014-0374-2][PMID: 24986227]

[78]	Verbrugghe M, Verhaeghe S, Lauwaert K, Beeckman D, van Hecke A. Determinants and associated factors influencing medication adherence and persistence to oral anticancer drugs: a systematic review. Cancer Treat Rev 2013; 39(6): 610–21
[https://doi.org/10.1016/j.ctrv.2012.12.014][PMID: 23428230]

[79]	Virani S, Afreen S, Perthiani A*, et al.* The Impact of Dietary Unsaturated Fat or the Mediterranean Diet on Women Diagnosed With Breast Cancer: A Systematic Review. Cureus 2024; 16(7): e65362
[https://doi.org/10.7759/cureus.65362][PMID: 39184716]

[80]	Visintini C, Mansutti I, Palese A. Medication adherence among allogeneic haematopoietic stem cell transplant recipients: a systematic review protocol. BMJ Open 2022; 12(12): e065676
[https://doi.org/10.1136/bmjopen-2022-065676][PMID: 36456011]

[81]	Wang Q-C, Yuan H, Chen Z-M, Wang J, Xue H, Zhang X-Y. Barriers and Facilitators of Adherence to Oral Nutritional Supplements Among People Living With Cancer: A Systematic Review. Clin Nurs Res 2023; 32(1): 209–20
[https://doi.org/10.1177/10547738221104216][PMID: 35707872]

[82]	Waseem H, Ginex PK, Sivakumaran K*, et al.* Interventions to Support Adherence to Oral Anticancer Medications: Systematic Review and Meta-Analysis. Oncol Nurs Forum 2022; 49(4): E4-E16
[https://doi.org/10.1188/22.ONF.E4-E16][PMID: 35788733]

[83]	Watts T, Courtier N, Fry S*, et al.* Access, acceptance and adherence to cancer prehabilitation: a mixed-methods systematic review. J Cancer Surviv 2024
[https://doi.org/10.1007/s11764-024-01605-3][PMID: 38709465]

[84]	Yang S, Park SW, Bae SJ, Ahn SG, Jeong J, Park K. Investigation of Factors Affecting Adherence to Adjuvant Hormone Therapy in Early-Stage Breast Cancer Patients: A Comprehensive Systematic Review. J Breast Cancer 2023; 26(4): 309–33
[https://doi.org/10.4048/jbc.2023.26.e22][PMID: 37272247]

[85]	Yussof I, Mohd Tahir NA, Hatah E, Mohamed Shah N. Factors influencing five-year adherence to adjuvant endocrine therapy in breast cancer patients: A systematic review. Breast 2022; 62: 22–35
[https://doi.org/10.1016/j.breast.2022.01.012][PMID: 35121501]

[86]	Zerillo JA, Goldenberg BA, Kotecha RR, Tewari AK, Jacobson JO, Krzyzanowska MK. Interventions to Improve Oral Chemotherapy Safety and Quality: A Systematic Review. JAMA Oncol 2018; 4(1): 105–17
[https://doi.org/10.1001/jamaoncol.2017.0625][PMID: 28570732]

[87]	Zhong Y, Zhu Y, Li Q*, et al.* Association between Mediterranean diet adherence and colorectal cancer: a dose-response meta-analysis. Am J Clin Nutr 2020; 111(6): 1214–25
[https://doi.org/10.1093/ajcn/nqaa083][PMID: 32359135]

[88]	Zou P, Huang A, Luo Y, Tchakerian N, Zhang H, Zhang C. Effects of using WeChat/WhatsApp on physical and psychosocial health outcomes among oncology patients: A systematic review. Health Informatics J 2023; 29(1): 14604582231164697
[https://doi.org/10.1177/14604582231164697][PMID: 36949645]

[89]	Zullig LL, Drake C, Shahsahebi M*, et al.* Adherence to cardiovascular disease risk factor medications among patients with cancer: a systematic review. J Cancer Surviv 2023; 17(3): 595–618
[https://doi.org/10.1007/s11764-022-01212-0][PMID: 35578150]
