# Supplementary material for: Patient-reported outcome measures (PROMs) capturing therapy adherence of cancer patients: an umbrella review
Source: Patient Relat Outcome Meas. 2026 May 12;17(1):25–34. doi: 10.1080/1179271X.2026.2669130 (PMC13536962; doi:10.1080/1179271X.2026.2669130)
Supplement: Supplementary file 4 [file DPRO_A_2669130_SM6251.docx]

**Table S2 Study characteristics of included Systematic Literature Reviews**

| **Systematic Literature Reviews** | **Studies with cancer patients included (N)** | **Studies usings PROMs included (N)** | **Patients included (N)** | **Meta analysis performed** | **Healthcare setting** | **study design** | **Cancer entities** | **Treatment stadium** | **Adherence Dimension focussed by SLR** |
| --- | --- | --- | --- | --- | --- | --- | --- | --- | --- |
| Abboud et al. 2021 [1] | 4 (plus 5 in non cancer-populations) | 2 | 205 | No | Multicontinental | RCT | Various cancer entities | Currently under treatment | Diet |
| Agirre-Elordui et al. 2024 [2] | 5 | 4 | 906 | Yes | Multicontinental | RCT | Colorectal / Gastrointestinal Cancers | Survivor | Exercise |
| Ahmed & Loiselle 2023 [3] | 120 | 30 | Not reported | No | Not reported | Multimethod | Various cancer entities | Currently under treatment | Medication |
| Alves et al. 2016 [4] | 14 | 6 | 2,043 | No | Multicontinental | Observational | Hematologic Malignancies | Currently under treatment | Medication |
| Atkinson et al. 2016 [5] | 8 | 8 | 813 | No | Not reported | Not reported | Various cancer entities | Currently under treatment | Medication |
| Barnes et al. 2019 [6] | 16 | 10 | Not reported for all studies | No | Multicontinental | Multimethod | Prostate Cancer | Currently under treatment, Survivor | Diet |
| Baydoun et al. 2021 [7] | 21 | 26 | 1,811 | No | Multicontinental | Multimethod | Various cancer entities | Currently under treatment, Survivor | Exercise |
| Bright et al. 2023 [8] | 33 | 17 | 375,951 | Yes | Multicontinental | Multimethod | Breast Cancer | Survivor | Medication |
| Bruns et al. 2016 [9] | 5 | 3 | 353 | No | Not reported | Multimethod | Colorectal / Gastrointestinal Cancers | Currently under treatment | Exercise |
| Burke et al. 2024 [10] | 12 | 2 | 1,461 | No | Multicontinental | Multimethod | Various cancer entities | Currently under treatment | Lifestyle |
| Cahir et al. 2015 [11] | 45 | 6 | 77,231 | Yes | Multicontinental | Multimethod | Breast Cancer | Currently under treatment | Medication |
| Carr & Rosengarten 2021 [12] | 7 | 1 | 416 | No | Multicontinental | Multimethod | Various cancer entities | Currently under treatment | Medication |
| Charters et al. 2024 [13] | 20 | 1 | 1,639 | No | Not reported | Multimethod | Head and Neck Cancer (HNC) | Currently under treatment | Exercise |
| Chen et al. 2022 [14] | 28 | 10 | 5,106 | No | Multicontinental | Multimethod | Colorectal / Gastrointestinal Cancers | Survivor | Diet |
| Chen et al. 2023 [15] | 11 | 14 | 9,140 | Yes | Multicontinental | Multimethod | Breast Cancer | Survivor | Diet |
| Cheung et al. 2023 [16] | 12 | 5 | 898 | No | Multicontinental | Multimethod | Various cancer entities | Currently under treatment | Exercise |
| Chlebowski & Geller 2006 [17] | 9 | 4 | 20,378 | No | Not reported | Multimethod | Breast Cancer | Currently under treatment | Medication |
| Chow et al. 2024 [18] | 4 | 4 | 806 | Yes | Multicontinental | RCT | Various cancer entities | Currently under treatment | Medication |
| Claros et al. 2019 [19] | 6 | 4 | 855 | No | Not reported | Not reported | Not reported | Currently under treatment | Medication |
| Deng et al. 2024 [20] | 34 | 1 | 31,696 | Yes | Multicontinental | Observational | Lung Cancer | Currently under treatment | Other |
| Eliasson, 2012 [21] | 17 | 2 | 5,450 | No | Multicontinental | Not reported | Hematologic Malignancies | Currently under treatment | Medication |
| Finitsis et al. 2018 [22] | 7 | 5 | 8,719 | Yes | Multicontinental | Multimethod | Breast Cancer | Survivor | Medication |
| Fleming et al. 2020 [23] | 62 | 30 | 78,321 | No | Multicontinental | Multimethod | Breast Cancer | Survivor | Medication |
| Ganna et al. 2024 [24] | 10 | 7 | 1,754 | No | Multicontinental | Multimethod | Breast Cancer | Currently under treatment, Survivor | Medication |
| Greer et al. 2016 [25] | 63 | 23 | 192,172 | No | Not reported | Multimethod | Various cancer entities | Currently under treatment, Survivor | Medication |
| Haddad et al. 2021 [26] | 28 | 6 | 3582 | No | Not reported | Multimethod | Gynecological Cancers | Currently under treatment, Survivor | Other |
| Hajjar & Rezazadeh, 2020 [27] | 9 | 8 | 68,5092 | No | Multicontinental | Observational | Various cancer entities | Currently under treatment, Survivor | Diet |
| Haynes et al. 2021 [28] | 5 | 4 | 3740 | Yes | Multicontinental | Observational | Head and Neck Cancer (HNC) | Currently under treatment | Lifestyle |
| Heiney et al. 2018 [29] | 16 | 9 | 60,159 | No | Multicontinental | Multimethod | Breast Cancer | Currently under treatment | Medication |
| Heiney et al. 2021 [30] | 14 | 2 | 6,177 | No | Not reported | Multimethod | Hematologic Malignancies | Currently under treatment | Medication |
| Horne et al. 2013 [31] | 3 (plus 91 in non cancer-populations) | 2 | 477 | No | Europe | Observational | Various cancer entities | Currently under treatment | Medication |
| Huang et al. 2016 [32] | 11 | 11 | 1,358 | No | Multicontinental | Observational | Various cancer entities | Currently under treatment | Medication |
| Hurtado-de-Medoza et al. 2016 [33] | 5 | 3 | 8,523 | No | Multicontinental | Multimethod | Breast Cancer | Survivor | Medication |
| Kaptein et al. 2021 [34] | 25 | 15 | 5,730 | No | Multicontinental | Not reported | Various cancer entities | Currently under treatment | Medication |
| Kavookjian & Wittayanukorn 2015 [35] | 6 | 1 | 537 | No | Multicontinental | Multimethod | Hematologic Malignancies | Currently under treatment | Medication |
| Kearney et al. 2022 [36] | 36 | 2 | 3,095 | No | Not reported | Multimethod | Various cancer entities | Currently under treatment | Exercise |
| Krekeler et al. 2018 [37] | 9 (plus 3 in non cancer populations) | 6 | 1,242 | No | Not reported | Multimethod | Head and Neck Cancer (HNC) | Currently under treatment | Other |
| Lafata et al. 2022 [38] | 24 | 7 | 3,943 | No | Multicontinental | Non-randomized Intervention | Various cancer entities | Currently under treatment | Medication |
| Lasala & Santoleri 2021 [39] | 42 | 13 | 22,109 | No | Not reported | Multimethod | Various cancer entities | Currently under treatment | Medication |
| Lazzari et al. 2021 [40] | 14 | 1 | 940 | No | Not reported | Multimethod | Various cancer entities | Currently under treatment | Exercise |
| Lee 2018 [41] | 12 (plus 8 in non-cancer populations) | 6 | 1,372 | No | Not reported | Multimethod | Gynecological Cancers | Currently under treatment | Other |
| Lin et al. 2017 [42] | 21 | 10 | 6,197 | No | Not reported | Multimethod | Breast Cancer | Currently under treatment, Survivor | Medication |
| Luo et al. 2022 [43] | 24 | 5 | 2,837 | Yes | Multicontinental | Multimethod | Breast Cancer | Currently under treatment | Exercise |
| Ma et al. 2020 [44] | 16 | 1 | 758 | No | Multicontinental | RCT | Lung Cancer | Currently under treatment | Exercise |
| Mathes et al. 2014 [45] | 22 | 7 | 36,960 | No | Multicontinental | Not reported | Various cancer entities | Currently under treatment | Medication |
| Mausbach, et al. 2015 [46] | 9 | 3 | 17,735 | Yes | Not reported | Not reported | Breast Cancer | Currently under treatment | Medication |
| Mc Grath et al. 2022 [47] | 9 | 5 | 396 | No | Multicontinental | Non-randomized Intervention | Gynecological Cancers | Currently under treatment, Survivor | Exercise |
| McGrady & Pai 2019 [48] | 12 | 2 | 1,651 | No | Not reported | RCT | Various cancer entities | Currently under treatment | Medication |
| McHugh et al. 2024 [49] | 14 | 14 | 2,783 | No | Multicontinental | RCT | Various cancer entities | Currently under treatment, Survivor | Diet |
| Molenaar et al. 2023 [50] | 3 | 3 | 250 | No | North America | RCT | Colorectal / Gastrointestinal Cancers | Currently under treatment | Multiple |
| Moon et al. 2017 [51] | 61 | 13 | 18,937 | No | Multicontinental | Observational | Breast Cancer | Currently under treatment | Medication |
| Mullan et al. 2023 [52] | 12 | 2 | 303 | No | Multicontinental | Multimethod | Head and Neck Cancer (HNC) | Currently under treatment | Medication |
| Murphy et al. 2012 [53] | 30 | 1 | 89,406 | No | Not reported | Observational | Breast Cancer | Survivor | Medication |
| Naser et al. 2022 [54] | 19 | 4 | 27,129 | Yes | Multicontinental | Not reported | Hematologic Malignancies | Currently under treatment | Medication |
| Noens et al. 2014 [55] | 19 | 8 | 3,345 | No | Multicontinental | Observational | Hematologic Malignancies | Currently under treatment | Medication |
| Oldenmenger et al. 2018 [56] | 26 | 5 | 4,735 | No | Multicontinental | RCT | Not reported | Currently under treatment | Medication |
| Onvani et al. 2016 [57] | 11 (plus 2 in non cancer-populations) | 11 | 922,199 | Yes | Multicontinental | Observational | Not reported | Currently under treatment | Diet |
| Ormel et al. 2017 [58] | 15 | 7 | 2,279 | No | Multicontinental | RCT | Various cancer entities | Currently under treatment, Survivor | Exercise |
| Parjanjpe et al. 2019 [59] | 19 | 7 | 7,007 | No | Europe | Observational | Breast Cancer | Survivor | Medication |
| Pezzolato et al. 2023 [60] | 36 | 20 | 28,528 | No | Multicontinental | Multimethod | Breast Cancer | Currently under treatment | Medication |
| Puspitasari et al. 2020 [61] | 9 | 6 | 997 | No | Multicontinental | Observational | Various cancer entities | Currently under treatment | Medication |
| Puspitasari et al. 2021 [62] | 6 | 4 | 504 | No | Not reported | Multimethod | Various cancer entities | Currently under treatment | Medication |
| Puspitasari et al. 2022 [63] | 5 | 1 | 301 | No | Multicontinental | Multimethod | Various cancer entities | Currently under treatment | Medication |
| Puts et al. 2014 [64] | 18 | 4 | 51,524 | No | Multicontinental | Multimethod | Various cancer entities | Currently under treatment | Medication |
| Ramos et al. 2024 [65] | 26 | 6 | 168 | No | Not reported | RCT | Breast Cancer | Currently under treatment, Survivor | Exercise |
| Schwedhelm et al. 2016 [66] | 117 | 54 | 209,597 | Yes | Multicontinental | Observational | Various cancer entities | Survivor | Diet |
| Setyowibowo et al. 2022 [67] | 27 | 1 | 7,742 | Yes | Multicontinental | RCT | Breast Cancer | Currently under treatment, Survivor | Other |
| Skrabal et al. 2021 [68] | 2 (plus 3 systematic reviews) | 2 | 2,639 | No | Not reported | Observational | Various cancer entities | Currently under treatment | Medication |
| Sotirova et al. 2021 [69] | 11 | 5 | 965 | No | Multicontinental | Multimethod | Breast Cancer | Currently under treatment, Survivor | Exercise |
| Spei et al. 2023 [70] | 19 | 19 | 38,846 | Yes | Multicontinental | Observational | Various cancer entities | Survivor | Diet |
| Spence et al. 2010 [71] | 10 | 3 | 505 | No | Not reported | Multimethod | Various cancer entities | Currently under treatment, Survivor | Exercise |
| Stalsberg & Darvik 2024 [72] | 37 | 16 | 2,042 | No | Multicontinental | RCT | Breast Cancer | Currently under treatment | Exercise |
| Sun et al. 2024 [73] | 8 | 5 | 1,780 | Yes | Multicontinental | RCT | Breast Cancer | Currently under treatment | Medication |
| Tan et al. 2021 [74] | 9 | 3 | 1,085 | No | Multicontinental | Multimethod | Hematologic Malignancies | Currently under treatment | Medication |
| Trauchburg et al. 2023 [75] | 23 (plus 18 studies by Schwedhelm et al. 2016 [66]) | 35 | 77,412 | Yes | Multicontinental | Observational | Various cancer entities | Survivor | Diet |
| van Gestel et al. 2022 [76] | 5 | 6 | 351 | Yes | Not reported | RCT | Various cancer entities | Currently under treatment | Exercise |
| van Liew et al. 2014 [77] | 14 | 4 | 22,798 | No | Multicontinental | Observational | Breast Cancer | Currently under treatment, Survivor | Medication |
| Verbrugghe et al. 2013 [78] | 25 | 12 | 55,530 | No | Multicontinental | Multimethod | Various cancer entities | Currently under treatment | Medication |
| Virani et al. 2024 [79] | 7 (plus 4 systematic reviews/ meta analyses) | 3 | Not reported for all studies | No | Not reported | Multimethod | Breast Cancer | Currently under treatment, Survivor | Diet |
| Visintini et al. 2023 [80] | 14 | 6 | 1,049 | No | Not reported | Multimethod | Not reported | Currently under treatment | Medication |
| Wang et al. 2023 [81] | 18 | 11 | 1,267 | No | Multicontinental | Multimethod | Various cancer entities | Currently under treatment | Medication |
| Waseem et al. 2022 [82] | 49 | 9 | 50,379 | Yes | Multicontinental | Multimethod | Various cancer entities | Currently under treatment | Medication |
| Watts et al. 2024 [83] | 56 | 11 | 3,500 | No | Multicontinental | Multimethod | Various cancer entities | Currently under treatment | Exercise |
| Yang et al. 2023 [84] | 146 | 49 | 50,6620 | No | Multicontinental | Multimethod | Breast Cancer | Currently under treatment, Survivor | Medication |
| Yussof et al. 2022 [85] | 26 | 3 | 140,254 | No | Multicontinental | Observational | Breast Cancer | Currently under treatment, Survivor | Medication |
| Zerillo et al. 2018 [86] | 16 | 4 | 3,612 | No | Multicontinental | Multimethod | Various cancer entities | Currently under treatment | Medication |
| Zhong et al. 2020 [87] | 13 | 13 | 1,607,886 | Yes | Multicontinental | Observational | Colorectal / Gastrointestinal Cancers | Currently under treatment | Diet |
| Zou et al. 2023 [88] | 20 | 1 | 3,110 | No | Multicontinental | Multimethod | Various cancer entities | Currently under treatment | Medication |
| Zullig et al. 2023 [89] | 21 | 4 | 2,666,401 | No | Multicontinental | Observational | Various cancer entities | Currently under treatment, Survivor | Medication |

Note. RCT = randomised controlled trial

References

[1]	Abboud M, AlAnouti F, Georgaki E, Papandreou D. Effect of Ketogenic Diet on Quality of Life in Adults with Chronic Disease: A Systematic Review of Randomized Controlled Trials. Nutrients 2021; 13(12)
[https://doi.org/10.3390/nu13124463][PMID: 34960015]

[2]	Agirre-Elordui S, Fernández-Landa J, Olasagasti-Ibargoien J, Castañeda-Babarro A. Physical activity maintenance in colorectal cancer survivors after an exercise intervention applying behaviour change techniques: a systematic review and meta-analysis. J Cancer Surviv 2024
[https://doi.org/10.1007/s11764-024-01654-8][PMID: 39098882]

[3]	Ahmed S, Loiselle CG. Patient Adherence to Oral Anticancer Agents: A Mapping Review of Supportive Interventions. Curr Oncol 2023; 30(12): 10224–36
[https://doi.org/10.3390/curroncol30120744][PMID: 38132378]

[4]	Alves AR, Lima WG, Nagai MM, Rodrigues JPV, Ayres LR. Adherence and/or discontinuation of imatinib mesylate in patients with chronic myeloid leukemia. Braz. J. Pharm. Sci. 2016; 52(4): 581–9
[https://doi.org/10.1590/S1984-82502016000400001]

[5]	Atkinson TM, Rodríguez VM, Gordon M*, et al.* The Association Between Patient-Reported and Objective Oral Anticancer Medication Adherence Measures: A Systematic Review. Oncol Nurs Forum 2016; 43(5): 576–82
[https://doi.org/10.1188/16.ONF.576-582][PMID: 27541550]

[6]	Barnes KA, Ball LE, Galvão DA, Newton RU, Chambers SK. Nutrition care guidelines for men with prostate cancer undergoing androgen deprivation therapy: do we have enough evidence? Prostate Cancer Prostatic Dis 2019; 22(2): 221–34
[https://doi.org/10.1038/s41391-018-0099-9][PMID: 30279584]

[7]	Baydoun M, Moran C, McLennan A, Piedalue K-AL, Oberoi D, Carlson LE. Mindfulness-Based Interventions in Cancer Survivors: A Systematic Review of Participants' Adherence to Home Practice. Patient Prefer Adherence 2021; 15: 1225–42
[https://doi.org/10.2147/PPA.S267064][PMID: 34135575]

[8]	Bright EE, Finkelstein LB, Nealis MS*, et al.* A Systematic Review and Meta-Analysis of Interventions to Promote Adjuvant Endocrine Therapy Adherence Among Breast Cancer Survivors. J Clin Oncol 2023; 41(28): 4548–61
[https://doi.org/10.1200/JCO.23.00697][PMID: 37531593]

[9]	Bruns ERJ, van den Heuvel B, Buskens CJ*, et al.* The effects of physical prehabilitation in elderly patients undergoing colorectal surgery: a systematic review. Colorectal Dis 2016; 18(8): O267-77
[https://doi.org/10.1111/codi.13429][PMID: 27332897]

[10]	Burke D, Brown M, O'Neill C*, et al.* The effect of lifestyle interventions on sarcopenia in advanced colorectal cancer: A systematic review. J Geriatr Oncol 2025; 16(1): 102143
[https://doi.org/10.1016/j.jgo.2024.102143][PMID: 39472241]

[11]	Cahir C, Guinan E, Dombrowski SU, Sharp L, Bennett K. Identifying the determinants of adjuvant hormonal therapy medication taking behaviour in women with stages I-III breast cancer: A systematic review and meta-analysis. Patient Educ Couns 2015
[https://doi.org/10.1016/j.pec.2015.05.013][PMID: 26054455]

[12]	Carr E, Rosengarten L. Teenagers and Young Adults with Cancer: An Exploration of Factors Contributing to Treatment Adherence. J Pediatr Oncol Nurs 2021; 38(3): 190–204
[https://doi.org/10.1177/1043454221992302][PMID: 33769898]

[13]	Charters E, Ricketts V, Sharman AR, Clark J. Systematic review of adherence to swallow and trismus exercises during radiation therapy for head and neck cancer. Head Neck 2024; 46(9): 2348–62
[https://doi.org/10.1002/hed.27849][PMID: 38895913]

[14]	Chen J, Jia L, Li Y*, et al.* Factors influencing adherence to healthy eating behaviors among adult colorectal cancer survivors: a systematic mixed method review. Support Care Cancer 2022; 31(1): 88
[https://doi.org/10.1007/s00520-022-07475-8][PMID: 36574042]

[15]	Chen G, Leary S, Niu J, Perry R, Papadaki A. The Role of the Mediterranean Diet in Breast Cancer Survivorship: A Systematic Review and Meta-Analysis of Observational Studies and Randomised Controlled Trials. Nutrients 2023; 15(9)
[https://doi.org/10.3390/nu15092099][PMID: 37432242]

[16]	Cheung C, Boocock E, Grande AJ, Maddocks M. Exercise-based interventions for cancer cachexia: A systematic review of randomised and non-randomised controlled trials. Asia Pac J Oncol Nurs 2023; 10(Suppl 1): 100335
[https://doi.org/10.1016/j.apjon.2023.100335][PMID: 38197041]

[17]	Chlebowski RT, Geller ML. Adherence to endocrine therapy for breast cancer. Oncology 2006; 71(1-2): 1–9
[https://doi.org/10.1159/000100444][PMID: 17344666]

[18]	Chow SM, Tan BK. Effectiveness of mHealth apps on adherence and symptoms to oral anticancer medications: a systematic review and meta-analysis. Support Care Cancer 2024; 32(7): 426
[https://doi.org/10.1007/s00520-024-08635-8][PMID: 38864924]

[19]	Claros MP, Messa CVM, García-Perdomo HA. Adherence to oral pharmacological treatment in cancer patients: Systematic review. Oncol Rev 2019; 13(1): 402
[https://doi.org/10.4081/oncol.2019.402][PMID: 31044026]

[20]	Deng T, Song J, Tuo J*, et al.* Incidence and risk factors of pulmonary complications after lung cancer surgery: A systematic review and meta-analysis. Heliyon 2024; 10(12): e32821
[https://doi.org/10.1016/j.heliyon.2024.e32821][PMID: 38975138]

[21]	Eliasson L. Treatment adherence in chronic myeloid leukemia: a systematic review of the literature. Clinical Practice 2012; 9(1): 87–100
[https://doi.org/10.2217/CPR.11.127]

[22]	Finitsis DJ, Vose BA, Mahalak JG, Salner AL. Interventions to promote adherence to endocrine therapy among breast cancer survivors: A meta-analysis. Psychooncology 2018; 28(2): 255–63
[https://doi.org/10.1002/pon.4959][PMID: 30511789]

[23]	Fleming L, Agnew S, Peddie N, Crawford M, Dixon D, MacPherson I. The impact of medication side effects on adherence and persistence to hormone therapy in breast cancer survivors: A quantitative systematic review. Breast 2022; 64: 63–84
[https://doi.org/10.1016/j.breast.2022.04.010][PMID: 35609380]

[24]	Ganna S, Rahimi S, Lu A, Laborde K, Trivedi M. Interventions to improve oral endocrine therapy adherence in breast cancer patients. J Cancer Surviv 2025; 19(3): 930–9
[https://doi.org/10.1007/s11764-023-01513-y][PMID: 38233637]

[25]	Greer JA, Amoyal N, Nisotel L*, et al.* A Systematic Review of Adherence to Oral Antineoplastic Therapies. Oncologist 2016; 21(3): 354–76
[https://doi.org/10.1634/theoncologist.2015-0405][PMID: 26921292]

[26]	Haddad NC, Soares Brollo LC, Pinho Oliveira MA, Bernardo-Filho M. Diagnostic Methods for Vaginal Stenosis and Compliance to Vaginal Dilator Use: A Systematic Review. J Sex Med 2021; 18(3): 493–514
[https://doi.org/10.1016/j.jsxm.2020.12.013][PMID: 33526400]

[27]	Hajjar M, Rezazadeh A. Recommended Food Score and Healthy Nordic Food Index in Cancer: A Systematic Review. Nutr Cancer 2021; 73(3): 369–75
[https://doi.org/10.1080/01635581.2020.1761406][PMID: 32400196]

[28]	Haynes DA, Vanison CC, Gillespie MB. The Impact of Dental Care in Head and Neck Cancer Outcomes: A Systematic Review and Meta-Analysis. Laryngoscope 2021; 132(1): 45–52
[https://doi.org/10.1002/lary.29494][PMID: 33635587]

[29]	Heiney SP, Parker PD, Felder TM, Adams SA, Omofuma OO, Hulett JM. A systematic review of interventions to improve adherence to endocrine therapy. Breast Cancer Res Treat 2018; 173(3): 499–510
[https://doi.org/10.1007/s10549-018-5012-7][PMID: 30387003]

[30]	Heiney SP, Sorrell M, Sheng J*, et al.* Interventions to Improve Adherence to Tyrosine Kinase Inhibitors in Chronic Myeloid Leukemia: A Systematic Review. Am J Clin Oncol 2021; 44(6): 291–8
[https://doi.org/10.1097/COC.0000000000000818][PMID: 33867480]

[31]	Horne R, Chapman SCE, Parham R, Freemantle N, Forbes A, Cooper V. Understanding patients' adherence-related beliefs about medicines prescribed for long-term conditions: a meta-analytic review of the Necessity-Concerns Framework. PLoS One 2013; 8(12): e80633
[https://doi.org/10.1371/journal.pone.0080633][PMID: 24312488]

[32]	Huang W-C, Chen C-Y, Lin S-J, Chang C-S. Medication adherence to oral anticancer drugs: systematic review. Expert Rev Anticancer Ther 2016; 16(4): 423–32
[https://doi.org/10.1586/14737140.2016.1159515][PMID: 26935964]

[33]	Hurtado-de-Mendoza A, Cabling ML, Lobo T, Dash C, Sheppard VB. Behavioral Interventions to Enhance Adherence to Hormone Therapy in Breast Cancer Survivors: A Systematic Literature Review. Clin Breast Cancer 2016; 16(4): 247-255.e3
[https://doi.org/10.1016/j.clbc.2016.03.006][PMID: 27133733]

[34]	Kaptein AA, Schoones JW, van der Meer PB*, et al.* Psychosocial determinants of adherence with oral anticancer treatment: 'we don't need no education'. Acta Oncol 2021; 60(1): 87–95
[https://doi.org/10.1080/0284186X.2020.1843190][PMID: 33151764]

[35]	Kavookjian J, Wittayanukorn S. Interventions for adherence with oral chemotherapy in hematological malignancies: A systematic review. Res Social Adm Pharm 2015; 11(3): 303–14
[https://doi.org/10.1016/j.sapharm.2014.08.006][PMID: 25262600]

[36]	Kearney N, Connolly D, Begic S, Mockler D, Guinan E. Feasibility metrics of exercise interventions during chemotherapy: A systematic review. Crit Rev Oncol Hematol 2024; 195: 104272
[https://doi.org/10.1016/j.critrevonc.2024.104272][PMID: 38272152]

[37]	Krekeler BN, Broadfoot CK, Johnson S, Connor NP, Rogus-Pulia N. Patient Adherence to Dysphagia Recommendations: A Systematic Review. Dysphagia 2018; 33(2): 173–84
[https://doi.org/10.1007/s00455-017-9852-9][PMID: 28965240]

[38]	Lafata JE, Nguyen B, Staresinic C, Johnson M, Gratie D, Muluneh B. Interpersonal communication-, education- and counselling-based interventions to support adherence to oral anticancer therapy: a systematic review. J Oncol Pharm Pract 2023; 29(2): 358–69
[https://doi.org/10.1177/10781552211073576][PMID: 35048768]

[39]	Lasala R, Santoleri F. Association between adherence to oral therapies in cancer patients and clinical outcome: A systematic review of the literature. Br J Clin Pharmacol 2022; 88(5): 1999–2018
[https://doi.org/10.1111/bcp.15147][PMID: 34784654]

[40]	Lazzari N de, Niels T, Tewes M, Götte M. A Systematic Review of the Safety, Feasibility and Benefits of Exercise for Patients with Advanced Cancer. Cancers (Basel) 2021; 13(17)
[https://doi.org/10.3390/cancers13174478][PMID: 34503288]

[41]	Lee Y. Patients' perception and adherence to vaginal dilator therapy: a systematic review and synthesis employing symbolic interactionism. Patient Prefer Adherence 2018; 12: 551–60
[https://doi.org/10.2147/PPA.S163273][PMID: 29695897]

[42]	Lin C, Clark R, Tu P, Bosworth HB, Zullig LL. Breast cancer oral anti-cancer medication adherence: a systematic review of psychosocial motivators and barriers. Breast Cancer Res Treat 2017; 165(2): 247–60
[https://doi.org/10.1007/s10549-017-4317-2][PMID: 28573448]

[43]	Luo X, Chen Y, Chen J*, et al.* Effectiveness of mobile health-based self-management interventions in breast cancer patients: a meta-analysis. Support Care Cancer 2022; 30(3): 2853–76
[https://doi.org/10.1007/s00520-021-06568-0][PMID: 34561732]

[44]	Ma R-C, Yin Y-Y, Liu X, Wang Y-Q, Xie J. Effect of Exercise Interventions on Quality of Life in Patients With Lung Cancer: A Systematic Review of Randomized Controlled Trials. Oncol Nurs Forum 2020; 47(3): E58-E72
[https://doi.org/10.1188/20.ONF.E58-E72][PMID: 32301933]

[45]	Mathes T, Pieper D, Antoine S-L, Eikermann M. Adherence influencing factors in patients taking oral anticancer agents: a systematic review. Cancer Epidemiol 2014; 38(3): 214–26
[https://doi.org/10.1016/j.canep.2014.03.012][PMID: 24768601]

[46]	Mausbach BT, Schwab RB, Irwin SA. Depression as a predictor of adherence to adjuvant endocrine therapy (AET) in women with breast cancer: a systematic review and meta-analysis. Breast Cancer Res Treat 2015; 152(2): 239–46
[https://doi.org/10.1007/s10549-015-3471-7][PMID: 26077640]

[47]	McGrath D, O'Halloran P, Prue G*, et al.* Exercise Interventions for Women with Ovarian Cancer: A Realist Review. Healthcare (Basel) 2022; 10(4)
[https://doi.org/10.3390/healthcare10040720][PMID: 35455897]

[48]	McGrady ME, Pai ALH. A Systematic Review of Rates, Outcomes, and Predictors of Medication Non-Adherence Among Adolescents and Young Adults with Cancer. J Adolesc Young Adult Oncol 2019; 8(5): 485–94
[https://doi.org/10.1089/jayao.2018.0160][PMID: 31038372]

[49]	McHugh A, O'Connell E, Gurd B*, et al.* Mediterranean-style dietary interventions in adults with cancer: a systematic review of the methodological approaches, feasibility, and preliminary efficacy. Eur J Clin Nutr 2024; 78(6): 463–76
[https://doi.org/10.1038/s41430-024-01426-8][PMID: 38459161]

[50]	Molenaar CJ, van Rooijen SJ, Fokkenrood HJ, Roumen RM, Janssen L, Slooter GD. Prehabilitation versus no prehabilitation to improve functional capacity, reduce postoperative complications and improve quality of life in colorectal cancer surgery. Cochrane Database Syst Rev 2023; 5(5): CD013259
[https://doi.org/10.1002/14651858.CD013259.pub2][PMID: 35588252]

[51]	Moon Z, Moss-Morris R, Hunter MS, Carlisle S, Hughes LD. Barriers and facilitators of adjuvant hormone therapy adherence and persistence in women with breast cancer: a systematic review. Patient Prefer Adherence 2017; 11: 305–22
[https://doi.org/10.2147/PPA.S126651][PMID: 28260867]

[52]	Mullan LJ, Blackburn NE, Gracey J, Dunwoody L, Lorimer J, Semple CJ. Evaluating the effects of lymphoedema management strategies on functional status and health-related quality of life following treatment for head and neck cancer: a systematic review. J Cancer Surviv 2023; 19(1): 121–39
[https://doi.org/10.1007/s11764-023-01453-7][PMID: 37648875]

[53]	Murphy CC, Bartholomew LK, Carpentier MY, Bluethmann SM, Vernon SW. Adherence to adjuvant hormonal therapy among breast cancer survivors in clinical practice: a systematic review. Breast Cancer Res Treat 2012; 134(2): 459–78
[https://doi.org/10.1007/s10549-012-2114-5][PMID: 22689091]

[54]	Naser AY, Ofori-Asenso R, Awawdeh SA, Qadus S, Alwafi H, Liew D. Real World Adherence to and Persistence With Oral Oncolytics in Multiple Myeloma: A Systematic Review and Meta-analysis. Clin Lymphoma Myeloma Leuk 2022; 22(10): 760–73
[https://doi.org/10.1016/j.clml.2022.05.003][PMID: 35764491]

[55]	Noens L, Hensen M, Kucmin-Bemelmans I, Lofgren C, Gilloteau I, Vrijens B. Measurement of adherence to BCR-ABL inhibitor therapy in chronic myeloid leukemia: current situation and future challenges. Haematologica 2014; 99(3): 437–47
[https://doi.org/10.3324/haematol.2012.082511][PMID: 24598855]

[56]	Oldenmenger WH, Geerling JI, Mostovaya I*, et al.* A systematic review of the effectiveness of patient-based educational interventions to improve cancer-related pain. Cancer Treat Rev 2018; 63: 96–103
[https://doi.org/10.1016/j.ctrv.2017.12.005][PMID: 29272781]

[57]	Onvani S, Haghighatdoost F, Surkan PJ, Larijani B, Azadbakht L. Adherence to the Healthy Eating Index and Alternative Healthy Eating Index dietary patterns and mortality from all causes, cardiovascular disease and cancer: a meta-analysis of observational studies. J Hum Nutr Diet 2016; 30(2): 216–26
[https://doi.org/10.1111/jhn.12415][PMID: 27620213]

[58]	Ormel HL, van der Schoot GGF, Sluiter WJ, Jalving M, Gietema JA, Walenkamp AME. Predictors of adherence to exercise interventions during and after cancer treatment: A systematic review. Psychooncology 2018; 27(3): 713–24
[https://doi.org/10.1002/pon.4612][PMID: 29247584]

[59]	Paranjpe R, John G, Trivedi M, Abughosh S. Identifying adherence barriers to oral endocrine therapy among breast cancer survivors. Breast Cancer Res Treat 2019; 174(2): 297–305
[https://doi.org/10.1007/s10549-018-05073-z][PMID: 30523459]

[60]	Pezzolato M, Marzorati C, Lanzoni L*, et al.* "Interventions to increase adherence to oral therapies in breast cancer patients: A systematic review based on the behavior change technique taxonomy". Psychooncology 2023; 32(10): 1481–502
[https://doi.org/10.1002/pon.6203][PMID: 37571974]

[61]	Puspitasari Aw. Overview of Adherence and Influencing Factors to Capecitabine In Cancer Patients: Systematic Review. IJPR 2020; 13(01)
[https://doi.org/10.31838/ijpr/2021.13.01.476]

[62]	Puspitasari Aw. Digital Intervention to Improve Oral Chemotherapy Adherence: Systematic Review. IJPR 2021; 13(02)
[https://doi.org/10.31838/ijpr/2021.13.02.185]

[63]	Puspitasari Aw, Kristina SA, Satibi, Prabandari YS. Effect of Pharmacist Interventions on Medication Adherence to Capecitabine in Cancer Patients: A Systematic Review. Indonesian J Pharm 2022: 22–33
[https://doi.org/10.22146/ijp.2284]

[64]	Puts MTE, Tu HA, Tourangeau A*, et al.* Factors influencing adherence to cancer treatment in older adults with cancer: a systematic review. Ann Oncol 2014; 25(3): 564–77
[https://doi.org/10.1093/annonc/mdt433][PMID: 24285020]

[65]	Ramos PGF, Júdice PB, Nobre I, Carraça EV. Home-based exercise interventions' impact on breast cancer survivors' functional performance: a systematic review. J Cancer Surviv 2024
[https://doi.org/10.1007/s11764-024-01545-y][PMID: 38356019]

[66]	Schwedhelm C, Boeing H, Hoffmann G, Aleksandrova K, Schwingshackl L. Effect of diet on mortality and cancer recurrence among cancer survivors: a systematic review and meta-analysis of cohort studies. Nutr Rev 2016; 74(12): 737–48
[https://doi.org/10.1093/nutrit/nuw045][PMID: 27864535]

[67]	Setyowibowo H, Yudiana W, Hunfeld JAM*, et al.* Psychoeducation for breast cancer: A systematic review and meta-analysis. Breast 2022; 62: 36–51
[https://doi.org/10.1016/j.breast.2022.01.005][PMID: 35121502]

[68]	Skrabal Ross X, Gunn KM, Suppiah V, Patterson P, Olver I. A review of factors influencing non-adherence to oral antineoplastic drugs. Support Care Cancer 2020; 28(9): 4043–50
[https://doi.org/10.1007/s00520-020-05469-y][PMID: 32335731]

[69]	Sotirova MB, McCaughan EM, Ramsey L*, et al.* Acceptability of online exercise-based interventions after breast cancer surgery: systematic review and narrative synthesis. J Cancer Surviv 2021; 15(2): 281–310
[https://doi.org/10.1007/s11764-020-00931-6][PMID: 32930924]

[70]	Spei M-E, Bellos I, Samoli E, Benetou V. Post-Diagnosis Dietary Patterns among Cancer Survivors in Relation to All-Cause Mortality and Cancer-Specific Mortality: A Systematic Review and Meta-Analysis of Cohort Studies. Nutrients 2023; 15(17)
[https://doi.org/10.3390/nu15173860][PMID: 37686892]

[71]	Spence, Rosalind R. and Heesch, Kristiann and Brown, Wendy J. Exercise and cancer rehabilitation : a systematic review. Cancer Treat Rev 2010; (36(2)): 185–94.

[72]	Stalsberg R, Darvik MD. Social Representativeness and Intervention Adherence-A Systematic Review of Clinical Physical Activity Trials in Breast Cancer Patients. Int J Public Health 2024; 69: 1607002
[https://doi.org/10.3389/ijph.2024.1607002][PMID: 38784387]

[73]	Sun S, Zhang B, Zhang N, Zhang Y, Zhu M, Zhang M. Effect of telehealth interventions on adherence to endocrine therapy among patients with breast cancer: a systematic review and meta-analysis. Support Care Cancer 2024; 32(3): 151
[https://doi.org/10.1007/s00520-024-08339-z][PMID: 38332357]

[74]	Tan BK, Bee PC, Chua SS, Chen L-C. Monitoring and Improving Adherence to Tyrosine Kinase Inhibitors in Patients with Chronic Myeloid Leukemia: A Systematic Review. Patient Prefer Adherence 2021; 15: 2563–75
[https://doi.org/10.2147/PPA.S269355][PMID: 34819724]

[75]	Trauchburg A, Schwingshackl L, Hoffmann G. Association between Dietary Indices and Dietary Patterns and Mortality and Cancer Recurrence among Cancer Survivors: An Updated Systematic Review and Meta-Analysis of Cohort Studies. Nutrients 2023; 15(14)
[https://doi.org/10.3390/nu15143151][PMID: 37513568]

[76]	van Gestel T, Groen LCB, Puik JR*, et al.* Fit4Surgery for cancer patients during covid-19 lockdown - A systematic review and meta-analysis. Eur J Surg Oncol 2022; 48(6): 1189–97
[https://doi.org/10.1016/j.ejso.2022.02.010][PMID: 35183411]

[77]	van Liew JR, Christensen AJ, Moor JS de. Psychosocial factors in adjuvant hormone therapy for breast cancer: an emerging context for adherence research. J Cancer Surviv 2014; 8(3): 521–31
[https://doi.org/10.1007/s11764-014-0374-2][PMID: 24986227]

[78]	Verbrugghe M, Verhaeghe S, Lauwaert K, Beeckman D, van Hecke A. Determinants and associated factors influencing medication adherence and persistence to oral anticancer drugs: a systematic review. Cancer Treat Rev 2013; 39(6): 610–21
[https://doi.org/10.1016/j.ctrv.2012.12.014][PMID: 23428230]

[79]	Virani S, Afreen S, Perthiani A*, et al.* The Impact of Dietary Unsaturated Fat or the Mediterranean Diet on Women Diagnosed With Breast Cancer: A Systematic Review. Cureus 2024; 16(7): e65362
[https://doi.org/10.7759/cureus.65362][PMID: 39184716]

[80]	Visintini C, Mansutti I, Palese A. Medication adherence among allogeneic haematopoietic stem cell transplant recipients: a systematic review protocol. BMJ Open 2022; 12(12): e065676
[https://doi.org/10.1136/bmjopen-2022-065676][PMID: 36456011]

[81]	Wang Q-C, Yuan H, Chen Z-M, Wang J, Xue H, Zhang X-Y. Barriers and Facilitators of Adherence to Oral Nutritional Supplements Among People Living With Cancer: A Systematic Review. Clin Nurs Res 2023; 32(1): 209–20
[https://doi.org/10.1177/10547738221104216][PMID: 35707872]

[82]	Waseem H, Ginex PK, Sivakumaran K*, et al.* Interventions to Support Adherence to Oral Anticancer Medications: Systematic Review and Meta-Analysis. Oncol Nurs Forum 2022; 49(4): E4-E16
[https://doi.org/10.1188/22.ONF.E4-E16][PMID: 35788733]

[83]	Watts T, Courtier N, Fry S*, et al.* Access, acceptance and adherence to cancer prehabilitation: a mixed-methods systematic review. J Cancer Surviv 2024
[https://doi.org/10.1007/s11764-024-01605-3][PMID: 38709465]

[84]	Yang S, Park SW, Bae SJ, Ahn SG, Jeong J, Park K. Investigation of Factors Affecting Adherence to Adjuvant Hormone Therapy in Early-Stage Breast Cancer Patients: A Comprehensive Systematic Review. J Breast Cancer 2023; 26(4): 309–33
[https://doi.org/10.4048/jbc.2023.26.e22][PMID: 37272247]

[85]	Yussof I, Mohd Tahir NA, Hatah E, Mohamed Shah N. Factors influencing five-year adherence to adjuvant endocrine therapy in breast cancer patients: A systematic review. Breast 2022; 62: 22–35
[https://doi.org/10.1016/j.breast.2022.01.012][PMID: 35121501]

[86]	Zerillo JA, Goldenberg BA, Kotecha RR, Tewari AK, Jacobson JO, Krzyzanowska MK. Interventions to Improve Oral Chemotherapy Safety and Quality: A Systematic Review. JAMA Oncol 2018; 4(1): 105–17
[https://doi.org/10.1001/jamaoncol.2017.0625][PMID: 28570732]

[87]	Zhong Y, Zhu Y, Li Q*, et al.* Association between Mediterranean diet adherence and colorectal cancer: a dose-response meta-analysis. Am J Clin Nutr 2020; 111(6): 1214–25
[https://doi.org/10.1093/ajcn/nqaa083][PMID: 32359135]

[88]	Zou P, Huang A, Luo Y, Tchakerian N, Zhang H, Zhang C. Effects of using WeChat/WhatsApp on physical and psychosocial health outcomes among oncology patients: A systematic review. Health Informatics J 2023; 29(1): 14604582231164697
[https://doi.org/10.1177/14604582231164697][PMID: 36949645]

[89]	Zullig LL, Drake C, Shahsahebi M*, et al.* Adherence to cardiovascular disease risk factor medications among patients with cancer: a systematic review. J Cancer Surviv 2023; 17(3): 595–618
[https://doi.org/10.1007/s11764-022-01212-0][PMID: 35578150]
